# Identification of downstream targets and signaling pathways of long non-coding RNA NR_002794 in human trophoblast cells

**DOI:** 10.1080/21655979.2021.1974808

**Published:** 2021-09-13

**Authors:** Yinyao Ma, Hua Wu, Xuxia Liang, Chun Zhang, Yanhua Ma, Yanfen Wei, Jing Li, Hui Chen

**Affiliations:** Department of Obstetrics, People’s Hospital of Guangxi Zhuang Autonomous Region, Nanning, P.R. China

**Keywords:** Preeclampsia, trophoblast, NR_002794, TIE1, AKT, ERK

## Abstract

Preeclampsia (PE) is a huge threat to pregnant women. Our previous study demonstrated that long non-coding RNA (lncRNA) NR_002794 was highly expressed in placentas of PE patients and could regulate the phenotypes of trophoblast cells. However, the downstream regulatory mechanisms of NR_002794 remain unknown. In this text, some potential downstream targets or signaling pathways of NR_002794 were identified through RNA sequencing (RNA-seq) and bioinformatics analysis in SWAN71 trophoblast cells. Western blot assay demonstrated that NR_002794 inactivated protein kinase B (AKT) and extracellular signal-regulated kinase 1/2 (ERK1/2) pathways and activated cell apoptotic signaling in SWAN71 cells. Both RNA-seq and reverse transcription-quantitative PCR (RT-qPCR) outcomes showed that NR_002794 up-regulation could notably inhibit the expression of C-C motif chemokine ligand 4 like 2 (CCL4L2), interleukin 15 receptor subunit alpha (IL15RA), interleukin 32 (IL32), and tyrosine kinase with immunoglobulin-like and EGF-like domains 1 (TIE1), while NR_002794 knockdown induced these gene expressions in SWAN71 cells. CCK-8, BrdU, Transwell, wound healing, and flow cytometry analyses showed that NR_002794 inhibited cell proliferation and migration and induced cell apoptosis through down-regulating TIE1 in SWAN71 cells. In conclusion, lncRNA NR_002794 could exert its functions by regulating AKT and ERK1/2 pathways and TIE1 expression in human trophoblast cells.

## Introduction

Preeclampsia (PE), a pregnancy-related multisystem disease that is characterized by hypertension and proteinuria, can affect 0.2%–9.2% of pregnant women [1]. It is a major cause of maternal mortality and morbidity, premature delivery, and neonatal and fetal death [2,3]. It is estimated that more than 70,000 maternal deaths and 500,000 infant deaths per year can be attributed to PE [4]. Moreover, PE is closely linked with the increased risks of cerebrovascular, cardiovascular, and end-stage renal diseases and multi-organ dysfunction [2,5]. Despite the major advances in the management of PE over the past decades, accurate/effective screening, diagnosis, or treatment strategies for PE remain seriously lacking [2]. Trophoblast cells, an essential cell type on the outer membrane of the placenta, play vital roles in fetal-maternal exchange, blastocyst implantation, and placentation [6,7]. Many studies support that abnormal trophoblast development is closely associated with the pathogenesis of PE [8,9]. However, the molecules or pathways governing trophoblast development are largely unknown. An in-depth insight into the molecular mechanisms underlying trophoblast dysfunction might contribute to the better management of PE.

Long non-coding RNAs (lncRNAs), a group of long (>200 nucleotides in length) transcripts with little or no protein-coding potential, have been identified as vital players in various physiopathological processes [10,11]. Moreover, lncRNAs can exert their functions by altering downstream gene expression and regulating downstream signaling pathways [12,13]. Over the past decades, multiple lncRNAs have been found to be dysregulated in preeclamptic placentas and dysregulated lncRNAs have been demonstrated to be able to alter the behaviors and phenotypes of trophoblast cells such as proliferation, apoptosis, and migration [14,15]. For instance, lncRNA Prospero homeobox 1-antisense RNA 1 (PROX1-AS1) expression level was notably increased in the placental tissues and blood samples of the patients with PE, and PROX1-AS1 loss facilitated cell migration and invasion and suppressed cell apoptosis by regulating microRNA-211-5p/caspase9 axis in human trophoblast cells [16]. LncRNA SH3PXD2A antisense RNA 1 (SH3PXD2A-AS1) was highly expressed in the placentas of PE patients, and SH3PXD2A-AS1 overexpression weakened cell proliferative, migratory, and invasive abilities and induced cell apoptosis by negatively regulating the expression of SH3 and PX domains 2A and C-C motif chemokine receptor 7 in human trophoblast cells [17].

Our recent study showed that lncRNA NR_002794 was highly expressed in the placental tissues of patients with PE, and enforced expression of NR_002794 triggered the notable reduction of cell proliferative and migratory abilities and marked increase of cell apoptotic rate in SWAN71 trophoblast cells [18]. However, the molecular mechanisms underlying the role of NR_002794 in the development of trophoblast cells remain unknown.

In this text, downstream targets and signaling pathways of NR_002794 were investigated by high throughput RNA sequencing (RNA-seq) technology, KEGG enrichment analysis, and cellular/molecular experiments in SWAN71 human trophoblast cells. We supposed that the pathways that were significantly enriched by the commonly dysregulated genes after NR_002794 overexpression or knockdown could be regulated by NR_002794 in SWAN71 cells. Among these pathways, we further examined the effects of NR_002794 up-regulation or down-regulation on protein kinase B (AKT) and extracellular signal-regulated kinase 1/2 (ERK1/2), Bcl-2/caspase3 pathways in SWAN71 cells. Moreover, we supposed that differentially expressed genes with converse alteration trends after NR_002794 overexpression or knockdown had a high possibility to be the potential targets of NR_002794. Subsequently, 9 potential NR_002794 targets were screened out for further reverse transcription-quantitative PCR (RT-qPCR) validation. Among genes with consistent outcomes in RT-qPCR and RNA-seq analyses, we further investigated whether NR_002794 could exert its functions by targeting tyrosine kinase with immunoglobulin-like and EGF-like domains 1 (TIE1) in SWAN71 cells.

## Materials and methods

### Cell culture and cell transfection

SWAN71 human trophoblast cells (kindly provided by Professor Ke Wu, School of Life Sciences, Peking University, Beijing, China) and HEK293T cells (obtained from American Type Culture Collection, Rockville, MD, USA) were cultured in DMEM medium (Thermo Scientific, Waltham, MA, USA) containing 10% fetal bovine serum (Thermo Scientific) in a 5% CO_2_ incubator at 37°C. Plasmids were transfected into HEK293T or SWAN71 cells using the Lipofectamine 2000 reagent (Thermo Scientific).

### Recombinant plasmid construction

To silence NR_002794, 3 pairs of oligonucleotides targeting NR_002794 were constructed into the pLenti6.3-shRNA-GFP vector (Novobio Biotechnology Co., Ltd., Shanghai, China) and generated recombinant plasmids were named sh-NR_002794#1, sh-NR_002794#2, and sh-NR_002794#3. The full-length fragment of NR_002794 was subcloned into the lentiviral vector pL6.3-CMV-GFPa1-IRES-MCS (Novobio Biotechnology). The coding region of TIE1 was constructed into the pLenti6.3-MCS lentiviral vector (Novobio Biotechnology) to generate the lenti-TIE1 lentiviral overexpression plasmid. The sequences of the above-mentioned oligonucleotides or primers were presented in Table 1.

**Table 1.** The sequences of the oligos or primers

The sequences of 3 pairs of oligos targeting NR_002794					
**name**			**sequences**		
sh-NR_002794#1-sense (5ʹ-3ʹ)			CACCGGTGGTCAACATCACCATGGTCGAAACCATGGTGATGTTGACCACC		
sh-NR_002794#1-antisense (5ʹ-3ʹ)			AAAAGGTGGTCAACATCACCATGGTTTCGACCATGGTGATGTTGACCACC		
sh-NR_002794#2-sense (5ʹ-3ʹ)			CACCGTGCTTCTGTAAGGGCTACAACGAATTGTAGCCCTTACAGAAGCAC		
sh-NR_002794#2-antisense (5ʹ-3ʹ)			AAAAGTGCTTCTGTAAGGGCTACAATTCGTTGTAGCCCTTACAGAAGCAC		
sh-NR_002794#3-sense (5ʹ-3ʹ)			CACCGGCAAGGTTTGGTGCAAAATCCGAAGATTTTGCACCAAACCTTGCC		
sh-NR_002794#3-antisense (5ʹ-3ʹ)			AAAAGGCAAGGTTTGGTGCAAAATCTTCGGATTTTGCACCAAACCTTGCC		
**Overexpression primer sequences**					
**name**	**forward primer (5ʹ-3ʹ)**			**reverse primer (5ʹ-3ʹ)**	
TIE1	AATTAAGGAAGCTAGCatggtctggcgggtgc			TAAATCCAAGGCGCGCCtcaggcctcctcagctgtggc	
**QPCR primer sequences**					
**gene name**		**foward(5ʹ-3ʹ)**			**revese(5ʹ-3ʹ)**
NR_002794		TCTGTCTGTGCAGTGCTTCTG			GTCGTCCTGCAGCAAGTAGC
CCL4L2		CCGCCTGCTGCTTTTCTTAC			TTGCTTGCCTACCACAGC
IL15RA		CCCAGCTCAAACAACACAGC			AGGTAGCATGCCAGGAGAGA
IL32		AGAGGGCTACCTGGAGACAG			CACCACCTTCTCCTTCACCC
TIE1		AGAGCATGGGACAGCCTCTA			CCAGGTCCCTGTGGATGAAC
Dkk1		GCCTCAGGATTGTGTTGTGC			ATCCGGCAAGACAGACCTTC
DMD		TCTACAGAGGTCCGACAGCA			CTCATTGGCTTTCCAGGGGT
GCNT1		TATCTCTGGGCCACCATCCA			GTTCAAGTCACCAGCTCCGA
Gypc		AGCCTGATCCAGGGATGTCT			TGCATCTGCACTCTCAGCAA
Hes1		GTGTCAACACGACACCGGAT			GGAATGCCGCGAGCTATCTT

### Lentiviral package

Lentiviral particles were packaged as previously described [19]. Briefly, the recombinant lentiviral plasmid and packaging plasmid mix (Novobio Biotechnology) were co-transfected into HEK293T cells. At 60 h after transfection, cell supernatants containing lentiviral particles were collected and titrated.

### Reverse transcription-quantitative PCR (RT-qPCR) assay

The RT-qPCR assay was performed as previously described [20,21]. Briefly, RNA was isolated from SWAN71 cells using the Trizol reagent (Thermo Scientific) and then reversely transcribed into the cDNA first-strand using the M-MLV Reverse Transcriptase (Thermo Scientific). Next, cDNA was amplified and quantified using the SYBR Green PCR Master Mix (Thermo Scientific) and specific quantitative primers. Relative quantification analysis was performed using the 2^−ΔΔCt^ method. The quantitative primer sequences were shown in Table 1.

### RNA-seq

RNA was extracted from SWAN71 cells infected with lenti-NR_002794 (OE group), sh-NR_002794 (KD group), or control (NC group) lentiviruses as described above. RNA containing polyA structure was enriched using the Oligo(dT) magnetic beads and then broken into approximately 300 bp fragments. Next, the cDNA library (about 450 bp) was constructed using the TruSeq RNA Library Prep Kit (Illumina, San Diego, CA, USA) and sequenced on Illumina HiSeq 2500 instrument (Illumina). The sequences after quality control were aligned to the reference genome (Homo_sapiens.GRCh38.dna.primary_assembly.fa) using the HISAT2 software. Gene expression levels were normalized using the TPM (Transcripts Per Million) method. Differential analysis was performed using the DEGseq software. Genes were considered as differentially expressed at |log_2_FoldChange| > 1 and *q* value < 0.05. KEGG enrichment analysis and gene annotation analysis were conducted using the KOBAS3.0 online website (http://kobas.cbi.pku.edu.cn/kobas3/?t=1). Subcluster analysis was carried out using the R gplots.

### Western blot assay

Protein expression levels were determined by western blot assay as previously described [20,22]. Protein was extracted from SWAN71 cells using the RIPA lysis buffer (Beyotime Biotechnology, Shanghai, China) supplemented with Protease and Phosphatase Inhibitor Cocktail (Thermo Scientific), and then quantified using the Pierce BCA Protein Assay Kit (Thermo Scientific). Next, protein (30 μg per lane) was separated through sodium dodecyl sulfate-polyacrylamide gel electrophoresis and transferred onto nitrocellulose membranes (Millipore, Bedford, MA, USA). After the blockade of nonspecific signals, the membranes were incubated for 12 h at 4°C with anti-pan-protein kinase B (AKT) (1:1000 dilution, ab8805, Abcam), anti-phosphorylated AKT (1:1000 dilution, ab38449, Abcam, Cambridge, UK), anti-extracellular signal-regulated kinase 1/2 (ERK1/2) (1:1000 dilution, ab184699, Abcam), anti-phosphorylated ERK1/2 (1:1000 dilution, ab214036, Abcam), anti-Cleaved Caspase-3 (1:1000 dilution, ab32042, Abcam), anti-Bcl2 (1:1000 dilution, ab32124, Abcam), or anti-GAPDH (1:2000 dilution, ab8245, Abcam). Next, the membranes were probed with horseradish peroxidase-conjugated secondary antibody for 1 h at room temperature. Finally, protein signals were detected using the Pierce ECL Western Blotting Substrate (Thermo Fisher Scientific) and quantified using the Image J v1.8.0 software (National Institutes of Health, Bethesda, MD, USA).

### CCK-8 assay

The proliferative activity of SWAN71 cells was measured using the Cell Counting Kit-8 (MedChemExpress, Monmouth Junction, NJ, USA) following the protocols of the manufacturer as previously described [20]. Briefly, cells were seeded into 96-well plates and infected with corresponding lentiviruses. After 48 h of incubation, 10 μl of CCK-8 solution was added to each well. Two hours later, the optical density (OD) values were measured at 450 nm.

### Bromodeoxyuridine (BrdU) assay

BrdU assay was carried out as previously described [23]. Briefly, cells were co-incubated with BrdU solution (10 μM) for 1 h. Subsequently, cells were fixed with 4% paraformaldehyde for 20 min at room temperature, permeabilized with 0.25% Triton X-100 for 20 min at room temperature, treated with 2 M HCl for 30 min at 37°C, and washed using phosphate/citric acid buffer (pH = 7.4) for 5 min. Next, cells were incubated with the antibody against BrdU for over 12 h at 4°C and corresponding fluorescence-labeled secondary antibody for 45 min at 37°C. After the treatment of DAPI solution, cells were imaged using a flow cytometer.

### Cell migration assay

Transwell assay was conducted as previously described [24,25]. Briefly, cells infected with matching lentiviruses were resuspended in serum-free medium and inoculated into the upper chambers of the 8 µm pore-size transwell plates (Corning Inc., New York, NY, USA), and medium supplemented with 10% FBS was added into the lower chambers. After 24 h of incubation in a 5% CO_2_ incubator at 37°C, cells migrated to the bottom side of the membranes were fixed with 4% paraformaldehyde and stained with crystal violet solution. Next, cells were imaged using a microscope and counted in 5 random fields.

### Wound healing assay

Wound healing assay was carried out as previously described [25–27]. Briefly, a vertical wound was created using a pipette tip when cells reached 100% confluence. Next, cells in serum-free medium were allowed to grow for 24 h. The wound area was imaged at 0 h and 24 h under a microscope.

## Cell apoptotic percentage analysis

Cell apoptotic percentage was examined using the Annexin V-FITC Apoptosis Detection Kit (Beyotime Biotechnology) following the protocols of the manufacturer as previously described [28,29]. Briefly, cells were collected at 72 h after lentiviral infection. Next, cells were re-suspended in Annexin V-FITC binding solution and co-incubated with Annexin V-FITC and Propidium Iodide solutions for 20 min at room temperature in the dark. Cell apoptotic percentage was measured using flow cytometry (BD Biosciences, San Diego, CA, USA).

## Statistics analysis

Data were analyzed using GraphPad Prism software (version 7, GraphPad Software, Inc., San Diego, CA, USA) with the results shown as means ± standard deviation. Difference analysis among groups was performed using one-way analysis of variance (ANOVA) together with Tukey’s post-hoc test with *P* < 0.05 as statistically significant.

## Results

Our previous study demonstrated that lncRNA NR_002794 was aberrantly expressed in the placental tissues of patients with PE and NR_002794 overexpression could influence the development of trophoblasts [18]. However, the downstream targets and regulatory pathways of NR_002794 are poorly defined. In this text, RNA-seq technology was used to identify dysregulated genes in OE versus NC group or KD versus NC group. The potential signaling pathways downstream of NR_002794 were identified by KEGG enrichment analysis for the dysregulated genes in both OE versus NC group and KD versus NC group. Among the pathways that could be regulated by NR_002794, we further investigated the effects of NR_002794 overexpression or knockdown on AKT, and ERK1/2 pathways. Our prior study demonstrated that NR_002794 could induce cell apoptosis in SWAN71 cells. Thus, the influences of NR_002794 up-regulation or down-regulation on the Bcl-2/caspase3 apoptotic pathway were also examined. We believed that differentially expressed genes with converse alteration trends in OE versus NC group and KD versus NC group might be the potential targets of NR_002794. Next, 9 potential NR_002794 targets were picked out for further RT-qPCR validation. Among genes with consistent outcomes in RNA-seq and RT-qPCR analyses, we further explored whether NR_002794 could influence cell proliferation, migration, and apoptosis by regulating TIE1 in SWAN71 cells.

### Identification of NR_002794 downstream genes and signaling pathways through RNA-seq and bioinformatics analysis

**Figure 1.**
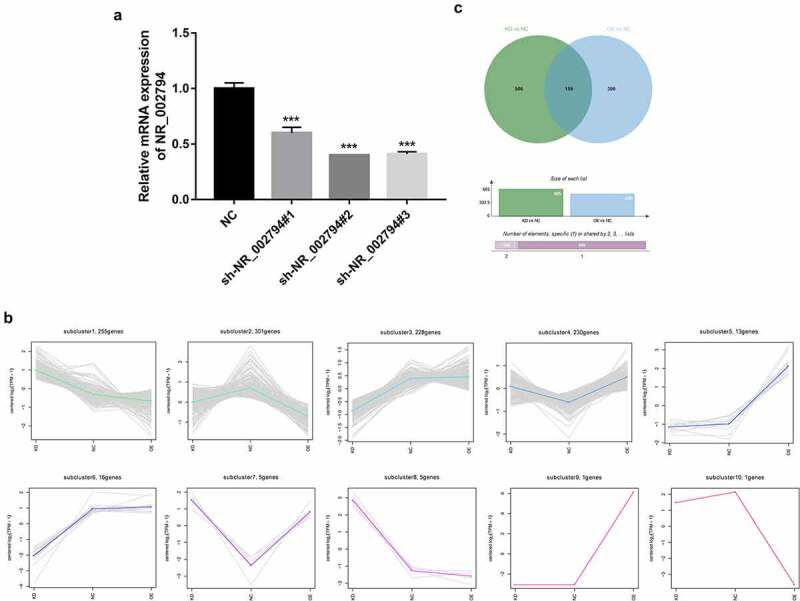
**Identification of NR_002794 downstream genes and signaling pathways through RNA-seq and bioinformatics analysis**. (a) SWAN71 cells were transfected with sh-NR_002794#1, sh-NR_002794#2, or sh-NR_002794#3 knockdown plasmid or control plasmid. Forty-eight hours later, the NR_002794 level was measured by RT-qPCR assay. (b) Cluster analysis for differentially expressed genes in KD versus NC group and OE versus NC group. (c) Venn analysis (http://jvenn.toulouse.inra.fr/app/example.html) for differentially expressed genes in KD versus NC group or OE versus NC group. ***P < 0.001

To further explore the downstream regulatory mechanisms of NR_002794, lentiviral particles that could overexpress NR_002794 (lenti-NR_002794) were packaged. Also, sh-NR_002794#1, sh-NR_002794#2, and sh-NR_002794#3 knockdown plasmids were constructed. Knockdown efficiency analysis revealed that the transfection of sh-NR_002794#1, sh-NR_002794#2, or sh-NR_002794#3 knockdown plasmid led to the notable reduction of NR_002794 level in SWAN71 cells (Figure 1a). The highest knockdown efficiency was observed in cells transfected with sh-NR_002794#2 plasmid. Thus, the sh-NR_002794#2 plasmid was transfected into HEK293T cells together with the packaging plasmid mix to obtain lentiviral particles that could silence NR_002794 (sh-NR_002794). Next, RNA-seq analysis was performed in SWAN71 cells infected with lenti-NR_002794 (OE group), sh-NR_002794 (KD group), or control lentiviruses (NC group) to identify genes that could be regulated by NR_002794. RNA-seq outcomes showed that 342 genes were up-regulated and 323 genes were down-regulated in the KD versus NC group (Supplementary Table 1). Also, 549 genes were identified to be differentially expressed (218 up-regulated, 331 down-regulated) in the OE versus NC group (Supplementary Table 2). To identify genes with similar expression patterns or similar functions, cluster analysis was performed for these differentially expressed genes in the KD versus NC group and OE versus NC group. As a result, a total of 10 gene clusters were identified (Figure 1b). In the following experiments, clusters 1 and 3 were selected for further investigations because genes in these clusters presented converse change trends in KD versus NC and OE versus NC groups. We believed that these genes had a high possibility to be regulated by NR_002794. Combined with the data in Supplementary Table 1 and Supplementary Table 2, 159 genes were found to be differentially expressed in both KD versus NC group and OE versus NC group (Figure 1c, Supplementary Table 3). KEGG enrichment analysis revealed that these 159 common dysregulated genes in both KD versus NC group and OE versus NC group mainly participated in the regulation of the pathways such as cytokine-cytokine receptor interaction, nucleotide excision repair, viral myocarditis, and Toll-like receptor/PI3K-AKT/MAPK/Wnt signaling pathways (Supplementary Table 4), suggesting that NR_002794 might be involved in the regulation of these pathways.

### Effects of NR_002794 overexpression or knockdown on AKT, ERK1/2 and caspase3 apoptotic pathways

**Figure 2.**
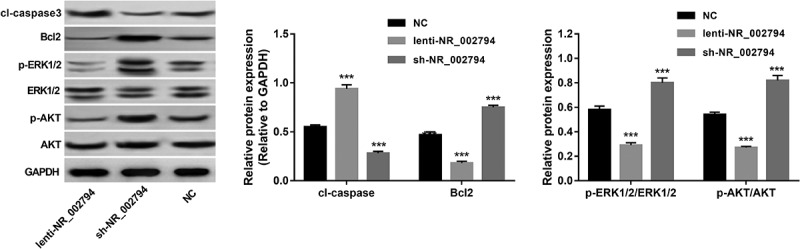
**Effects of NR_002794 overexpression or knockdown on AKT, ERK1/2, and apoptotic pathways**. SWAN71 cells were infected with lenti-NR_002794, sh-NR_002794, or control lentiviruses for 72 h. Next, protein levels of cleaved-caspase 3, Bcl2, p-ERK1/2, ERK1/2, p-AKT, and AKT were measured through western blot assay. ***P < 0.001

As mentioned above, KEGG enrichment analysis suggested that NR_002794 might be implicated in nucleotide excision repair and PI3K-AKT/MAPK pathways. Also, our prior study showed that NR_002794 overexpression could influence cell apoptosis in SWAN71 cells [18]. Thus, the effects of NR_002794 overexpression or knockdown on AKT, ERK1/2, and apoptotic pathways were further investigated by western blot assay in SWAN71 cells. Results showed that NR_002794 overexpression could trigger the marked increase of cleaved-caspase3 protein level and noticeable reduction of Bcl2 protein level and p-ERK/ERK and p-AKT/AKT ratios in SWAN71 cells (Figure 2). Conversely, reduced cleaved-caspase3 level, increased Bcl2 expression, and up-regulated p-ERK/ERK and p-AKT/AKT ratios were observed in NR_002794-depleted SWAN71 cells relative to the negative control group (Figure 2). In other words, these data suggested that NR_002794 could inactivate AKT and ERK1/2 pathways and activate caspase3 apoptotic signaling in SWAN71 cells.

### Effects of NR_002794 overexpression or knockdown on the expression of 9 potential targets: validation by RT-qPCR assay

**Figure 3.**
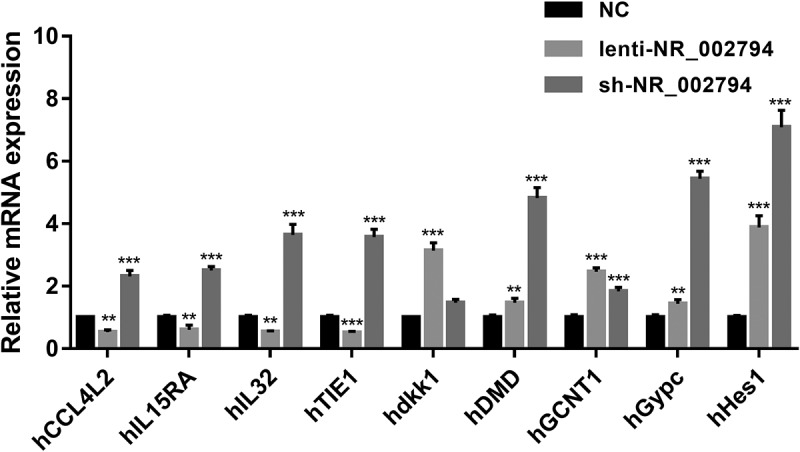
**Effects of NR_002794 overexpression or knockdown on the expression of CCL4L2/IL15RA/IL32/TIE1/dkk1/DMD/GCNT1/Gypc/Hes1**. SWAN71 cells were infected with lenti-NR_002794, sh-NR_002794, or control lentiviruses for 48 h. Next, the expression levels of CCL4L2/IL15RA/IL32/TIE1/dkk1/DMD/GCNT1/Gypc/Hes1 were measured by RT-qPCR assay. **P < 0.01. ***P < 0.001

Among genes in cluster 1, 14 genes were found to be notably up-regulated in the KD versus NC group and markedly down-regulated in the OE versus NC group (Supplementary Table 5). Among genes in cluster 3, 9 genes were identified to be noticeably down-regulated in the KD versus NC group and conspicuously up-regulated in the OE versus NC group (Supplementary Table 6). Among these 23 differentially expressed genes with the inverse alteration trends in KD versus NC and OE versus NC groups, C-C motif chemokine ligand 4 like 2 (CCL4L2)/interleukin 15 receptor subunit alpha (IL15RA)/interleukin 32 (IL32)/TIE1/dickkopf 1 (dkk1)/dystrophin (DMD)/glucosaminyl (N-acetyl) transferase 1 (GCNT1)/glycophorin C (Gerbich blood group) (Gypc)/hes family bHLH transcription factor 1 (Hes1) were picked out for further RT-qPCR experiment validation. The RT-qPCR assay demonstrated that enforced expression of NR_002794 could markedly reduce the expression levels of CCL4L2, IL15RA, IL32, and TIE1 in SWAN71 cells (Figure 3). Inversely, NR_002794 depletion led to a notable increase in the expression levels of CCL4L2, IL15RA, IL32, and TIE1 in SWAN71 cells (Figure 3). The influences of NR_002794 overexpression or knockdown on CCL4L2, IL15RA, IL32, and TIE1 expression were consistent in RT-qPCR and RNA-seq analyses. However, our data showed that NR_002794 overexpression or knockdown triggered the increase of dkk1, DMD, GCNT1, Gypc, and Hes1 expression levels, which was inconsistent with the RNA-seq outcomes (Figure 3).

### NR_002794 suppressed cell proliferation and migration by down-regulating TIE1 in SWAN71 cells

**Figure 4.**
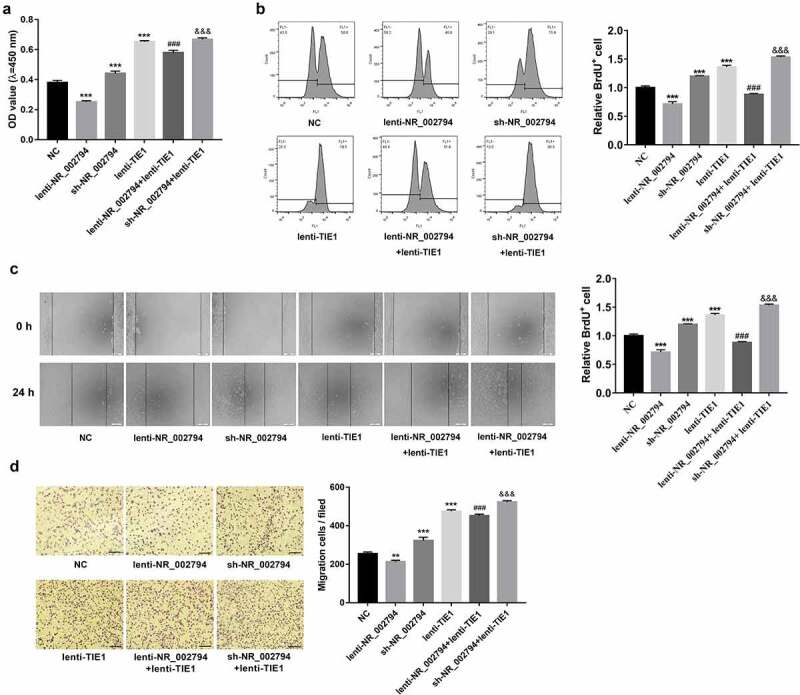
**NR_002794 suppressed cell proliferation and migration by silencing TIE1 in SWAN71 cells**. (a-d) SWAN71 cells were infected with lenti-NR_002794, sh-NR_002794, lenti-TIE1, lenti-NR_002794+ lenti-TIE1, sh-NR_002794+ lenti-TIE1 or control lentiviruses. (a and b) At 48 h post-infection, cell proliferative ability was measured by CCK-8 (a) and BrdU (b) assays. (c and d) Cell migratory ability was examined through wound healing assay (c) and transwell migration assay (d). *: treated group versus NC group. #: treated group versus lenti-NR_002794 group. &: treated group versus sh-NR_002794 group. ***P < 0.001. ###*P* < 0.001. &&&*P* < 0.001

Consistent with our prior study, CCK-8 and BrdU assays demonstrated that ectopic expression of NR_002794 weakened the proliferative potential of SWAN71 cells (Figure 4a and 4b). Conversely, NR_002794 knockdown facilitated SWAN71 cell proliferation (Figure 4a and 4b). In this text, we further demonstrated that TIE1 overexpression reversed NR_002794-mediated anti-proliferative effect, and enhanced the promotive effect of NR_002794 loss on cell proliferation in SWAN71 cells (Figure 4a and 4b). Additionally, enforced expression of TIE1 markedly potentiated the proliferative activity of SWAN71 cells (Figure 4a and 4b). Wound healing assays and transwell migration showed that NR_002794 overexpression hindered cell migration, while NR_002794 depletion triggered the notable increase of cell migratory capacity in SWAN71 cells (Figure 4c and 4d). Additionally, TIE1 up-regulation abrogated the detrimental effect of NR_002794 on cell migration and potentiated the stimulative effect of NR_002794 depletion on cell migration in SWAN71 cells (Figure 4c and 4d). Furthermore, enforced expression of TIE1 led to the noticeable elevation of cell migratory potential in SWAN71 cells (Figure 4c and 4d).

### NR_002794 facilitated cell apoptosis through down-regulating TIE1 in SWAN71 cells

**Figure 5.**
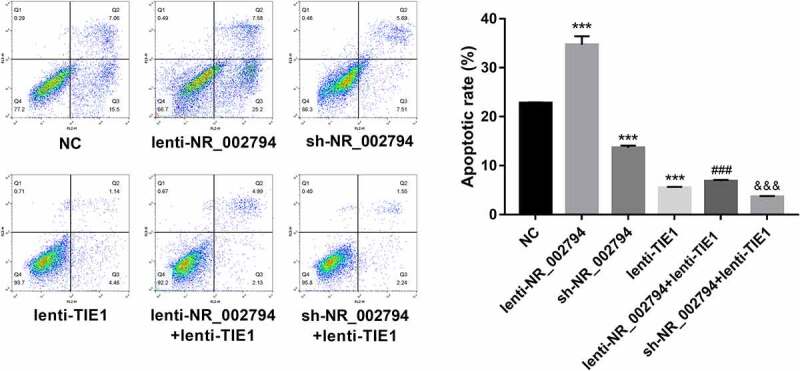
**NR_002794 facilitated cell apoptosis through down-regulating TIE1 in SWAN71 cells**. SWAN71 cells were infected with lenti-NR_002794, sh-NR_002794, lenti-TIE1, lenti-NR_002794+ lenti-TIE1, sh-NR_002794+ lenti-TIE1 or control lentiviruses. Cell apoptotic rate was measured through flow cytometry at 72 h post infection. *: treated group versus NC group. #: treated group versus lenti-NR_002794 group. &: treated group versus sh-NR_002794 group. ***P < 0.001. ###*P* < 0.001. &&&*P* < 0.001

Moreover, our data revealed that enforced expression of NR_002794 induced the notable increase of cell apoptotic rate, and TIE1 overexpression abolished NR_002794-mediated pro-apoptotic effect in SWAN71 cells (Figure 5). Conversely, NR_002794 knockdown inhibited cell apoptosis, while this effect was further enhanced by the TIE1 increase in SWAN71 cells (Figure 5). Additionally, TIE1 overexpression triggered the marked reduction of cell apoptotic rate in SWAN71 cells (Figure 5).

## Discussion

In this text, 159 potential downstream targets of NR_002794 were screened out by comparative transcriptomics analysis. KEGG enrichment analysis showed that these 159 genes were significantly enriched in multiple pathways including cytokine-cytokine receptor interaction, nucleotide excision repair, viral myocarditis, and Toll-like receptor/PI3K-AKT/MAPK/Wnt signaling pathways, suggesting the vital roles of NR_002794 in these pathways. AKT and ERK1/2 signaling pathways have been found to be involved in the regulation of PE and trophoblast development [30–32]. For instance, the inhibition of the PI3K/AKT pathway by LY294002 led to the notable reduction of cell proliferative and invasive abilities and a marked increase of cell apoptotic percentage in HTR-8/SVneo trophoblast cells [33]. The phosphorylation levels of PI3K and AKT were noticeably reduced in the placentas of PE mice compared to the control group [34]. The ERK1/2 phosphorylation levels and p-ERK/ERK ratio were noticeably reduced in the placental tissues of PE rats relative to the control group [35]. Cyclophilin A weakened the migratory and invasive abilities of trophoblast cells *in vitro* and *in vivo* and triggered PE-like features in pregnant mice by inactivating p38 MAPK and ERK1/2 signaling pathways [36]. Moreover, our previous study demonstrated that enforced expression of NR_002794 could induce cell apoptosis in SWAN71 cells. Thus, we further investigated the effects of NR_002794 overexpression or knockdown on AKT, ERK1/2, and apoptotic pathways in SWAN71 cells. Our data revealed that NR_002794 up-regulation could inactivate AKT and ERK1/2 signaling pathways and activate Bcl-2/caspase3 cell apoptotic signaling in SWAN71 cells.

Also, our data showed that 23 notably dysregulated genes presented the inverse change trends in KD versus NC and OE versus NC groups. We supposed that these genes had a high possibility to be regulated by NR_002794 in SWAN71 cells. Based on the annotation analyses for these 23 genes (Supplementary Table 7) and the above-mentioned KEGG enrichment analysis, we selected CCL4L2/IL15RA/IL32 (three genes related to cytokine-cytokine receptor interaction)/TIE1 (an angiogenesis-related gene), DKK1 (a gene involved in WNT1 signaling)/DMD (a gene implicated in viral myocarditis)/GCNT1 (a gene related to metabolic pathways and cardiovascular disease)/Gypc (a cardiovascular disease-related gene)/Hes1 (an unannotated gene) for further investigations. Moreover, DKK1 has been found to be abnormally expressed in preeclamptic placentas and to be related to the pathogenesis of PE [37–39]. In this project, both RNA-seq and RT-qPCR outcomes revealed that NR_002794 overexpression triggered the notable reduction in the expression levels of CCL4L2, IL15RA, IL32, and TIE1, while NR_002794 knockdown markedly promoted the expression of these genes in SWAN71 cells. However, RT-qPCR analyses showed that the expression levels of dkk1, DMD, GCNT1, Gypc, and Hes1 were increased in both KD versus NC and OE versus NC groups, which was not in line with the RNA-seq results.

Currently, a large amount of evidence suggests that the abnormality of angiogenesis is closely associated with the development of PE [2,40,41]. TIE1, also named TIE, has emerged as a vital regulator in angiogenesis recently [42,43]. Moreover, a stronger TIE1 immunoreactivity was observed in angiogenic cells and trophoblast cells of human placental tissues [44,45]. Additionally, TIE1 serum levels were notably reduced in PE women than in non-pregnant women and healthy pregnant women [46]. Given the close association of TIE1 and angiogenesis/PE development, we further explored whether NR_002794 could exert its functions by regulating TIE1 in SWAN71 cells. Our data revealed that NR_002794 weakened cell proliferative and migratory capacities and induced cell apoptosis through down-regulating TIE1 in SWAN71 cells. Moreover, TIE1 overexpression facilitated cell proliferation and migration and hindered cell apoptosis in SWAN71 cells.

## Conclusion

In summary, our outcomes showed that lncRNA NR_002794 exerted its anti-proliferative, anti-migratory, and pro-apoptotic effects through regulating AKT and ERK1/2 pathways and TIE1 expression in human trophoblast cells. Additionally, multiple potential targets or downstream signaling pathways of NR_002794 were identified in our project, which might contribute to a better understanding of the pathogenic mechanisms of NR_002794 in PE. Furthermore, our study provides an in-depth insight into the molecular basis implicated in trophoblast and PE development. However, our experimental design is rough and it is imperative to further examine the effects of the NR_002794/TIE1 axis on PE development *in vivo*.

## Supplementary Material

Supplemental Material

## Highlights

1. Some potential targets and downstream pathways of NR_002794 were identified.

2. NR_002794 inactivated AKT and ERK1/2 pathways in SWAN71 trophoblast cells.

3. NR_002794 inhibited TIE1 expression in SWAN71 trophoblast cells.

4. NR_002794 hampered cell proliferation and migration and induced cell apoptosis.

5. NR_002794 exerted its functions by down-regulating TIE1 in SWAN71 trophoblast cells.

## References

[1] Umesawa M, Kobashi G. Epidemiology of hypertensive disorders in pregnancy: prevalence, risk factors, predictors and prognosis. Hypertens Res. 2017;40(3):213–220. 27682655 10.1038/hr.2016.126

[2] Nirupama R, Divyashree S, Janhavi P, et al. Preeclampsia: pathophysiology and management. J Gynecol Obstet Hum Reprod. 2021;50(2):101975. 33171282 10.1016/j.jogoh.2020.101975

[3] Ives CW, Sinkey R, Rajapreyar I, et al. Preeclampsia-pathophysiology and clinical presentations: JACC state-of-the-art review. J Am Coll Cardiol. 2020;76(14):1690–1702. 33004135 10.1016/j.jacc.2020.08.014

[4] Rana S, Lemoine E, Granger JP, et al. Preeclampsia: pathophysiology, challenges, and perspectives. Circ Res. 2019;124(7):1094–1112. 30920918 10.1161/CIRCRESAHA.118.313276

[5] Jim B, Karumanchi SA. Preeclampsia: pathogenesis, prevention, and long-term complications. Semin Nephrol 2017;37(4):386–397. 10.1016/j.semnephrol.2017.05.011 28711078

[6] Lunghi L, Ferretti ME, Medici S, et al. Control of human trophoblast function. ReprodBiol Endocrinol. 2007;5:6. 10.1186/1477-7827-5-6 PMC1800852 17288592

[7] Dhariwal NK, Lynde GC. Update in the management of patients with Preeclampsia. Anesthesiol Clin. 2017;35(1):95–106. 28131123 10.1016/j.anclin.2016.09.009

[8] Huppertz B. The critical role of abnormal trophoblast development in the etiology of preeclampsia. Curr Pharm Biotechnol. 2018;19(10):771–780. 29701150 10.2174/1389201019666180427110547 PMC6463401

[9] Ridder A, Giorgione V, Khalil A. Preeclampsia: the relationship between uterine artery blood flow and trophoblast function. Int J Mol Sci. 2019;20(13):3263. 10.3390/ijms20133263 PMC6651116 31269775

[10] Azizi Z, Mirtavoos-Mahyari H, Karimi R, et al. Long non-coding RNAs: diverse roles in various disorders. Hum Antibodies. 2019;27(4):221–225. 30909207 10.3233/HAB-190374

[11] Tsagakis I, Douka K, Birds I. Long non-coding RNAs in development and disease: conservation to mechanisms. J Pathol. 2020;250(5):480–495. 32100288 10.1002/path.5405 PMC8638664

[12] Statello L, Guo CJ, Chen LL. Gene regulation by long non-coding RNAs and its biological functions. Nat Rev Mol Cell Biol. 2021;22(2):96–118. 33353982 10.1038/s41580-020-00315-9 PMC7754182

[13] Peng WX, Koirala P, Mo YY. LncRNA-mediated regulation of cell signaling in cancer. Oncogene. 2017;36(41):5661–5667. 28604750 10.1038/onc.2017.184 PMC6450570

[14] Moradi MT, Rahimi Z, Vaisi-Raygani A. New insight into the role of long non-coding RNAs in the pathogenesis of preeclampsia. Hypertens Pregnancy. 2019;38(1):41–51. 30707634 10.1080/10641955.2019.1573252

[15] Yang X, Meng T. Long noncoding RNA in preeclampsia: transcriptional noise or innovative indicators? Biomed Res Int. 2019;2019:5437621. 31111058 10.1155/2019/5437621 PMC6487157

[16] Tang D, Geng L, Ma J. lncRNA PROX1-AS1 mediates the migration and invasion of placental trophoblast cells via the miR-211-5p/caspase-9 axis. Bioengineered. 2021;12(1):4100–4110. 34288800 10.1080/21655979.2021.1953213 PMC8806442

[17] Chen Q, Jiang S, Liu H, *et al*. Association of lncRNA SH3PXD2A-AS1 with preeclampsia and its function in invasion and migration of placental trophoblast cells. Cell Death Dis. 2020;11(7):583. 32719429 10.1038/s41419-020-02796-0 PMC7385659

[18] Ma Y, Liang X, Wu H, et al. Long non‑coding RNA NR_002794 is upregulated in pre‑eclampsia and regulates the proliferation, apoptosis and invasion of trophoblast cells. Mol Med Rep. 2019;20(5):4567–4575. 31702023 10.3892/mmr.2019.10701 PMC6797946

[19] Mao M, Zheng X, Jin B, et al. Effects of CD44 and E-cadherin overexpression on the proliferation, adhesion and invasion of ovarian cancer cells. Exp Ther Med. 2017;14(6):5557–5563. 29285092 10.3892/etm.2017.5259 PMC5740773

[20] Li G, Yang J, Chong T, et al. TUG1 knockdown inhibits the tumorigenesis and progression of prostate cancer by regulating microRNA-496/Wnt/β-catenin pathway. Anticancer Drugs. 2020;31(6):592–600. 32427740 10.1097/CAD.0000000000000882

[21] Falzone L, Salemi R, Travali S, et al. overexpression is associated with intragenic hypermethylation of MMP9 gene in melanoma. Aging (Albany NY). 2016;8(5):933–944. 27115178 10.18632/aging.100951 PMC4931845

[22] Hou W, Ye C, Chen M, et al. Bergenin activates SIRT1 as a novel therapeutic agent for osteogenesis of bone mesenchymal stem cells. Front Pharmacol. 2019;10:618. 31258473 10.3389/fphar.2019.00618 PMC6586741

[23] Xu Z, Orkwis JA, DeVine BM, et al. Extracellular matrix cues modulate Schwann cell morphology, proliferation, and protein expression. J Tissue Eng Regen Med. 2020;14(2):229–242. 31702874 10.1002/term.2987

[24] Frión-Herrera Y, Gabbia D, Scaffidi M, et al. The cuban propolis component nemorosone inhibits proliferation and metastatic properties of human colorectal cancer cells. Int J Mol Sci. 2020;21(5):1827. 10.3390/ijms21051827 PMC7084755 32155848

[25] Li J, Hu L, Zhou T, et al. Taxifolin inhibits breast cancer cells proliferation, migration and invasion by promoting mesenchymal to epithelial transition viaβ-catenin signaling. Life Sci. 2019;232:116617. 31260685 10.1016/j.lfs.2019.116617

[26] Zhang L, Li H, Li M, et al. LRP6 is involved in the proliferation, migration and invasion of trophoblast cells via miR-346. Int J Mol Med. 2020;46(1):211–223. 32319541 10.3892/ijmm.2020.4570 PMC7255486

[27] Fangjun L, Zhijia Y. Tumor suppressive roles of eugenol in human lung cancer cells. Thorac Cancer. 2018;9(1):25–29. 29024500 10.1111/1759-7714.12508 PMC5754308

[28] Gao X, Zhang X, Hu J, et al. Aconitine induces apoptosis in H9c2 cardiac cells via mitochondria-mediated pathway. Mol Med Rep. 2018;17(1):284–292. 29115599 10.3892/mmr.2017.7894 PMC5780139

[29] Liu Y, Shao E, Zhang Z, et al. Derivative induces apoptosis through the mitochondria p53 pathway in HepG2 cells. Front Pharmacol. 2019;10:762. 31354481 10.3389/fphar.2019.00762 PMC6635656

[30] Liu C, Wang H, Yang M, et al. Downregulation of cAMP-dependent protein kinase inhibitor-b promotes preeclampsia by decreasing phosphorylated AKT. Reprod Sci. 2021;28(1):178–185. 32676926 10.1007/s43032-020-00258-8 PMC7782383

[31] You X, Cui H, Yu N, et al. Knockdown of DDX46 inhibits trophoblast cell proliferation and migration through the PI3K/Akt/mTOR signaling pathway in preeclampsia. Open life sci. 2020;15(1):400–408. 10.1515/biol-2020-0043 PMC7874595 33817228

[32] Li M, Cheng W, Luo J, et al. Loss of selenocysteine insertion sequence binding protein 2 suppresses the proliferation, migration/invasion and hormone secretion of human trophoblast cells via the PI3K/Akt and ERK signaling pathway. Placenta. 2017;55:81–89. 28623977 10.1016/j.placenta.2017.05.007

[33] Xu Y, Sui L, Qiu B, et al. ANXA4 promotes trophoblast invasion via the PI3K/Akt/eNOS pathway in preeclampsia. Am J Physiol Cell Physiol. 2019;316(4):C481–c491. 30673304 10.1152/ajpcell.00404.2018

[34] Huang J, Zheng L, Wang F, et al. Mangiferin ameliorates placental oxidative stress and activates PI3K/Akt/mTOR pathway in mouse model of preeclampsia. Arch Pharm Res. 2020;43(2):233–241. 31989480 10.1007/s12272-020-01220-7

[35] Wang Y, Jie L, Gong H, et al. miR-30 inhibits proliferation of trophoblasts in preeclampsia rats partially related to MAPK/ERK pathway. Exp Ther Med. 2020;20(2):1379–1384. 32742372 10.3892/etm.2020.8866 PMC7388335

[36] Hu H, Jiang J, Chen Q, *et al*. Cyclophilin A inhibits trophoblast migration and invasion in vitro and vivo through p38/ERK/JNK pathways and causes features of preeclampsia in mice. Life Sci. 2020;261:118351. 32858039 10.1016/j.lfs.2020.118351

[37] Li N, Huang L, Li Y, et al. Lin28B/miR-92b promote the proliferation, migration, and invasion in the pathogenesis of preeclampsia via the DKK1/Wnt/β-Catenin pathway. Reprod Sci. 2020;27(3):815–822. 32072603 10.1007/s43032-019-00083-8

[38] Zhang Z, Li H, Zhang L, et al. Differential expression of β-catenin and Dickkopf-1 in the third trimester placentas from normal and preeclamptic pregnancies: a comparative study. ReprodBiol Endocrinol. 2013;11:17. 10.1186/1477-7827-11-17 PMC3599361 23452984

[39] Wang X, Zhang Z, Zeng X. Wnt/β-catenin signaling pathway in severe preeclampsia. J Mol Histol. 2018;49(3):317–327. 29603045 10.1007/s10735-018-9770-7

[40] Schrey-Petersen S, Stepan H. Anti-angiogenesis and preeclampsia in 2016. Curr Hypertens Rep. 2017;19(1):6. 28155021 10.1007/s11906-017-0706-5

[41] Shi M, Chen X, Li H, et al. δ-tocotrienol suppresses the migration and angiogenesis of trophoblasts in preeclampsia and promotes their apoptosis via miR-429/ZEB1 axis. Bioengineered. 2021;12(1):1861–1873. 34002673 10.1080/21655979.2021.1923238 PMC8806315

[42] Mueller SB, Kontos CD. Tie1: an orphan receptor provides context for angiopoietin-2/Tie2 signaling. J Clin Investig. 2016;126(9):3188–3191. 27548526 10.1172/JCI89963 PMC5004958

[43] Yang P, Chen N, Jia JH, et al. Tie-1: a potential target for anti-angiogenesis therapy. J Huazhong Univ Sci Tech Med sci. 2015;35(5):615–622. 10.1007/s11596-015-1479-1 26489611

[44] Seval Y, Sati L, Celik-Ozenci C, et al. The distribution of angiopoietin-1, angiopoietin-2 and their receptors tie-1 and tie-2 in the very early human placenta. Placenta. 2008;29(9):809–815. 18675456 10.1016/j.placenta.2008.06.009

[45] Kayisli UA, Cayli S, Seval Y, et al. Spatial and temporal distribution of Tie-1 and Tie-2 during very early development of the human placenta. Placenta. 2006;27(6–7):648–659. 16026828 10.1016/j.placenta.2005.05.013

[46] Vuorela P, Matikainen MT, Kuusela P, et al. Endothelial tie receptor antigen in maternal and cord blood of healthy and preeclamptic subjects. Obstet Gynecol. 1998;92(2):179–183. 9699747 10.1016/s0029-7844(98)00195-1

