# Supplementary material for: Identification of downstream targets and signaling pathways of long non-coding RNA NR_002794 in human trophoblast cells
Source: Bioengineered. 2021 Sep 13;12(1):6617–28. doi: 10.1080/21655979.2021.1974808 (PMC8806843; doi:10.1080/21655979.2021.1974808)
Supplement: Supplemental Material [file KBIE_A_1974808_SM0255.zip › supplementary/supplementary Table 1.docx]

**The sequences of 3 pairs of oligos targeting NR_002794**

| **name** | **sequences** |
| --- | --- |
| sh-NR_002794#1-sense (5'-3') | CACCGGTGGTCAACATCACCATGGTCGAAACCATGGTGATGTTGACCACC |
| sh-NR_002794#1-antisense (5'-3') | AAAAGGTGGTCAACATCACCATGGTTTCGACCATGGTGATGTTGACCACC |
| sh-NR_002794#2-sense (5'-3') | CACCGTGCTTCTGTAAGGGCTACAACGAATTGTAGCCCTTACAGAAGCAC |
| sh-NR_002794#2-antisense (5'-3') | AAAAGTGCTTCTGTAAGGGCTACAATTCGTTGTAGCCCTTACAGAAGCAC |
| sh-NR_002794#3-sense (5'-3') | CACCGGCAAGGTTTGGTGCAAAATCCGAAGATTTTGCACCAAACCTTGCC |
| sh-NR_002794#3-antisense (5'-3') | AAAAGGCAAGGTTTGGTGCAAAATCTTCGGATTTTGCACCAAACCTTGCC |

**Overexpression primer sequences**

| **name** | **forward primer (5'-3')** | **reverse primer (5'-3')** |
| --- | --- | --- |
| TIE1 | AATTAAGGAAGCTAGCatggtctggcgggtgc | TAAATCCAAGGCGCGCCtcaggcctcctcagctgtggc |

| **QPCR primer sequences** |  |  |
| --- | --- | --- |
| **gene name** | **foward(5'-3')** | **revese(5'-3')** |
| NR_002794 | TCTGTCTGTGCAGTGCTTCTG | GTCGTCCTGCAGCAAGTAGC |
| CCL4L2 | CCGCCTGCTGCTTTTCTTAC | TTGCTTGCCTACCACAGC |
| IL15RA | CCCAGCTCAAACAACACAGC | AGGTAGCATGCCAGGAGAGA |
| IL32 | AGAGGGCTACCTGGAGACAG | CACCACCTTCTCCTTCACCC |
| TIE1 | AGAGCATGGGACAGCCTCTA | CCAGGTCCCTGTGGATGAAC |
| Dkk1 | GCCTCAGGATTGTGTTGTGC | ATCCGGCAAGACAGACCTTC |
| DMD | TCTACAGAGGTCCGACAGCA | CTCATTGGCTTTCCAGGGGT |
| GCNT1 | TATCTCTGGGCCACCATCCA | GTTCAAGTCACCAGCTCCGA |
| Gypc | AGCCTGATCCAGGGATGTCT | TGCATCTGCACTCTCAGCAA |
| Hes1 | GTGTCAACACGACACCGGAT | GGAATGCCGCGAGCTATCTT |
