# Supplementary material for: Identification of downstream targets and signaling pathways of long non-coding RNA NR_002794 in human trophoblast cells
Source: Bioengineered. 2021 Sep 13;12(1):6617–28. doi: 10.1080/21655979.2021.1974808 (PMC8806843; doi:10.1080/21655979.2021.1974808)
Supplement: Supplemental Material [file KBIE_A_1974808_SM0255.zip › supplementary/supplementary Table 2.docx]

| **Differentially expressed genes in OE versus NC group** | | | | | |  |
| --- | --- | --- | --- | --- | --- | --- |
| Gene id | GeneName | GeneDescription | log2FoldChange | pValue | qValue | result |
| ENSG00000210082 | MT-RNR2 | mitochondrially encoded 16S RNA [Source:HGNC Symbol;Acc:HGNC:7471] | -1.25794 | 0 | 0 | down |
| ENSG00000120738 | EGR1 | early growth response 1 [Source:HGNC Symbol;Acc:HGNC:3238] | -1.91396 | 0 | 0 | down |
| ENSG00000198899 | MT-ATP6 | mitochondrially encoded ATP synthase 6 [Source:HGNC Symbol;Acc:HGNC:7414] | -1.03753 | 0 | 0 | down |
| ENSG00000255508 | AP002990.1 | - | -3.69772 | 0 | 0 | down |
| ENSG00000108518 | PFN1 | profilin 1 [Source:HGNC Symbol;Acc:HGNC:8881] | -1.22607 | 0 | 0 | down |
| ENSG00000173402 | DAG1 | dystroglycan 1 [Source:HGNC Symbol;Acc:HGNC:2666] | -3.04702 | 0 | 0 | down |
| ENSG00000152291 | TGOLN2 | trans-golgi network protein 2 [Source:HGNC Symbol;Acc:HGNC:15450] | -2.47461 | 0 | 0 | down |
| ENSG00000175592 | FOSL1 | FOS like 1, AP-1 transcription factor subunit [Source:HGNC Symbol;Acc:HGNC:13718] | -1.20105 | 0 | 0 | down |
| ENSG00000234745 | HLA-B | major histocompatibility complex, class I, B [Source:HGNC Symbol;Acc:HGNC:4932] | -1.21108 | 0 | 0 | down |
| ENSG00000163359 | COL6A3 | collagen type VI alpha 3 chain [Source:HGNC Symbol;Acc:HGNC:2213] | -2.62757 | 0 | 0 | down |
| ENSG00000185022 | MAFF | MAF bZIP transcription factor F [Source:HGNC Symbol;Acc:HGNC:6780] | -3.0145 | 0 | 0 | down |
| ENSG00000172466 | ZNF24 | zinc finger protein 24 [Source:HGNC Symbol;Acc:HGNC:13032] | -2.61262 | 0 | 0 | down |
| ENSG00000167244 | IGF2 | insulin like growth factor 2 [Source:HGNC Symbol;Acc:HGNC:5466] | -3.53848 | 0 | 0 | down |
| ENSG00000276085 | CCL3L1 | C-C motif chemokine ligand 3 like 1 [Source:HGNC Symbol;Acc:HGNC:10628] | -2.38018 | 0 | 0 | down |
| ENSG00000150991 | UBC | ubiquitin C [Source:HGNC Symbol;Acc:HGNC:12468] | -1.13125 | 0 | 0 | down |
| ENSG00000105825 | TFPI2 | tissue factor pathway inhibitor 2 [Source:HGNC Symbol;Acc:HGNC:11761] | -1.31471 | 0 | 0 | down |
| ENSG00000137628 | DDX60 | DExD/H-box helicase 60 [Source:HGNC Symbol;Acc:HGNC:25942] | -1.27182 | 0 | 0 | down |
| ENSG00000179222 | MAGED1 | MAGE family member D1 [Source:HGNC Symbol;Acc:HGNC:6813] | -1.47696 | 0 | 0 | down |
| ENSG00000196611 | MMP1 | matrix metallopeptidase 1 [Source:HGNC Symbol;Acc:HGNC:7155] | -1.79582 | 0 | 0 | down |
| ENSG00000169429 | CXCL8 | C-X-C motif chemokine ligand 8 [Source:HGNC Symbol;Acc:HGNC:6025] | -1.18144 | 0 | 0 | down |
| ENSG00000108468 | CBX1 | chromobox 1 [Source:HGNC Symbol;Acc:HGNC:1551] | -1.73991 | 0 | 0 | down |
| ENSG00000213782 | DDX47 | DEAD-box helicase 47 [Source:HGNC Symbol;Acc:HGNC:18682] | -2.67415 | 0 | 0 | down |
| ENSG00000135052 | GOLM1 | golgi membrane protein 1 [Source:HGNC Symbol;Acc:HGNC:15451] | -1.99403 | 0 | 0 | down |
| ENSG00000197965 | MPZL1 | myelin protein zero like 1 [Source:HGNC Symbol;Acc:HGNC:7226] | -1.8836 | 0 | 0 | down |
| ENSG00000136830 | FAM129B | family with sequence similarity 129 member B [Source:HGNC Symbol;Acc:HGNC:25282] | -1.30474 | 0 | 0 | down |
| ENSG00000120129 | DUSP1 | dual specificity phosphatase 1 [Source:HGNC Symbol;Acc:HGNC:3064] | -1.12485 | 0 | 0 | down |
| ENSG00000161011 | SQSTM1 | sequestosome 1 [Source:HGNC Symbol;Acc:HGNC:11280] | -1.16802 | 0 | 0 | down |
| ENSG00000143549 | TPM3 | tropomyosin 3 [Source:HGNC Symbol;Acc:HGNC:12012] | -1.37499 | 0 | 0 | down |
| ENSG00000164362 | TERT | telomerase reverse transcriptase [Source:HGNC Symbol;Acc:HGNC:11730] | -2.4556 | 0 | 0 | down |
| ENSG00000116489 | CAPZA1 | capping actin protein of muscle Z-line alpha subunit 1 [Source:HGNC Symbol;Acc:HGNC:1488] | -1.06158 | 0 | 0 | down |
| ENSG00000161847 | RAVER1 | ribonucleoprotein, PTB binding 1 [Source:HGNC Symbol;Acc:HGNC:30296] | -1.54433 | 0 | 0 | down |
| ENSG00000132912 | DCTN4 | dynactin subunit 4 [Source:HGNC Symbol;Acc:HGNC:15518] | -1.12434 | 0 | 0 | down |
| ENSG00000171223 | JUNB | JunB proto-oncogene, AP-1 transcription factor subunit [Source:HGNC Symbol;Acc:HGNC:6205] | -1.43171 | 0 | 0 | down |
| ENSG00000234127 | TRIM26 | tripartite motif containing 26 [Source:HGNC Symbol;Acc:HGNC:12962] | -1.85582 | 0 | 0 | down |
| ENSG00000239264 | TXNDC5 | thioredoxin domain containing 5 [Source:HGNC Symbol;Acc:HGNC:21073] | -1.45879 | 0 | 0 | down |
| ENSG00000123689 | G0S2 | G0/G1 switch 2 [Source:HGNC Symbol;Acc:HGNC:30229] | -2.31263 | 0 | 0 | down |
| ENSG00000136826 | KLF4 | Kruppel like factor 4 [Source:HGNC Symbol;Acc:HGNC:6348] | -1.97116 | 0 | 0 | down |
| ENSG00000164284 | GRPEL2 | GrpE like 2, mitochondrial [Source:HGNC Symbol;Acc:HGNC:21060] | -1.94089 | 0 | 0 | down |
| ENSG00000176624 | MEX3C | mex-3 RNA binding family member C [Source:HGNC Symbol;Acc:HGNC:28040] | -2.69482 | 0 | 0 | down |
| ENSG00000120896 | SORBS3 | sorbin and SH3 domain containing 3 [Source:HGNC Symbol;Acc:HGNC:30907] | -1.31732 | 0 | 0 | down |
| ENSG00000063322 | MED29 | mediator complex subunit 29 [Source:HGNC Symbol;Acc:HGNC:23074] | -2.70265 | 0 | 0 | down |
| ENSG00000154710 | RABGEF1 | RAB guanine nucleotide exchange factor 1 [Source:HGNC Symbol;Acc:HGNC:17676] | -1.53008 | 6.40E-290 | 1.09E-287 | down |
| ENSG00000119729 | RHOQ | ras homolog family member Q [Source:HGNC Symbol;Acc:HGNC:17736] | -1.15625 | 7.93E-280 | 1.32E-277 | down |
| ENSG00000025800 | KPNA6 | karyopherin subunit alpha 6 [Source:HGNC Symbol;Acc:HGNC:6399] | -1.02113 | 8.13E-252 | 1.27E-249 | down |
| ENSG00000123094 | RASSF8 | Ras association domain family member 8 [Source:HGNC Symbol;Acc:HGNC:13232] | -1.32137 | 1.01E-250 | 1.56E-248 | down |
| ENSG00000172936 | MYD88 | myeloid differentiation primary response 88 [Source:HGNC Symbol;Acc:HGNC:7562] | -2.13742 | 1.14E-249 | 1.73E-247 | down |
| ENSG00000144802 | NFKBIZ | NFKB inhibitor zeta [Source:HGNC Symbol;Acc:HGNC:29805] | -1.34915 | 7.07E-239 | 1.03E-236 | down |
| ENSG00000277632 | CCL3 | C-C motif chemokine ligand 3 [Source:HGNC Symbol;Acc:HGNC:10627] | -2.54048 | 1.04E-237 | 1.51E-235 | down |
| ENSG00000130311 | DDA1 | DET1 and DDB1 associated 1 [Source:HGNC Symbol;Acc:HGNC:28360] | -1.37672 | 3.49E-232 | 4.88E-230 | down |
| ENSG00000144677 | CTDSPL | CTD small phosphatase like [Source:HGNC Symbol;Acc:HGNC:16890] | -1.22794 | 2.76E-228 | 3.81E-226 | down |
| ENSG00000135631 | RAB11FIP5 | RAB11 family interacting protein 5 [Source:HGNC Symbol;Acc:HGNC:24845] | -1.04502 | 5.10E-226 | 6.99E-224 | down |
| ENSG00000198860 | TSEN15 | tRNA splicing endonuclease subunit 15 [Source:HGNC Symbol;Acc:HGNC:16791] | -1.63553 | 2.18E-222 | 2.96E-220 | down |
| ENSG00000142784 | WDTC1 | WD and tetratricopeptide repeats 1 [Source:HGNC Symbol;Acc:HGNC:29175] | -1.18888 | 1.03E-214 | 1.36E-212 | down |
| ENSG00000204356 | NELFE | negative elongation factor complex member E [Source:HGNC Symbol;Acc:HGNC:13974] | -1.39655 | 4.80E-204 | 6.17E-202 | down |
| ENSG00000173517 | PEAK1 | pseudopodium enriched atypical kinase 1 [Source:HGNC Symbol;Acc:HGNC:29431] | -1.26899 | 1.10E-203 | 1.40E-201 | down |
| ENSG00000261915 | AC026954.2 | - | -7.24762 | 2.86E-203 | 3.64E-201 | down |
| ENSG00000106261 | ZKSCAN1 | zinc finger with KRAB and SCAN domains 1 [Source:HGNC Symbol;Acc:HGNC:13101] | -1.17003 | 3.76E-200 | 4.69E-198 | down |
| ENSG00000162892 | IL24 | interleukin 24 [Source:HGNC Symbol;Acc:HGNC:11346] | -1.35843 | 3.56E-196 | 4.33E-194 | down |
| ENSG00000264772 | AC016876.2 | - | -1.31828 | 1.75E-194 | 2.09E-192 | down |
| ENSG00000156671 | SAMD8 | sterile alpha motif domain containing 8 [Source:HGNC Symbol;Acc:HGNC:26320] | -1.16335 | 1.79E-188 | 2.07E-186 | down |
| ENSG00000126709 | IFI6 | interferon alpha inducible protein 6 [Source:HGNC Symbol;Acc:HGNC:4054] | -1.99902 | 6.75E-187 | 7.74E-185 | down |
| ENSG00000196878 | LAMB3 | laminin subunit beta 3 [Source:HGNC Symbol;Acc:HGNC:6490] | -1.09962 | 3.96E-182 | 4.46E-180 | down |
| ENSG00000162433 | AK4 | adenylate kinase 4 [Source:HGNC Symbol;Acc:HGNC:363] | -1.66453 | 1.32E-181 | 1.48E-179 | down |
| ENSG00000211460 | TSN | translin [Source:HGNC Symbol;Acc:HGNC:12379] | -1.20251 | 1.27E-171 | 1.36E-169 | down |
| ENSG00000160446 | ZDHHC12 | zinc finger DHHC-type containing 12 [Source:HGNC Symbol;Acc:HGNC:19159] | -2.35905 | 3.86E-170 | 4.12E-168 | down |
| ENSG00000173875 | ZNF791 | zinc finger protein 791 [Source:HGNC Symbol;Acc:HGNC:26895] | -2.09959 | 1.69E-167 | 1.76E-165 | down |
| ENSG00000047932 | GOPC | golgi associated PDZ and coiled-coil motif containing [Source:HGNC Symbol;Acc:HGNC:17643] | -1.62433 | 2.46E-166 | 2.52E-164 | down |
| ENSG00000185414 | MRPL30 | mitochondrial ribosomal protein L30 [Source:HGNC Symbol;Acc:HGNC:14036] | -1.3436 | 6.30E-164 | 6.38E-162 | down |
| ENSG00000095139 | ARCN1 | archain 1 [Source:HGNC Symbol;Acc:HGNC:649] | -1.10615 | 2.89E-163 | 2.92E-161 | down |
| ENSG00000110047 | EHD1 | EH domain containing 1 [Source:HGNC Symbol;Acc:HGNC:3242] | -1.486 | 1.55E-157 | 1.49E-155 | down |
| ENSG00000132535 | DLG4 | discs large MAGUK scaffold protein 4 [Source:HGNC Symbol;Acc:HGNC:2903] | -1.08217 | 2.71E-155 | 2.56E-153 | down |
| ENSG00000114251 | WNT5A | Wnt family member 5A [Source:HGNC Symbol;Acc:HGNC:12784] | -1.52605 | 2.67E-152 | 2.48E-150 | down |
| ENSG00000198040 | ZNF84 | zinc finger protein 84 [Source:HGNC Symbol;Acc:HGNC:13159] | -1.2159 | 3.96E-149 | 3.65E-147 | down |
| ENSG00000136167 | LCP1 | lymphocyte cytosolic protein 1 [Source:HGNC Symbol;Acc:HGNC:6528] | -1.35932 | 3.15E-146 | 2.85E-144 | down |
| ENSG00000197943 | PLCG2 | phospholipase C gamma 2 [Source:HGNC Symbol;Acc:HGNC:9066] | -1.1774 | 2.12E-141 | 1.87E-139 | down |
| ENSG00000007545 | CRAMP1 | cramped chromatin regulator homolog 1 [Source:HGNC Symbol;Acc:HGNC:14122] | -1.00224 | 2.77E-141 | 2.44E-139 | down |
| ENSG00000205213 | LGR4 | leucine rich repeat containing G protein-coupled receptor 4 [Source:HGNC Symbol;Acc:HGNC:13299] | -1.11718 | 2.68E-140 | 2.31E-138 | down |
| ENSG00000160888 | IER2 | immediate early response 2 [Source:HGNC Symbol;Acc:HGNC:28871] | -1.25663 | 2.13E-139 | 1.82E-137 | down |
| ENSG00000240184 | PCDHGC3 | protocadherin gamma subfamily C, 3 [Source:HGNC Symbol;Acc:HGNC:8716] | -1.27787 | 4.08E-137 | 3.40E-135 | down |
| ENSG00000124208 | TMEM189-UBE2V1 | TMEM189-UBE2V1 readthrough [Source:HGNC Symbol;Acc:HGNC:33521] | -2.54512 | 1.48E-133 | 1.19E-131 | down |
| ENSG00000175602 | CCDC85B | coiled-coil domain containing 85B [Source:HGNC Symbol;Acc:HGNC:24926] | -1.08045 | 3.82E-128 | 2.95E-126 | down |
| ENSG00000111348 | ARHGDIB | Rho GDP dissociation inhibitor beta [Source:HGNC Symbol;Acc:HGNC:679] | -2.94271 | 3.58E-125 | 2.74E-123 | down |
| ENSG00000122861 | PLAU | plasminogen activator, urokinase [Source:HGNC Symbol;Acc:HGNC:9052] | -1.086 | 1.00E-122 | 7.57E-121 | down |
| ENSG00000187134 | AKR1C1 | aldo-keto reductase family 1 member C1 [Source:HGNC Symbol;Acc:HGNC:384] | -1.04426 | 2.10E-122 | 1.58E-120 | down |
| ENSG00000085365 | SCAMP1 | secretory carrier membrane protein 1 [Source:HGNC Symbol;Acc:HGNC:10563] | -1.01976 | 2.42E-122 | 1.81E-120 | down |
| ENSG00000196182 | STK40 | serine/threonine kinase 40 [Source:HGNC Symbol;Acc:HGNC:21373] | -1.46257 | 3.21E-121 | 2.39E-119 | down |
| ENSG00000167323 | STIM1 | stromal interaction molecule 1 [Source:HGNC Symbol;Acc:HGNC:11386] | -1.40155 | 3.34E-118 | 2.42E-116 | down |
| ENSG00000284431 | AL022238.4 | - | -2.3693 | 1.13E-113 | 7.89E-112 | down |
| ENSG00000179241 | LDLRAD3 | low density lipoprotein receptor class A domain containing 3 [Source:HGNC Symbol;Acc:HGNC:27046] | -1.50222 | 9.81E-113 | 6.79E-111 | down |
| ENSG00000125968 | ID1 | inhibitor of DNA binding 1, HLH protein [Source:HGNC Symbol;Acc:HGNC:5360] | -1.31385 | 9.27E-112 | 6.31E-110 | down |
| ENSG00000158747 | NBL1 | neuroblastoma 1, DAN family BMP antagonist [Source:HGNC Symbol;Acc:HGNC:7650] | -1.08637 | 9.86E-111 | 6.61E-109 | down |
| ENSG00000153823 | PID1 | phosphotyrosine interaction domain containing 1 [Source:HGNC Symbol;Acc:HGNC:26084] | -2.50727 | 3.53E-100 | 2.12E-98 | down |
| ENSG00000225339 | AL354740.1 | - | -2.05265 | 6.60E-100 | 3.94E-98 | down |
| ENSG00000178974 | FBXO34 | F-box protein 34 [Source:HGNC Symbol;Acc:HGNC:20201] | -1.94189 | 6.75E-100 | 4.02E-98 | down |
| ENSG00000227706 | AL713998.1 | - | -3.20323 | 7.99E-100 | 4.73E-98 | down |
| ENSG00000221926 | TRIM16 | tripartite motif containing 16 [Source:HGNC Symbol;Acc:HGNC:17241] | -1.21854 | 1.20E-97 | 7.00E-96 | down |
| ENSG00000204599 | TRIM39 | tripartite motif containing 39 [Source:HGNC Symbol;Acc:HGNC:10065] | -1.19462 | 7.31E-96 | 4.15E-94 | down |
| ENSG00000128016 | ZFP36 | ZFP36 ring finger protein [Source:HGNC Symbol;Acc:HGNC:12862] | -1.5846 | 3.01E-93 | 1.64E-91 | down |
| ENSG00000181904 | C5orf24 | chromosome 5 open reading frame 24 [Source:HGNC Symbol;Acc:HGNC:26746] | -1.18302 | 2.17E-92 | 1.17E-90 | down |
| ENSG00000136504 | KAT7 | lysine acetyltransferase 7 [Source:HGNC Symbol;Acc:HGNC:17016] | -1.31537 | 8.39E-87 | 4.29E-85 | down |
| ENSG00000004799 | PDK4 | pyruvate dehydrogenase kinase 4 [Source:HGNC Symbol;Acc:HGNC:8812] | -1.16201 | 1.12E-81 | 5.34E-80 | down |
| ENSG00000174307 | PHLDA3 | pleckstrin homology like domain family A member 3 [Source:HGNC Symbol;Acc:HGNC:8934] | -1.26903 | 9.21E-81 | 4.31E-79 | down |
| ENSG00000130733 | YIPF2 | Yip1 domain family member 2 [Source:HGNC Symbol;Acc:HGNC:28476] | -1.25356 | 1.22E-80 | 5.71E-79 | down |
| ENSG00000144655 | CSRNP1 | cysteine and serine rich nuclear protein 1 [Source:HGNC Symbol;Acc:HGNC:14300] | -1.05785 | 1.23E-80 | 5.75E-79 | down |
| ENSG00000134107 | BHLHE40 | basic helix-loop-helix family member e40 [Source:HGNC Symbol;Acc:HGNC:1046] | -1.01994 | 2.13E-79 | 9.74E-78 | down |
| ENSG00000258529 | AP001781.3 | - | -16.4068 | 2.26E-79 | 1.03E-77 | down |
| ENSG00000267520 | AC010733.2 | - | -2.09602 | 4.20E-78 | 1.89E-76 | down |
| ENSG00000281453 | TGFB2-OT1 | TGFB2 overlapping transcript 1 [Source:HGNC Symbol;Acc:HGNC:50629] | -4.83221 | 1.07E-77 | 4.78E-76 | down |
| ENSG00000214736 | TOMM6 | translocase of outer mitochondrial membrane 6 [Source:HGNC Symbol;Acc:HGNC:34528] | -4.07612 | 2.48E-77 | 1.10E-75 | down |
| ENSG00000003137 | CYP26B1 | cytochrome P450 family 26 subfamily B member 1 [Source:HGNC Symbol;Acc:HGNC:20581] | -1.44479 | 4.63E-76 | 2.00E-74 | down |
| ENSG00000161671 | EMC10 | ER membrane protein complex subunit 10 [Source:HGNC Symbol;Acc:HGNC:27609] | -1.45615 | 1.36E-72 | 5.57E-71 | down |
| ENSG00000011677 | GABRA3 | gamma-aminobutyric acid type A receptor alpha3 subunit [Source:HGNC Symbol;Acc:HGNC:4077] | -2.78554 | 4.16E-72 | 1.70E-70 | down |
| ENSG00000244486 | SCARF2 | scavenger receptor class F member 2 [Source:HGNC Symbol;Acc:HGNC:19869] | -1.01784 | 9.32E-72 | 3.79E-70 | down |
| ENSG00000143061 | IGSF3 | immunoglobulin superfamily member 3 [Source:HGNC Symbol;Acc:HGNC:5950] | -1.82406 | 1.06E-70 | 4.25E-69 | down |
| ENSG00000204604 | ZNF468 | zinc finger protein 468 [Source:HGNC Symbol;Acc:HGNC:33105] | -1.19298 | 1.19E-67 | 4.58E-66 | down |
| ENSG00000145779 | TNFAIP8 | TNF alpha induced protein 8 [Source:HGNC Symbol;Acc:HGNC:17260] | -1.07493 | 5.48E-67 | 2.08E-65 | down |
| ENSG00000125740 | FOSB | FosB proto-oncogene, AP-1 transcription factor subunit [Source:HGNC Symbol;Acc:HGNC:3797] | -2.22473 | 8.41E-67 | 3.19E-65 | down |
| ENSG00000163017 | ACTG2 | actin, gamma 2, smooth muscle, enteric [Source:HGNC Symbol;Acc:HGNC:145] | -2.51247 | 1.85E-66 | 6.98E-65 | down |
| ENSG00000087116 | ADAMTS2 | ADAM metallopeptidase with thrombospondin type 1 motif 2 [Source:HGNC Symbol;Acc:HGNC:218] | -1.12599 | 1.71E-65 | 6.32E-64 | down |
| ENSG00000204642 | HLA-F | major histocompatibility complex, class I, F [Source:HGNC Symbol;Acc:HGNC:4963] | -1.78025 | 3.41E-65 | 1.25E-63 | down |
| ENSG00000164053 | ATRIP | ATR interacting protein [Source:HGNC Symbol;Acc:HGNC:33499] | -1.80381 | 4.40E-65 | 1.61E-63 | down |
| ENSG00000085644 | ZNF213 | zinc finger protein 213 [Source:HGNC Symbol;Acc:HGNC:13005] | -1.49296 | 4.48E-64 | 1.61E-62 | down |
| ENSG00000073849 | ST6GAL1 | ST6 beta-galactoside alpha-2,6-sialyltransferase 1 [Source:HGNC Symbol;Acc:HGNC:10860] | -1.98994 | 7.00E-64 | 2.51E-62 | down |
| ENSG00000132603 | NIP7 | NIP7, nucleolar pre-rRNA processing protein [Source:HGNC Symbol;Acc:HGNC:24328] | -1.26391 | 2.13E-63 | 7.59E-62 | down |
| ENSG00000169139 | UBE2V2 | ubiquitin conjugating enzyme E2 V2 [Source:HGNC Symbol;Acc:HGNC:12495] | -1.72183 | 2.54E-62 | 8.87E-61 | down |
| ENSG00000169241 | SLC50A1 | solute carrier family 50 member 1 [Source:HGNC Symbol;Acc:HGNC:30657] | -1.24005 | 4.92E-61 | 1.69E-59 | down |
| ENSG00000184983 | NDUFA6 | NADH:ubiquinone oxidoreductase subunit A6 [Source:HGNC Symbol;Acc:HGNC:7690] | -1.019 | 1.05E-60 | 3.58E-59 | down |
| ENSG00000011590 | ZBTB32 | zinc finger and BTB domain containing 32 [Source:HGNC Symbol;Acc:HGNC:16763] | -1.02356 | 5.33E-60 | 1.80E-58 | down |
| ENSG00000064300 | NGFR | nerve growth factor receptor [Source:HGNC Symbol;Acc:HGNC:7809] | -1.78087 | 8.96E-60 | 3.02E-58 | down |
| ENSG00000275302 | CCL4 | C-C motif chemokine ligand 4 [Source:HGNC Symbol;Acc:HGNC:10630] | -2.89154 | 1.11E-58 | 3.68E-57 | down |
| ENSG00000175348 | TMEM9B | TMEM9 domain family member B [Source:HGNC Symbol;Acc:HGNC:1168] | -1.08248 | 5.06E-57 | 1.62E-55 | down |
| ENSG00000177427 | MIEF2 | mitochondrial elongation factor 2 [Source:HGNC Symbol;Acc:HGNC:17920] | -1.32326 | 1.31E-56 | 4.14E-55 | down |
| ENSG00000102934 | PLLP | plasmolipin [Source:HGNC Symbol;Acc:HGNC:18553] | -1.76484 | 5.42E-56 | 1.70E-54 | down |
| ENSG00000205423 | CNEP1R1 | CTD nuclear envelope phosphatase 1 regulatory subunit 1 [Source:HGNC Symbol;Acc:HGNC:26759] | -1.099 | 1.36E-55 | 4.24E-54 | down |
| ENSG00000130303 | BST2 | bone marrow stromal cell antigen 2 [Source:HGNC Symbol;Acc:HGNC:1119] | -2.61519 | 5.22E-55 | 1.61E-53 | down |
| ENSG00000248871 | TNFSF12-TNFSF13 | TNFSF12-TNFSF13 readthrough [Source:HGNC Symbol;Acc:HGNC:33537] | -16.3502 | 1.43E-54 | 4.37E-53 | down |
| ENSG00000100985 | MMP9 | matrix metallopeptidase 9 [Source:HGNC Symbol;Acc:HGNC:7176] | -3.35136 | 3.08E-54 | 9.32E-53 | down |
| ENSG00000276070 | CCL4L2 | C-C motif chemokine ligand 4 like 2 [Source:HGNC Symbol;Acc:HGNC:24066] | -1.43123 | 6.66E-54 | 2.00E-52 | down |
| ENSG00000175274 | TP53I11 | tumor protein p53 inducible protein 11 [Source:HGNC Symbol;Acc:HGNC:16842] | -2.01584 | 4.74E-53 | 1.40E-51 | down |
| ENSG00000244005 | NFS1 | NFS1, cysteine desulfurase [Source:HGNC Symbol;Acc:HGNC:15910] | -1.04283 | 1.91E-51 | 5.51E-50 | down |
| ENSG00000095906 | NUBP2 | nucleotide binding protein 2 [Source:HGNC Symbol;Acc:HGNC:8042] | -1.26972 | 2.17E-51 | 6.27E-50 | down |
| ENSG00000168301 | KCTD6 | potassium channel tetramerization domain containing 6 [Source:HGNC Symbol;Acc:HGNC:22235] | -1.75096 | 2.63E-51 | 7.57E-50 | down |
| ENSG00000163159 | VPS72 | vacuolar protein sorting 72 homolog [Source:HGNC Symbol;Acc:HGNC:11644] | -1.36707 | 2.77E-51 | 7.98E-50 | down |
| ENSG00000147419 | CCDC25 | coiled-coil domain containing 25 [Source:HGNC Symbol;Acc:HGNC:25591] | -1.05503 | 3.23E-50 | 9.11E-49 | down |
| ENSG00000164400 | CSF2 | colony stimulating factor 2 [Source:HGNC Symbol;Acc:HGNC:2434] | -1.29453 | 3.20E-49 | 8.90E-48 | down |
| ENSG00000198947 | DMD | dystrophin [Source:HGNC Symbol;Acc:HGNC:2928] | -1.55096 | 7.98E-48 | 2.16E-46 | down |
| ENSG00000267261 | AC099811.2 | - | -3.19175 | 1.52E-47 | 4.12E-46 | down |
| ENSG00000235106 | LINC00094 | long intergenic non-protein coding RNA 94 [Source:HGNC Symbol;Acc:HGNC:24742] | -1.18272 | 3.25E-47 | 8.66E-46 | down |
| ENSG00000226145 | KRT16P6 | keratin 16 pseudogene 6 [Source:HGNC Symbol;Acc:HGNC:50719] | -2.72748 | 3.81E-47 | 1.01E-45 | down |
| ENSG00000284194 | SCO2 | SCO2, cytochrome c oxidase assembly protein [Source:NCBI gene;Acc:9997] | -3.03899 | 8.67E-47 | 2.28E-45 | down |
| ENSG00000162144 | CYB561A3 | cytochrome b561 family member A3 [Source:HGNC Symbol;Acc:HGNC:23014] | -1.01128 | 4.67E-46 | 1.20E-44 | down |
| ENSG00000184205 | TSPYL2 | TSPY like 2 [Source:HGNC Symbol;Acc:HGNC:24358] | -1.3321 | 1.79E-45 | 4.51E-44 | down |
| ENSG00000066056 | TIE1 | tyrosine kinase with immunoglobulin like and EGF like domains 1 [Source:HGNC Symbol;Acc:HGNC:11809] | -1.37687 | 8.49E-44 | 2.05E-42 | down |
| ENSG00000240065 | PSMB9 | proteasome subunit beta 9 [Source:HGNC Symbol;Acc:HGNC:9546] | -1.1968 | 1.38E-43 | 3.32E-42 | down |
| ENSG00000116406 | EDEM3 | ER degradation enhancing alpha-mannosidase like protein 3 [Source:HGNC Symbol;Acc:HGNC:16787] | -1.16741 | 4.75E-43 | 1.13E-41 | down |
| ENSG00000168528 | SERINC2 | serine incorporator 2 [Source:HGNC Symbol;Acc:HGNC:23231] | -1.27733 | 1.13E-42 | 2.65E-41 | down |
| ENSG00000188223 | AD000671.1 | - | -16.6912 | 4.68E-42 | 1.08E-40 | down |
| ENSG00000114315 | HES1 | hes family bHLH transcription factor 1 [Source:HGNC Symbol;Acc:HGNC:5192] | -4.67362 | 1.70E-41 | 3.86E-40 | down |
| ENSG00000269028 | MTRNR2L12 | MT-RNR2-like 12 [Source:HGNC Symbol;Acc:HGNC:37169] | -2.45389 | 2.84E-41 | 6.40E-40 | down |
| ENSG00000216490 | IFI30 | IFI30, lysosomal thiol reductase [Source:HGNC Symbol;Acc:HGNC:5398] | -2.05892 | 8.58E-41 | 1.91E-39 | down |
| ENSG00000187908 | DMBT1 | deleted in malignant brain tumors 1 [Source:HGNC Symbol;Acc:HGNC:2926] | -1.50811 | 1.06E-39 | 2.31E-38 | down |
| ENSG00000074047 | GLI2 | GLI family zinc finger 2 [Source:HGNC Symbol;Acc:HGNC:4318] | -1.34288 | 1.54E-39 | 3.34E-38 | down |
| ENSG00000115944 | COX7A2L | cytochrome c oxidase subunit 7A2 like [Source:HGNC Symbol;Acc:HGNC:2289] | -1.02452 | 3.15E-39 | 6.78E-38 | down |
| ENSG00000265590 | AP000275.2 | - | -4.75472 | 5.47E-38 | 1.15E-36 | down |
| ENSG00000099992 | TBC1D10A | TBC1 domain family member 10A [Source:HGNC Symbol;Acc:HGNC:23609] | -1.18728 | 7.23E-38 | 1.51E-36 | down |
| ENSG00000170345 | FOS | Fos proto-oncogene, AP-1 transcription factor subunit [Source:HGNC Symbol;Acc:HGNC:3796] | -1.43749 | 1.77E-37 | 3.68E-36 | down |
| ENSG00000065989 | PDE4A | phosphodiesterase 4A [Source:HGNC Symbol;Acc:HGNC:8780] | -1.56583 | 2.34E-37 | 4.86E-36 | down |
| ENSG00000185947 | ZNF267 | zinc finger protein 267 [Source:HGNC Symbol;Acc:HGNC:13060] | -1.53485 | 1.12E-36 | 2.29E-35 | down |
| ENSG00000205084 | TMEM231 | transmembrane protein 231 [Source:HGNC Symbol;Acc:HGNC:37234] | -1.38383 | 4.01E-36 | 8.07E-35 | down |
| ENSG00000271853 | AL162258.1 | - | -3.83672 | 1.84E-35 | 3.63E-34 | down |
| ENSG00000258839 | MC1R | melanocortin 1 receptor [Source:HGNC Symbol;Acc:HGNC:6929] | -1.31211 | 2.49E-35 | 4.90E-34 | down |
| ENSG00000076944 | STXBP2 | syntaxin binding protein 2 [Source:HGNC Symbol;Acc:HGNC:11445] | -4.04053 | 6.04E-34 | 1.14E-32 | down |
| ENSG00000166507 | NDST2 | N-deacetylase and N-sulfotransferase 2 [Source:HGNC Symbol;Acc:HGNC:7681] | -1.14181 | 7.29E-34 | 1.37E-32 | down |
| ENSG00000213780 | GTF2H4 | general transcription factor IIH subunit 4 [Source:HGNC Symbol;Acc:HGNC:4658] | -1.3066 | 8.05E-34 | 1.51E-32 | down |
| ENSG00000243989 | ACY1 | aminoacylase 1 [Source:HGNC Symbol;Acc:HGNC:177] | -1.73886 | 1.07E-33 | 2.01E-32 | down |
| ENSG00000278023 | RDM1 | RAD52 motif containing 1 [Source:HGNC Symbol;Acc:HGNC:19950] | -1.47372 | 1.78E-33 | 3.31E-32 | down |
| ENSG00000181773 | GPR3 | G protein-coupled receptor 3 [Source:HGNC Symbol;Acc:HGNC:4484] | -1.03555 | 2.54E-33 | 4.69E-32 | down |
| ENSG00000134470 | IL15RA | interleukin 15 receptor subunit alpha [Source:HGNC Symbol;Acc:HGNC:5978] | -1.2425 | 1.39E-31 | 2.45E-30 | down |
| ENSG00000143171 | RXRG | retinoid X receptor gamma [Source:HGNC Symbol;Acc:HGNC:10479] | -1.6889 | 3.54E-31 | 6.16E-30 | down |
| ENSG00000267248 | AC025048.2 | - | -1.20624 | 2.91E-30 | 4.94E-29 | down |
| ENSG00000134321 | RSAD2 | radical S-adenosyl methionine domain containing 2 [Source:HGNC Symbol;Acc:HGNC:30908] | -1.21439 | 2.18E-29 | 3.60E-28 | down |
| ENSG00000137496 | IL18BP | interleukin 18 binding protein [Source:HGNC Symbol;Acc:HGNC:5987] | -1.07584 | 8.74E-29 | 1.41E-27 | down |
| ENSG00000005189 | REXO5 | RNA exonuclease 5 [Source:HGNC Symbol;Acc:HGNC:24661] | -1.25783 | 1.10E-28 | 1.76E-27 | down |
| ENSG00000125772 | GPCPD1 | glycerophosphocholine phosphodiesterase 1 [Source:HGNC Symbol;Acc:HGNC:26957] | -1.03647 | 1.76E-26 | 2.61E-25 | down |
| ENSG00000073756 | PTGS2 | prostaglandin-endoperoxide synthase 2 [Source:HGNC Symbol;Acc:HGNC:9605] | -1.42645 | 6.78E-26 | 9.83E-25 | down |
| ENSG00000143363 | PRUNE1 | prune exopolyphosphatase 1 [Source:HGNC Symbol;Acc:HGNC:13420] | -1.01713 | 1.56E-24 | 2.16E-23 | down |
| ENSG00000210049 | MT-TF | mitochondrially encoded tRNA phenylalanine [Source:HGNC Symbol;Acc:HGNC:7481] | -1.85982 | 2.05E-24 | 2.81E-23 | down |
| ENSG00000136732 | GYPC | glycophorin C (Gerbich blood group) [Source:HGNC Symbol;Acc:HGNC:4704] | -1.24425 | 2.14E-24 | 2.94E-23 | down |
| ENSG00000162894 | FCMR | Fc fragment of IgM receptor [Source:HGNC Symbol;Acc:HGNC:14315] | -1.7971 | 2.50E-24 | 3.41E-23 | down |
| ENSG00000154553 | PDLIM3 | PDZ and LIM domain 3 [Source:HGNC Symbol;Acc:HGNC:20767] | -1.5977 | 5.58E-24 | 7.53E-23 | down |
| ENSG00000175482 | POLD4 | DNA polymerase delta 4, accessory subunit [Source:HGNC Symbol;Acc:HGNC:14106] | -1.0183 | 6.99E-24 | 9.41E-23 | down |
| ENSG00000185808 | PIGP | phosphatidylinositol glycan anchor biosynthesis class P [Source:HGNC Symbol;Acc:HGNC:3046] | -1.09366 | 1.07E-23 | 1.43E-22 | down |
| ENSG00000255526 | NEDD8-MDP1 | NEDD8-MDP1 readthrough [Source:HGNC Symbol;Acc:HGNC:39551] | -3.10616 | 3.63E-23 | 4.73E-22 | down |
| ENSG00000162194 | LBHD1 | LBH domain containing 1 [Source:HGNC Symbol;Acc:HGNC:28351] | -1.22005 | 7.51E-23 | 9.65E-22 | down |
| ENSG00000163795 | ZNF513 | zinc finger protein 513 [Source:HGNC Symbol;Acc:HGNC:26498] | -1.36526 | 9.88E-23 | 1.26E-21 | down |
| ENSG00000198832 | SELENOM | selenoprotein M [Source:HGNC Symbol;Acc:HGNC:30397] | -1.03511 | 1.48E-22 | 1.87E-21 | down |
| ENSG00000250021 | C15orf38-AP3S2 | C15orf38-AP3S2 readthrough [Source:HGNC Symbol;Acc:HGNC:38824] | -3.00664 | 1.72E-22 | 2.16E-21 | down |
| ENSG00000268916 | CSAG3 | CSAG family member 3 [Source:HGNC Symbol;Acc:HGNC:26237] | -1.40788 | 1.82E-22 | 2.30E-21 | down |
| ENSG00000280123 | AC023632.6 | - | -1.16328 | 8.50E-22 | 1.03E-20 | down |
| ENSG00000113532 | ST8SIA4 | ST8 alpha-N-acetyl-neuraminide alpha-2,8-sialyltransferase 4 [Source:HGNC Symbol;Acc:HGNC:10871] | -1.14247 | 1.48E-21 | 1.78E-20 | down |
| ENSG00000244270 | AL139099.1 | - | -3.89303 | 2.58E-21 | 3.08E-20 | down |
| ENSG00000179532 | DNHD1 | dynein heavy chain domain 1 [Source:HGNC Symbol;Acc:HGNC:26532] | -1.17685 | 1.06E-20 | 1.23E-19 | down |
| ENSG00000283149 | AC068631.2 | - | -16.4562 | 1.82E-20 | 2.09E-19 | down |
| ENSG00000164430 | MB21D1 | Mab-21 domain containing 1 [Source:HGNC Symbol;Acc:HGNC:21367] | -1.2509 | 3.43E-20 | 3.90E-19 | down |
| ENSG00000258653 | AC005520.1 | - | -1.04576 | 5.48E-20 | 6.18E-19 | down |
| ENSG00000174206 | C12orf66 | chromosome 12 open reading frame 66 [Source:HGNC Symbol;Acc:HGNC:26517] | -1.10367 | 6.94E-20 | 7.78E-19 | down |
| ENSG00000115919 | KYNU | kynureninase [Source:HGNC Symbol;Acc:HGNC:6469] | -3.43053 | 1.58E-19 | 1.75E-18 | down |
| ENSG00000283936 | MIR3658 | microRNA 3658 [Source:HGNC Symbol;Acc:HGNC:38963] | -7.92408 | 2.41E-19 | 2.64E-18 | down |
| ENSG00000147003 | TMEM27 | transmembrane protein 27 [Source:HGNC Symbol;Acc:HGNC:29437] | -1.17173 | 6.03E-19 | 6.48E-18 | down |
| ENSG00000268902 | CSAG2 | CSAG family member 2 [Source:HGNC Symbol;Acc:HGNC:16847] | -1.66685 | 7.77E-19 | 8.32E-18 | down |
| ENSG00000159208 | CIART | circadian associated repressor of transcription [Source:HGNC Symbol;Acc:HGNC:25200] | -1.58638 | 2.19E-18 | 2.30E-17 | down |
| ENSG00000128965 | CHAC1 | ChaC glutathione specific gamma-glutamylcyclotransferase 1 [Source:HGNC Symbol;Acc:HGNC:28680] | -1.14258 | 7.64E-18 | 7.86E-17 | down |
| ENSG00000123154 | WDR83 | WD repeat domain 83 [Source:HGNC Symbol;Acc:HGNC:32672] | -1.03629 | 8.68E-18 | 8.90E-17 | down |
| ENSG00000132749 | TESMIN | testis expressed metallothionein like protein [Source:HGNC Symbol;Acc:HGNC:7446] | -1.0085 | 1.74E-17 | 1.76E-16 | down |
| ENSG00000170498 | KISS1 | KiSS-1 metastasis-suppressor [Source:HGNC Symbol;Acc:HGNC:6341] | -1.45733 | 2.08E-17 | 2.10E-16 | down |
| ENSG00000243207 | PPAN-P2RY11 | PPAN-P2RY11 readthrough [Source:HGNC Symbol;Acc:HGNC:33526] | -1.02394 | 4.37E-17 | 4.36E-16 | down |
| ENSG00000262481 | TMEM256-PLSCR3 | TMEM256-PLSCR3 readthrough (NMD candidate) [Source:HGNC Symbol;Acc:HGNC:49186] | -1.05077 | 4.86E-17 | 4.83E-16 | down |
| ENSG00000260238 | PMF1-BGLAP | PMF1-BGLAP readthrough [Source:HGNC Symbol;Acc:HGNC:42953] | -1.17718 | 7.34E-17 | 7.21E-16 | down |
| ENSG00000228470 | AL929236.1 | - | -2.94126 | 1.36E-16 | 1.32E-15 | down |
| ENSG00000187689 | AMTN | amelotin [Source:HGNC Symbol;Acc:HGNC:33188] | -2.91834 | 2.92E-16 | 2.77E-15 | down |
| ENSG00000141198 | TOM1L1 | target of myb1 like 1 membrane trafficking protein [Source:HGNC Symbol;Acc:HGNC:11983] | -2.8941 | 5.68E-16 | 5.30E-15 | down |
| ENSG00000142677 | IL22RA1 | interleukin 22 receptor subunit alpha 1 [Source:HGNC Symbol;Acc:HGNC:13700] | -1.05754 | 8.19E-16 | 7.59E-15 | down |
| ENSG00000246705 | H2AFJ | H2A histone family member J [Source:HGNC Symbol;Acc:HGNC:14456] | -1.76578 | 1.18E-15 | 1.08E-14 | down |
| ENSG00000231503 | PTMAP4 | prothymosin, alpha pseudogene 4 [Source:HGNC Symbol;Acc:HGNC:9627] | -1.21541 | 2.25E-15 | 2.03E-14 | down |
| ENSG00000114735 | HEMK1 | HemK methyltransferase family member 1 [Source:HGNC Symbol;Acc:HGNC:24923] | -1.1557 | 2.54E-15 | 2.29E-14 | down |
| ENSG00000142046 | TMEM91 | transmembrane protein 91 [Source:HGNC Symbol;Acc:HGNC:32393] | -1.34886 | 1.26E-14 | 1.09E-13 | down |
| ENSG00000111012 | CYP27B1 | cytochrome P450 family 27 subfamily B member 1 [Source:HGNC Symbol;Acc:HGNC:2606] | -1.07061 | 4.27E-14 | 3.59E-13 | down |
| ENSG00000184281 | TSSC4 | tumor suppressing subtransferable candidate 4 [Source:HGNC Symbol;Acc:HGNC:12386] | -1.18929 | 6.46E-14 | 5.36E-13 | down |
| ENSG00000230002 | ALMS1-IT1 | ALMS1 intronic transcript 1 [Source:HGNC Symbol;Acc:HGNC:41305] | -1.30998 | 9.25E-14 | 7.59E-13 | down |
| ENSG00000166920 | C15orf48 | chromosome 15 open reading frame 48 [Source:HGNC Symbol;Acc:HGNC:29898] | -1.8012 | 1.21E-13 | 9.87E-13 | down |
| ENSG00000184979 | USP18 | ubiquitin specific peptidase 18 [Source:HGNC Symbol;Acc:HGNC:12616] | -1.06431 | 1.36E-13 | 1.10E-12 | down |
| ENSG00000072818 | ACAP1 | ArfGAP with coiled-coil, ankyrin repeat and PH domains 1 [Source:HGNC Symbol;Acc:HGNC:16467] | -1.93607 | 1.36E-13 | 1.10E-12 | down |
| ENSG00000273291 | AC092042.3 | - | -2.20025 | 1.96E-13 | 1.57E-12 | down |
| ENSG00000110944 | IL23A | interleukin 23 subunit alpha [Source:HGNC Symbol;Acc:HGNC:15488] | -2.08812 | 3.09E-13 | 2.43E-12 | down |
| ENSG00000115009 | CCL20 | C-C motif chemokine ligand 20 [Source:HGNC Symbol;Acc:HGNC:10619] | -1.9528 | 1.22E-12 | 9.27E-12 | down |
| ENSG00000162526 | TSSK3 | testis specific serine kinase 3 [Source:HGNC Symbol;Acc:HGNC:15473] | -1.62174 | 1.28E-12 | 9.69E-12 | down |
| ENSG00000198598 | MMP17 | matrix metallopeptidase 17 [Source:HGNC Symbol;Acc:HGNC:7163] | -3.2709 | 1.43E-12 | 1.08E-11 | down |
| ENSG00000232859 | LYRM9 | LYR motif containing 9 [Source:HGNC Symbol;Acc:HGNC:27314] | -1.5438 | 2.55E-12 | 1.89E-11 | down |
| ENSG00000203644 | AC083799.1 | - | -1.10866 | 2.98E-12 | 2.20E-11 | down |
| ENSG00000258232 | AC125611.3 | - | -2.48432 | 6.31E-12 | 4.56E-11 | down |
| ENSG00000008517 | IL32 | interleukin 32 [Source:HGNC Symbol;Acc:HGNC:16830] | -1.55198 | 1.09E-11 | 7.71E-11 | down |
| ENSG00000163132 | MSX1 | msh homeobox 1 [Source:HGNC Symbol;Acc:HGNC:7391] | -1.39056 | 1.20E-11 | 8.46E-11 | down |
| ENSG00000186567 | CEACAM19 | carcinoembryonic antigen related cell adhesion molecule 19 [Source:HGNC Symbol;Acc:HGNC:31951] | -1.10161 | 1.24E-11 | 8.76E-11 | down |
| ENSG00000143429 | AC116050.1 | - | -1.76294 | 1.32E-11 | 9.28E-11 | down |
| ENSG00000089127 | OAS1 | 2'-5'-oligoadenylate synthetase 1 [Source:HGNC Symbol;Acc:HGNC:8086] | -1.18383 | 1.56E-11 | 1.10E-10 | down |
| ENSG00000250748 | AC025419.1 | - | -1.14347 | 2.38E-11 | 1.66E-10 | down |
| ENSG00000275121 | AC211486.4 | - | -5.91908 | 6.57E-11 | 4.43E-10 | down |
| ENSG00000239332 | LINC01119 | long intergenic non-protein coding RNA 1119 [Source:HGNC Symbol;Acc:HGNC:49262] | -1.8034 | 1.02E-10 | 6.81E-10 | down |
| ENSG00000238917 | SNORD10 | small nucleolar RNA, C/D box 10 [Source:HGNC Symbol;Acc:HGNC:32706] | -1.20116 | 1.34E-10 | 8.83E-10 | down |
| ENSG00000257949 | TEN1 | TEN1, CST complex subunit [Source:HGNC Symbol;Acc:HGNC:37242] | -1.90604 | 1.40E-10 | 9.27E-10 | down |
| ENSG00000101017 | CD40 | CD40 molecule [Source:HGNC Symbol;Acc:HGNC:11919] | -1.00457 | 1.62E-10 | 1.07E-09 | down |
| ENSG00000137198 | GMPR | guanosine monophosphate reductase [Source:HGNC Symbol;Acc:HGNC:4376] | -1.02047 | 2.01E-10 | 1.31E-09 | down |
| ENSG00000156500 | FAM122C | family with sequence similarity 122C [Source:HGNC Symbol;Acc:HGNC:25202] | -1.05515 | 2.03E-10 | 1.32E-09 | down |
| ENSG00000186866 | POFUT2 | protein O-fucosyltransferase 2 [Source:HGNC Symbol;Acc:HGNC:14683] | -1.07567 | 2.85E-10 | 1.84E-09 | down |
| ENSG00000227220 | AL133346.1 | - | -1.54486 | 3.51E-10 | 2.25E-09 | down |
| ENSG00000142632 | ARHGEF19 | Rho guanine nucleotide exchange factor 19 [Source:HGNC Symbol;Acc:HGNC:26604] | -1.02322 | 5.37E-10 | 3.40E-09 | down |
| ENSG00000278334 | HOXA11-AS1_2 | HOXA11 antisense RNA 1 conserved region 2 [Source:RFAM;Acc:RF02138] | -5.55291 | 6.84E-10 | 4.29E-09 | down |
| ENSG00000283196 | AC006453.2 | - | -1.07617 | 7.18E-10 | 4.50E-09 | down |
| ENSG00000267598 | AC011446.2 | - | -2.79238 | 1.42E-09 | 8.67E-09 | down |
| ENSG00000145088 | EAF2 | ELL associated factor 2 [Source:HGNC Symbol;Acc:HGNC:23115] | -1.6502 | 1.48E-09 | 9.04E-09 | down |
| ENSG00000171970 | ZNF57 | zinc finger protein 57 [Source:HGNC Symbol;Acc:HGNC:13125] | -1.0533 | 1.48E-09 | 9.04E-09 | down |
| ENSG00000139725 | RHOF | ras homolog family member F, filopodia associated [Source:HGNC Symbol;Acc:HGNC:15703] | -1.08739 | 2.36E-09 | 1.41E-08 | down |
| ENSG00000092096 | SLC22A17 | solute carrier family 22 member 17 [Source:HGNC Symbol;Acc:HGNC:23095] | -1.02608 | 2.79E-09 | 1.67E-08 | down |
| ENSG00000172250 | SERHL | serine hydrolase-like (pseudogene) [Source:HGNC Symbol;Acc:HGNC:14408] | -2.00502 | 3.15E-09 | 1.87E-08 | down |
| ENSG00000226067 | LINC00623 | long intergenic non-protein coding RNA 623 [Source:HGNC Symbol;Acc:HGNC:44252] | -1.7566 | 8.13E-09 | 4.66E-08 | down |
| ENSG00000150455 | TIRAP | TIR domain containing adaptor protein [Source:HGNC Symbol;Acc:HGNC:17192] | -1.04121 | 1.48E-08 | 8.32E-08 | down |
| ENSG00000140876 | NUDT7 | nudix hydrolase 7 [Source:HGNC Symbol;Acc:HGNC:8054] | -1.31188 | 1.68E-08 | 9.34E-08 | down |
| ENSG00000281348 | AC120114.5 | - | -1.4746 | 2.48E-08 | 1.37E-07 | down |
| ENSG00000186787 | SPIN2B | spindlin family member 2B [Source:HGNC Symbol;Acc:HGNC:33147] | -1.40943 | 2.90E-08 | 1.58E-07 | down |
| ENSG00000204397 | CARD16 | caspase recruitment domain family member 16 [Source:HGNC Symbol;Acc:HGNC:33701] | -1.06861 | 2.98E-08 | 1.62E-07 | down |
| ENSG00000005961 | ITGA2B | integrin subunit alpha 2b [Source:HGNC Symbol;Acc:HGNC:6138] | -1.0476 | 4.94E-08 | 2.66E-07 | down |
| ENSG00000157895 | C12orf43 | chromosome 12 open reading frame 43 [Source:HGNC Symbol;Acc:HGNC:25719] | -1.0497 | 5.75E-08 | 3.07E-07 | down |
| ENSG00000226221 | AC022431.1 | - | -1.52167 | 1.17E-07 | 6.04E-07 | down |
| ENSG00000153291 | SLC25A27 | solute carrier family 25 member 27 [Source:HGNC Symbol;Acc:HGNC:21065] | -1.52322 | 1.26E-07 | 6.44E-07 | down |
| ENSG00000198930 | CSAG1 | chondrosarcoma associated gene 1 [Source:HGNC Symbol;Acc:HGNC:24294] | -1.86047 | 1.58E-07 | 7.99E-07 | down |
| ENSG00000224389 | C4B | complement C4B (Chido blood group) [Source:HGNC Symbol;Acc:HGNC:1324] | -1.0781 | 2.08E-07 | 1.04E-06 | down |
| ENSG00000172974 | AC007318.1 | - | -1.04756 | 2.48E-07 | 1.23E-06 | down |
| ENSG00000254452 | AP001107.2 | - | -1.20359 | 4.11E-07 | 2.00E-06 | down |
| ENSG00000235508 | RPS2P7 | ribosomal protein S2 pseudogene 7 [Source:HGNC Symbol;Acc:HGNC:15838] | -1.21394 | 4.97E-07 | 2.40E-06 | down |
| ENSG00000122550 | KLHL7 | kelch like family member 7 [Source:HGNC Symbol;Acc:HGNC:15646] | -1.06658 | 6.92E-07 | 3.30E-06 | down |
| ENSG00000217716 | RPS10P3 | ribosomal protein S10 pseudogene 3 [Source:HGNC Symbol;Acc:HGNC:23684] | -1.11898 | 8.50E-07 | 4.02E-06 | down |
| ENSG00000260615 | RPL23AP97 | ribosomal protein L23a pseudogene 97 [Source:HGNC Symbol;Acc:HGNC:51632] | -1.43051 | 2.15E-06 | 9.69E-06 | down |
| ENSG00000125726 | CD70 | CD70 molecule [Source:HGNC Symbol;Acc:HGNC:11937] | -1.19929 | 3.09E-06 | 1.37E-05 | down |
| ENSG00000200087 | SNORA73B | small nucleolar RNA, H/ACA box 73B [Source:HGNC Symbol;Acc:HGNC:10116] | -1.13407 | 3.96E-06 | 1.73E-05 | down |
| ENSG00000272275 | AC092687.3 | - | -2.19042 | 7.36E-06 | 3.12E-05 | down |
| ENSG00000256967 | AC018653.3 | - | -1.0405 | 7.62E-06 | 3.22E-05 | down |
| ENSG00000267952 | AC008878.1 | - | -1.34924 | 7.69E-06 | 3.25E-05 | down |
| ENSG00000229953 | AL590666.2 | - | -1.24115 | 1.21E-05 | 4.99E-05 | down |
| ENSG00000209480 | SNORD83B | small nucleolar RNA, C/D box 83B [Source:HGNC Symbol;Acc:HGNC:17132] | -2.65879 | 1.98E-05 | 7.99E-05 | down |
| ENSG00000254837 | AP001372.2 | - | -1.05824 | 2.46E-05 | 9.80E-05 | down |
| ENSG00000209482 | SNORD83A | small nucleolar RNA, C/D box 83A [Source:HGNC Symbol;Acc:HGNC:17131] | -2.7326 | 3.47E-05 | 0.000135 | down |
| ENSG00000199785 | SNORA52 | small nucleolar RNA, H/ACA box 52 [Source:HGNC Symbol;Acc:HGNC:32645] | -3.29966 | 6.13E-05 | 0.000231 | down |
| ENSG00000269906 | AL606834.2 | - | -1.19482 | 7.21E-05 | 0.000269 | down |
| ENSG00000281808 | SNORA17 | Small nucleolar RNA SNORA17 [Source:RFAM;Acc:RF00560] | -4.90249 | 7.31E-05 | 0.000273 | down |
| ENSG00000187953 | PMS2CL | PMS2 C-terminal like pseudogene [Source:HGNC Symbol;Acc:HGNC:30061] | -1.02941 | 9.94E-05 | 0.000364 | down |
| ENSG00000199133 | MIRLET7D | microRNA let-7d [Source:HGNC Symbol;Acc:HGNC:31481] | -3.69833 | 0.000113 | 0.000412 | down |
| ENSG00000265749 | AC135178.3 | - | -1.47523 | 0.000115 | 0.000416 | down |
| ENSG00000200534 | SNORA33 | small nucleolar RNA, H/ACA box 33 [Source:HGNC Symbol;Acc:HGNC:32623] | -1.85217 | 0.000124 | 0.000447 | down |
| ENSG00000204652 | RPS26P8 | ribosomal protein S26 pseudogene 8 [Source:HGNC Symbol;Acc:HGNC:31329] | -1.08509 | 0.000126 | 0.000453 | down |
| ENSG00000225920 | RIMKLBP2 | ribosomal modification protein rimK like family member B pseudogene 2 [Source:HGNC Symbol;Acc:HGNC:39163] | -1.07868 | 0.000126 | 0.000453 | down |
| ENSG00000264346 | SNORA77B | small nucleolar RNA, H/ACA box 77B [Source:HGNC Symbol;Acc:HGNC:52221] | -6.70415 | 0.000138 | 0.000493 | down |
| ENSG00000224858 | RPL29P11 | ribosomal protein L29 pseudogene 11 [Source:HGNC Symbol;Acc:HGNC:36905] | -1.29131 | 0.00014 | 0.000501 | down |
| ENSG00000234648 | AL162151.2 | - | -1.07038 | 0.000161 | 0.000573 | down |
| ENSG00000227591 | AL031316.1 | - | -1.52928 | 0.000171 | 0.000605 | down |
| ENSG00000222365 | SNORD12B | small nucleolar RNA, C/D box 12B [Source:HGNC Symbol;Acc:HGNC:33573] | -1.65684 | 0.000233 | 0.000808 | down |
| ENSG00000253320 | AZIN1-AS1 | AZIN1 antisense RNA 1 [Source:HGNC Symbol;Acc:HGNC:51558] | -1.06005 | 0.000415 | 0.001382 | down |
| ENSG00000272273 | AL662797.2 | - | -1.22776 | 0.000507 | 0.001665 | down |
| ENSG00000227615 | AP001324.1 | - | -1.20977 | 0.000723 | 0.002317 | down |
| ENSG00000111863 | ADTRP | androgen dependent TFPI regulating protein [Source:HGNC Symbol;Acc:HGNC:21214] | -1.14858 | 0.000768 | 0.002449 | down |
| ENSG00000197483 | ZNF628 | zinc finger protein 628 [Source:HGNC Symbol;Acc:HGNC:28054] | -1.15678 | 0.000907 | 0.002851 | down |
| ENSG00000263847 | AP005899.1 | - | -1.10949 | 0.000912 | 0.002865 | down |
| ENSG00000224094 | RPS24P8 | ribosomal protein S24 pseudogene 8 [Source:HGNC Symbol;Acc:HGNC:37016] | -1.29366 | 0.000928 | 0.00291 | down |
| ENSG00000225808 | DNAJC19P5 | DnaJ heat shock protein family (Hsp40) member C19 pseudogene 5 [Source:HGNC Symbol;Acc:HGNC:45068] | -1.47936 | 0.00108 | 0.003349 | down |
| ENSG00000196337 | CGB7 | chorionic gonadotropin beta subunit 7 [Source:HGNC Symbol;Acc:HGNC:16451] | -2.13302 | 0.001383 | 0.004202 | down |
| ENSG00000220988 | SNORD88C | small nucleolar RNA, C/D box 88C [Source:HGNC Symbol;Acc:HGNC:32749] | -5.18804 | 0.001756 | 0.005234 | down |
| ENSG00000277945 | AC107308.1 | - | -1.52862 | 0.001821 | 0.005415 | down |
| ENSG00000272669 | AL021707.6 | - | -1.01999 | 0.002212 | 0.006467 | down |
| ENSG00000160408 | ST6GALNAC6 | ST6 N-acetylgalactosaminide alpha-2,6-sialyltransferase 6 [Source:HGNC Symbol;Acc:HGNC:23364] | -1.00737 | 0.002374 | 0.006896 | down |
| ENSG00000198088 | NUP62CL | nucleoporin 62 C-terminal like [Source:HGNC Symbol;Acc:HGNC:25960] | -1.0069 | 0.002673 | 0.007667 | down |
| ENSG00000214922 | HLA-F-AS1 | HLA-F antisense RNA 1 [Source:HGNC Symbol;Acc:HGNC:26645] | -1.06793 | 0.004018 | 0.011137 | down |
| ENSG00000207725 | MIR222 | microRNA 222 [Source:HGNC Symbol;Acc:HGNC:31602] | -2.30377 | 0.004663 | 0.012728 | down |
| ENSG00000165714 | BORCS5 | BLOC-1 related complex subunit 5 [Source:HGNC Symbol;Acc:HGNC:17950] | -1.13047 | 0.004808 | 0.013091 | down |
| ENSG00000207757 | MIR93 | microRNA 93 [Source:HGNC Symbol;Acc:HGNC:31645] | -1.97472 | 0.007045 | 0.018407 | down |
| ENSG00000212175 | SNORA12 | Small nucleolar RNA SNORA12 [Source:RFAM;Acc:RF00586] | -2.18723 | 0.007748 | 0.020076 | down |
| ENSG00000187186 | AL162231.1 | - | -1.41263 | 0.008532 | 0.02191 | down |
| ENSG00000188732 | FAM221A | family with sequence similarity 221 member A [Source:HGNC Symbol;Acc:HGNC:27977] | -1.1712 | 0.011174 | 0.027826 | down |
| ENSG00000200913 | SNORD46 | small nucleolar RNA, C/D box 46 [Source:HGNC Symbol;Acc:HGNC:10186] | -2.05479 | 0.012998 | 0.031789 | down |
| ENSG00000207047 | SNORD51 | small nucleolar RNA, C/D box 51 [Source:HGNC Symbol;Acc:HGNC:10201] | -1.87865 | 0.012998 | 0.031789 | down |
| ENSG00000199477 | SNORA31 | small nucleolar RNA, H/ACA box 31 [Source:HGNC Symbol;Acc:HGNC:32621] | -1.58295 | 0.013406 | 0.032654 | down |
| ENSG00000023228 | NDUFS1 | NADH:ubiquinone oxidoreductase core subunit S1 [Source:HGNC Symbol;Acc:HGNC:7707] | -1.38393 | 0.017047 | 0.040546 | down |
| ENSG00000284520 | MIRLET7B | microRNA let-7b [Source:HGNC Symbol;Acc:HGNC:31479] | -1.67799 | 0.018715 | 0.043997 | down |
| ENSG00000198938 | MT-CO3 | mitochondrially encoded cytochrome c oxidase III [Source:HGNC Symbol;Acc:HGNC:7422] | 1.405187 | 0 | 0 | up |
| ENSG00000198727 | MT-CYB | mitochondrially encoded cytochrome b [Source:HGNC Symbol;Acc:HGNC:7427] | 1.171702 | 0 | 0 | up |
| ENSG00000248527 | MTATP6P1 | mitochondrially encoded ATP synthase 6 pseudogene 1 [Source:HGNC Symbol;Acc:HGNC:44575] | 1.129035 | 0 | 0 | up |
| ENSG00000198840 | MT-ND3 | mitochondrially encoded NADH:ubiquinone oxidoreductase core subunit 3 [Source:HGNC Symbol;Acc:HGNC:7458] | 1.151718 | 0 | 0 | up |
| ENSG00000164924 | YWHAZ | tyrosine 3-monooxygenase/tryptophan 5-monooxygenase activation protein zeta [Source:HGNC Symbol;Acc:HGNC:12855] | 1.599827 | 0 | 0 | up |
| ENSG00000108821 | COL1A1 | collagen type I alpha 1 chain [Source:HGNC Symbol;Acc:HGNC:2197] | 1.044516 | 0 | 0 | up |
| ENSG00000166913 | YWHAB | tyrosine 3-monooxygenase/tryptophan 5-monooxygenase activation protein beta [Source:HGNC Symbol;Acc:HGNC:12849] | 1.7442 | 0 | 0 | up |
| ENSG00000182158 | CREB3L2 | cAMP responsive element binding protein 3 like 2 [Source:HGNC Symbol;Acc:HGNC:23720] | 1.66724 | 0 | 0 | up |
| ENSG00000115963 | RND3 | Rho family GTPase 3 [Source:HGNC Symbol;Acc:HGNC:671] | 1.038119 | 0 | 0 | up |
| ENSG00000151835 | SACS | sacsin molecular chaperone [Source:HGNC Symbol;Acc:HGNC:10519] | 1.774004 | 0 | 0 | up |
| ENSG00000063978 | RNF4 | ring finger protein 4 [Source:HGNC Symbol;Acc:HGNC:10067] | 1.310572 | 0 | 0 | up |
| ENSG00000116962 | NID1 | nidogen 1 [Source:HGNC Symbol;Acc:HGNC:7821] | 1.283386 | 0 | 0 | up |
| ENSG00000178878 | APOLD1 | apolipoprotein L domain containing 1 [Source:HGNC Symbol;Acc:HGNC:25268] | 3.234942 | 0 | 0 | up |
| ENSG00000104765 | BNIP3L | BCL2 interacting protein 3 like [Source:HGNC Symbol;Acc:HGNC:1085] | 1.576007 | 0 | 0 | up |
| ENSG00000164171 | ITGA2 | integrin subunit alpha 2 [Source:HGNC Symbol;Acc:HGNC:6137] | 1.168748 | 0 | 0 | up |
| ENSG00000107984 | DKK1 | dickkopf WNT signaling pathway inhibitor 1 [Source:HGNC Symbol;Acc:HGNC:2891] | 1.223646 | 0 | 0 | up |
| ENSG00000283782 | AC116366.3 | - | 5.380814 | 0 | 0 | up |
| ENSG00000183291 | SELENOF | selenoprotein F [Source:HGNC Symbol;Acc:HGNC:17705] | 2.367062 | 0 | 0 | up |
| ENSG00000225690 | TREML5P | triggering receptor expressed on myeloid cells like 5, pseudogene [Source:HGNC Symbol;Acc:HGNC:30808] | 22.48673 | 0 | 0 | up |
| ENSG00000146674 | IGFBP3 | insulin like growth factor binding protein 3 [Source:HGNC Symbol;Acc:HGNC:5472] | 2.010134 | 0 | 0 | up |
| ENSG00000125841 | NRSN2 | neurensin 2 [Source:HGNC Symbol;Acc:HGNC:16229] | 1.91753 | 0 | 0 | up |
| ENSG00000284461 | RABGEF1 | RAB guanine nucleotide exchange factor 1 [Source:NCBI gene;Acc:27342] | 3.140668 | 0 | 0 | up |
| ENSG00000198561 | CTNND1 | catenin delta 1 [Source:HGNC Symbol;Acc:HGNC:2515] | 1.119713 | 0 | 0 | up |
| ENSG00000124767 | GLO1 | glyoxalase I [Source:HGNC Symbol;Acc:HGNC:4323] | 1.009516 | 0 | 0 | up |
| ENSG00000127314 | RAP1B | RAP1B, member of RAS oncogene family [Source:HGNC Symbol;Acc:HGNC:9857] | 2.213127 | 0 | 0 | up |
| ENSG00000111799 | COL12A1 | collagen type XII alpha 1 chain [Source:HGNC Symbol;Acc:HGNC:2188] | 1.04749 | 0 | 0 | up |
| ENSG00000163395 | IGFN1 | immunoglobulin-like and fibronectin type III domain containing 1 [Source:HGNC Symbol;Acc:HGNC:24607] | 1.300407 | 0 | 0 | up |
| ENSG00000187446 | CHP1 | calcineurin like EF-hand protein 1 [Source:HGNC Symbol;Acc:HGNC:17433] | 1.618361 | 0 | 0 | up |
| ENSG00000105778 | AVL9 | AVL9 cell migration associated [Source:HGNC Symbol;Acc:HGNC:28994] | 1.284349 | 4.91E-298 | 8.56E-296 | up |
| ENSG00000206053 | JPT2 | Jupiter microtubule associated homolog 2 [Source:HGNC Symbol;Acc:HGNC:14137] | 1.536789 | 4.11E-293 | 7.02E-291 | up |
| ENSG00000168386 | FILIP1L | filamin A interacting protein 1 like [Source:HGNC Symbol;Acc:HGNC:24589] | 2.168587 | 1.61E-270 | 2.62E-268 | up |
| ENSG00000143970 | ASXL2 | additional sex combs like 2, transcriptional regulator [Source:HGNC Symbol;Acc:HGNC:23805] | 1.408176 | 7.21E-268 | 1.17E-265 | up |
| ENSG00000111652 | COPS7A | COP9 signalosome subunit 7A [Source:HGNC Symbol;Acc:HGNC:16758] | 1.46324 | 3.99E-257 | 6.41E-255 | up |
| ENSG00000253873 | PCDHGA11 | protocadherin gamma subfamily A, 11 [Source:HGNC Symbol;Acc:HGNC:8698] | 3.082981 | 1.07E-246 | 1.61E-244 | up |
| ENSG00000136997 | MYC | MYC proto-oncogene, bHLH transcription factor [Source:HGNC Symbol;Acc:HGNC:7553] | 2.253284 | 3.71E-246 | 5.52E-244 | up |
| ENSG00000197757 | HOXC6 | homeobox C6 [Source:HGNC Symbol;Acc:HGNC:5128] | 2.679145 | 1.17E-243 | 1.73E-241 | up |
| ENSG00000168497 | CAVIN2 | caveolae associated protein 2 [Source:HGNC Symbol;Acc:HGNC:10690] | 1.381506 | 2.37E-236 | 3.43E-234 | up |
| ENSG00000169851 | PCDH7 | protocadherin 7 [Source:HGNC Symbol;Acc:HGNC:8659] | 1.571817 | 5.46E-223 | 7.46E-221 | up |
| ENSG00000267680 | ZNF224 | zinc finger protein 224 [Source:HGNC Symbol;Acc:HGNC:13017] | 2.283504 | 1.45E-190 | 1.70E-188 | up |
| ENSG00000123091 | RNF11 | ring finger protein 11 [Source:HGNC Symbol;Acc:HGNC:10056] | 1.005815 | 1.86E-190 | 2.17E-188 | up |
| ENSG00000134762 | DSC3 | desmocollin 3 [Source:HGNC Symbol;Acc:HGNC:3037] | 1.255199 | 1.28E-188 | 1.48E-186 | up |
| ENSG00000109572 | CLCN3 | chloride voltage-gated channel 3 [Source:HGNC Symbol;Acc:HGNC:2021] | 1.092145 | 4.03E-184 | 4.60E-182 | up |
| ENSG00000270181 | BIVM-ERCC5 | BIVM-ERCC5 readthrough [Source:HGNC Symbol;Acc:HGNC:43690] | 3.588456 | 3.29E-179 | 3.67E-177 | up |
| ENSG00000132561 | MATN2 | matrilin 2 [Source:HGNC Symbol;Acc:HGNC:6908] | 1.18149 | 6.75E-178 | 7.42E-176 | up |
| ENSG00000154678 | PDE1C | phosphodiesterase 1C [Source:HGNC Symbol;Acc:HGNC:8776] | 1.67486 | 6.16E-170 | 6.54E-168 | up |
| ENSG00000118495 | PLAGL1 | PLAG1 like zinc finger 1 [Source:HGNC Symbol;Acc:HGNC:9046] | 1.253078 | 6.43E-161 | 6.33E-159 | up |
| ENSG00000260772 | AC012321.1 | - | 19.27596 | 7.62E-161 | 7.48E-159 | up |
| ENSG00000187210 | GCNT1 | glucosaminyl (N-acetyl) transferase 1, core 2 [Source:HGNC Symbol;Acc:HGNC:4203] | 1.293838 | 1.74E-160 | 1.70E-158 | up |
| ENSG00000134590 | RTL8C | retrotransposon Gag like 8C [Source:HGNC Symbol;Acc:HGNC:2569] | 1.177431 | 1.95E-159 | 1.89E-157 | up |
| ENSG00000106484 | MEST | mesoderm specific transcript [Source:HGNC Symbol;Acc:HGNC:7028] | 1.576068 | 1.28E-151 | 1.19E-149 | up |
| ENSG00000283515 | AC020915.6 | - | 2.858175 | 4.10E-141 | 3.57E-139 | up |
| ENSG00000198894 | CIPC | CLOCK interacting pacemaker [Source:HGNC Symbol;Acc:HGNC:20365] | 1.687204 | 2.21E-137 | 1.85E-135 | up |
| ENSG00000185298 | CCDC137 | coiled-coil domain containing 137 [Source:HGNC Symbol;Acc:HGNC:33451] | 1.151638 | 1.14E-136 | 9.49E-135 | up |
| ENSG00000238227 | TMEM250 | transmembrane protein 250 [Source:HGNC Symbol;Acc:HGNC:31009] | 1.213246 | 7.70E-136 | 6.34E-134 | up |
| ENSG00000113494 | PRLR | prolactin receptor [Source:HGNC Symbol;Acc:HGNC:9446] | 2.10175 | 3.90E-135 | 3.19E-133 | up |
| ENSG00000198625 | MDM4 | MDM4, p53 regulator [Source:HGNC Symbol;Acc:HGNC:6974] | 1.275556 | 1.35E-132 | 1.07E-130 | up |
| ENSG00000167397 | VKORC1 | vitamin K epoxide reductase complex subunit 1 [Source:HGNC Symbol;Acc:HGNC:23663] | 1.011039 | 4.46E-130 | 3.49E-128 | up |
| ENSG00000270136 | MINOS1-NBL1 | MINOS1-NBL1 readthrough [Source:HGNC Symbol;Acc:HGNC:48338] | 18.03312 | 6.43E-125 | 4.91E-123 | up |
| ENSG00000013297 | CLDN11 | claudin 11 [Source:HGNC Symbol;Acc:HGNC:8514] | 1.025033 | 4.36E-124 | 3.31E-122 | up |
| ENSG00000171161 | ZNF672 | zinc finger protein 672 [Source:HGNC Symbol;Acc:HGNC:26179] | 1.478927 | 1.70E-121 | 1.27E-119 | up |
| ENSG00000110031 | LPXN | leupaxin [Source:HGNC Symbol;Acc:HGNC:14061] | 1.393731 | 1.12E-118 | 8.25E-117 | up |
| ENSG00000197562 | RAB40C | RAB40C, member RAS oncogene family [Source:HGNC Symbol;Acc:HGNC:18285] | 1.605526 | 1.85E-118 | 1.36E-116 | up |
| ENSG00000099204 | ABLIM1 | actin binding LIM protein 1 [Source:HGNC Symbol;Acc:HGNC:78] | 1.233625 | 4.04E-116 | 2.88E-114 | up |
| ENSG00000126653 | NSRP1 | nuclear speckle splicing regulatory protein 1 [Source:HGNC Symbol;Acc:HGNC:25305] | 1.560494 | 1.56E-109 | 1.04E-107 | up |
| ENSG00000147533 | GOLGA7 | golgin A7 [Source:HGNC Symbol;Acc:HGNC:24876] | 1.601201 | 3.74E-108 | 2.43E-106 | up |
| ENSG00000103042 | SLC38A7 | solute carrier family 38 member 7 [Source:HGNC Symbol;Acc:HGNC:25582] | 1.231974 | 1.48E-107 | 9.58E-106 | up |
| ENSG00000128923 | MINDY2 | MINDY lysine 48 deubiquitinase 2 [Source:HGNC Symbol;Acc:HGNC:26954] | 1.489047 | 3.02E-107 | 1.94E-105 | up |
| ENSG00000033627 | ATP6V0A1 | ATPase H+ transporting V0 subunit a1 [Source:HGNC Symbol;Acc:HGNC:865] | 1.100353 | 1.91E-104 | 1.19E-102 | up |
| ENSG00000128510 | CPA4 | carboxypeptidase A4 [Source:HGNC Symbol;Acc:HGNC:15740] | 2.495593 | 8.35E-102 | 5.06E-100 | up |
| ENSG00000140391 | TSPAN3 | tetraspanin 3 [Source:HGNC Symbol;Acc:HGNC:17752] | 1.72278 | 1.78E-101 | 1.08E-99 | up |
| ENSG00000148516 | ZEB1 | zinc finger E-box binding homeobox 1 [Source:HGNC Symbol;Acc:HGNC:11642] | 1.030248 | 3.32E-96 | 1.89E-94 | up |
| ENSG00000053747 | LAMA3 | laminin subunit alpha 3 [Source:HGNC Symbol;Acc:HGNC:6483] | 1.014233 | 3.40E-94 | 1.87E-92 | up |
| ENSG00000166848 | TERF2IP | TERF2 interacting protein [Source:HGNC Symbol;Acc:HGNC:19246] | 1.391878 | 4.29E-94 | 2.35E-92 | up |
| ENSG00000229124 | VIM-AS1 | VIM antisense RNA 1 [Source:HGNC Symbol;Acc:HGNC:44879] | 1.461491 | 6.69E-89 | 3.52E-87 | up |
| ENSG00000154429 | CCSAP | centriole, cilia and spindle associated protein [Source:HGNC Symbol;Acc:HGNC:29578] | 1.258813 | 2.27E-88 | 1.18E-86 | up |
| ENSG00000204442 | FAM155A | family with sequence similarity 155 member A [Source:HGNC Symbol;Acc:HGNC:33877] | 1.243891 | 3.99E-88 | 2.06E-86 | up |
| ENSG00000214282 | KRT8P14 | keratin 8 pseudogene 14 [Source:HGNC Symbol;Acc:HGNC:33366] | 1.269543 | 1.41E-86 | 7.17E-85 | up |
| ENSG00000185728 | YTHDF3 | YTH N6-methyladenosine RNA binding protein 3 [Source:HGNC Symbol;Acc:HGNC:26465] | 1.174222 | 3.43E-84 | 1.69E-82 | up |
| ENSG00000135823 | STX6 | syntaxin 6 [Source:HGNC Symbol;Acc:HGNC:11441] | 1.541531 | 1.08E-83 | 5.33E-82 | up |
| ENSG00000160285 | LSS | lanosterol synthase [Source:HGNC Symbol;Acc:HGNC:6708] | 1.107967 | 6.11E-82 | 2.92E-80 | up |
| ENSG00000102452 | NALCN | sodium leak channel, non-selective [Source:HGNC Symbol;Acc:HGNC:19082] | 1.050658 | 3.69E-81 | 1.74E-79 | up |
| ENSG00000108433 | GOSR2 | golgi SNAP receptor complex member 2 [Source:HGNC Symbol;Acc:HGNC:4431] | 1.106179 | 1.64E-79 | 7.53E-78 | up |
| ENSG00000171617 | ENC1 | ectodermal-neural cortex 1 [Source:HGNC Symbol;Acc:HGNC:3345] | 1.134206 | 2.34E-75 | 1.00E-73 | up |
| ENSG00000183671 | GPR1 | G protein-coupled receptor 1 [Source:HGNC Symbol;Acc:HGNC:4463] | 1.363973 | 1.81E-74 | 7.71E-73 | up |
| ENSG00000163378 | EOGT | EGF domain specific O-linked N-acetylglucosamine transferase [Source:HGNC Symbol;Acc:HGNC:28526] | 1.394194 | 2.67E-70 | 1.06E-68 | up |
| ENSG00000069702 | TGFBR3 | transforming growth factor beta receptor 3 [Source:HGNC Symbol;Acc:HGNC:11774] | 1.155257 | 1.35E-69 | 5.33E-68 | up |
| ENSG00000157600 | TMEM164 | transmembrane protein 164 [Source:HGNC Symbol;Acc:HGNC:26217] | 1.005678 | 3.19E-69 | 1.25E-67 | up |
| ENSG00000168952 | STXBP6 | syntaxin binding protein 6 [Source:HGNC Symbol;Acc:HGNC:19666] | 1.174619 | 2.93E-67 | 1.12E-65 | up |
| ENSG00000101216 | GMEB2 | glucocorticoid modulatory element binding protein 2 [Source:HGNC Symbol;Acc:HGNC:4371] | 1.016708 | 4.16E-67 | 1.58E-65 | up |
| ENSG00000103066 | PLA2G15 | phospholipase A2 group XV [Source:HGNC Symbol;Acc:HGNC:17163] | 1.30147 | 6.23E-67 | 2.37E-65 | up |
| ENSG00000257046 | AC011604.2 | - | 16.24899 | 1.36E-66 | 5.12E-65 | up |
| ENSG00000204941 | PSG5 | pregnancy specific beta-1-glycoprotein 5 [Source:HGNC Symbol;Acc:HGNC:9522] | 1.489949 | 1.10E-65 | 4.10E-64 | up |
| ENSG00000163751 | CPA3 | carboxypeptidase A3 [Source:HGNC Symbol;Acc:HGNC:2298] | 1.246058 | 3.43E-63 | 1.22E-61 | up |
| ENSG00000153714 | LURAP1L | leucine rich adaptor protein 1 like [Source:HGNC Symbol;Acc:HGNC:31452] | 1.227783 | 5.51E-63 | 1.95E-61 | up |
| ENSG00000154114 | TBCEL | tubulin folding cofactor E like [Source:HGNC Symbol;Acc:HGNC:28115] | 1.312151 | 3.92E-62 | 1.36E-60 | up |
| ENSG00000139292 | LGR5 | leucine rich repeat containing G protein-coupled receptor 5 [Source:HGNC Symbol;Acc:HGNC:4504] | 1.230436 | 1.50E-61 | 5.19E-60 | up |
| ENSG00000224818 | AC096677.3 | - | 18.09797 | 2.37E-60 | 8.05E-59 | up |
| ENSG00000173917 | HOXB2 | homeobox B2 [Source:HGNC Symbol;Acc:HGNC:5113] | 1.082765 | 5.45E-59 | 1.81E-57 | up |
| ENSG00000219545 | UMAD1 | UBAP1-MVB12-associated (UMA) domain containing 1 [Source:HGNC Symbol;Acc:HGNC:48955] | 1.38929 | 6.55E-58 | 2.14E-56 | up |
| ENSG00000139675 | HNRNPA1L2 | heterogeneous nuclear ribonucleoprotein A1-like 2 [Source:HGNC Symbol;Acc:HGNC:27067] | 1.768312 | 2.47E-57 | 7.97E-56 | up |
| ENSG00000189171 | S100A13 | S100 calcium binding protein A13 [Source:HGNC Symbol;Acc:HGNC:10490] | 1.421535 | 3.07E-57 | 9.87E-56 | up |
| ENSG00000145555 | MYO10 | myosin X [Source:HGNC Symbol;Acc:HGNC:7593] | 1.14356 | 3.22E-57 | 1.04E-55 | up |
| ENSG00000253522 | MIR3142HG | MIR3142 host gene [Source:HGNC Symbol;Acc:HGNC:51944] | 16.04014 | 7.38E-57 | 2.36E-55 | up |
| ENSG00000083812 | ZNF324 | zinc finger protein 324 [Source:HGNC Symbol;Acc:HGNC:14096] | 1.659091 | 1.29E-56 | 4.09E-55 | up |
| ENSG00000182551 | ADI1 | acireductone dioxygenase 1 [Source:HGNC Symbol;Acc:HGNC:30576] | 1.148459 | 4.87E-56 | 1.53E-54 | up |
| ENSG00000244332 | AL138759.1 | - | 4.520421 | 5.21E-56 | 1.63E-54 | up |
| ENSG00000164283 | ESM1 | endothelial cell specific molecule 1 [Source:HGNC Symbol;Acc:HGNC:3466] | 1.016567 | 1.17E-55 | 3.67E-54 | up |
| ENSG00000196693 | ZNF33B | zinc finger protein 33B [Source:HGNC Symbol;Acc:HGNC:13097] | 1.40378 | 3.74E-54 | 1.13E-52 | up |
| ENSG00000168542 | COL3A1 | collagen type III alpha 1 chain [Source:HGNC Symbol;Acc:HGNC:2201] | 1.130701 | 6.17E-53 | 1.83E-51 | up |
| ENSG00000196730 | DAPK1 | death associated protein kinase 1 [Source:HGNC Symbol;Acc:HGNC:2674] | 1.165077 | 1.64E-52 | 4.80E-51 | up |
| ENSG00000256514 | AP003419.2 | - | 4.667004 | 3.48E-49 | 9.67E-48 | up |
| ENSG00000225206 | MIR137HG | MIR137 host gene [Source:HGNC Symbol;Acc:HGNC:42871] | 1.318834 | 7.71E-49 | 2.12E-47 | up |
| ENSG00000135362 | PRR5L | proline rich 5 like [Source:HGNC Symbol;Acc:HGNC:25878] | 1.00303 | 1.70E-47 | 4.58E-46 | up |
| ENSG00000114698 | PLSCR4 | phospholipid scramblase 4 [Source:HGNC Symbol;Acc:HGNC:16497] | 1.001692 | 3.11E-47 | 8.28E-46 | up |
| ENSG00000185730 | ZNF696 | zinc finger protein 696 [Source:HGNC Symbol;Acc:HGNC:25872] | 1.115724 | 3.78E-47 | 1.01E-45 | up |
| ENSG00000261796 | ISY1-RAB43 | ISY1-RAB43 readthrough [Source:HGNC Symbol;Acc:HGNC:42969] | 1.236048 | 5.90E-47 | 1.56E-45 | up |
| ENSG00000196653 | ZNF502 | zinc finger protein 502 [Source:HGNC Symbol;Acc:HGNC:23718] | 2.394782 | 2.40E-46 | 6.25E-45 | up |
| ENSG00000206530 | CFAP44 | cilia and flagella associated protein 44 [Source:HGNC Symbol;Acc:HGNC:25631] | 1.013003 | 6.75E-46 | 1.73E-44 | up |
| ENSG00000260804 | LINC01963 | long intergenic non-protein coding RNA 1963 [Source:HGNC Symbol;Acc:HGNC:25283] | 1.051752 | 1.16E-45 | 2.95E-44 | up |
| ENSG00000254122 | PCDHGB7 | protocadherin gamma subfamily B, 7 [Source:HGNC Symbol;Acc:HGNC:8714] | 1.177425 | 1.94E-45 | 4.88E-44 | up |
| ENSG00000187837 | HIST1H1C | histone cluster 1 H1 family member c [Source:HGNC Symbol;Acc:HGNC:4716] | 1.444 | 5.96E-44 | 1.45E-42 | up |
| ENSG00000260604 | AL590004.4 | - | 1.012569 | 6.90E-44 | 1.68E-42 | up |
| ENSG00000065361 | ERBB3 | erb-b2 receptor tyrosine kinase 3 [Source:HGNC Symbol;Acc:HGNC:3431] | 1.038301 | 6.46E-43 | 1.53E-41 | up |
| ENSG00000093100 | AC016026.1 | - | 1.086875 | 1.01E-40 | 2.25E-39 | up |
| ENSG00000222009 | BTBD19 | BTB domain containing 19 [Source:HGNC Symbol;Acc:HGNC:27145] | 1.937511 | 1.53E-39 | 3.32E-38 | up |
| ENSG00000231924 | PSG1 | pregnancy specific beta-1-glycoprotein 1 [Source:HGNC Symbol;Acc:HGNC:9514] | 1.500914 | 3.01E-38 | 6.34E-37 | up |
| ENSG00000248167 | TRIM39-RPP21 | TRIM39-RPP21 readthrough [Source:HGNC Symbol;Acc:HGNC:38845] | 15.71071 | 4.96E-37 | 1.02E-35 | up |
| ENSG00000169432 | SCN9A | sodium voltage-gated channel alpha subunit 9 [Source:HGNC Symbol;Acc:HGNC:10597] | 1.128857 | 4.26E-36 | 8.55E-35 | up |
| ENSG00000152234 | ATP5A1 | ATP synthase, H+ transporting, mitochondrial F1 complex, alpha subunit 1, cardiac muscle [Source:HGNC Symbol;Acc:HGNC:823] | 1.008257 | 8.98E-36 | 1.78E-34 | up |
| ENSG00000118894 | EEF2KMT | eukaryotic elongation factor 2 lysine methyltransferase [Source:HGNC Symbol;Acc:HGNC:32221] | 1.016508 | 2.61E-35 | 5.13E-34 | up |
| ENSG00000157404 | KIT | KIT proto-oncogene receptor tyrosine kinase [Source:HGNC Symbol;Acc:HGNC:6342] | 1.116342 | 3.02E-35 | 5.92E-34 | up |
| ENSG00000157870 | FAM213B | family with sequence similarity 213 member B [Source:HGNC Symbol;Acc:HGNC:28390] | 1.105932 | 1.78E-34 | 3.42E-33 | up |
| ENSG00000100478 | AP4S1 | adaptor related protein complex 4 sigma 1 subunit [Source:HGNC Symbol;Acc:HGNC:575] | 1.057991 | 2.03E-33 | 3.77E-32 | up |
| ENSG00000125962 | ARMCX5 | armadillo repeat containing, X-linked 5 [Source:HGNC Symbol;Acc:HGNC:25772] | 1.530372 | 1.86E-32 | 3.37E-31 | up |
| ENSG00000188785 | ZNF548 | zinc finger protein 548 [Source:HGNC Symbol;Acc:HGNC:26561] | 1.214374 | 3.10E-32 | 5.59E-31 | up |
| ENSG00000186272 | ZNF17 | zinc finger protein 17 [Source:HGNC Symbol;Acc:HGNC:12958] | 1.402068 | 3.66E-32 | 6.57E-31 | up |
| ENSG00000138867 | GUCD1 | guanylyl cyclase domain containing 1 [Source:HGNC Symbol;Acc:HGNC:14237] | 1.071439 | 5.64E-31 | 9.78E-30 | up |
| ENSG00000179855 | GIPC3 | GIPC PDZ domain containing family member 3 [Source:HGNC Symbol;Acc:HGNC:18183] | 1.073986 | 9.44E-31 | 1.63E-29 | up |
| ENSG00000164761 | TNFRSF11B | TNF receptor superfamily member 11b [Source:HGNC Symbol;Acc:HGNC:11909] | 1.626215 | 1.22E-30 | 2.10E-29 | up |
| ENSG00000104343 | UBE2W | ubiquitin conjugating enzyme E2 W [Source:HGNC Symbol;Acc:HGNC:25616] | 1.187153 | 9.00E-30 | 1.52E-28 | up |
| ENSG00000167371 | PRRT2 | proline rich transmembrane protein 2 [Source:HGNC Symbol;Acc:HGNC:30500] | 1.775921 | 2.27E-29 | 3.75E-28 | up |
| ENSG00000128606 | LRRC17 | leucine rich repeat containing 17 [Source:HGNC Symbol;Acc:HGNC:16895] | 1.591612 | 3.09E-29 | 5.07E-28 | up |
| ENSG00000110881 | ASIC1 | acid sensing ion channel subunit 1 [Source:HGNC Symbol;Acc:HGNC:100] | 1.138023 | 9.03E-29 | 1.45E-27 | up |
| ENSG00000273038 | AL365203.3 | - | 1.239415 | 1.83E-27 | 2.79E-26 | up |
| ENSG00000170848 | PSG6 | pregnancy specific beta-1-glycoprotein 6 [Source:HGNC Symbol;Acc:HGNC:9523] | 1.68652 | 6.56E-27 | 9.82E-26 | up |
| ENSG00000197635 | DPP4 | dipeptidyl peptidase 4 [Source:HGNC Symbol;Acc:HGNC:3009] | 1.190527 | 4.31E-26 | 6.29E-25 | up |
| ENSG00000123843 | C4BPB | complement component 4 binding protein beta [Source:HGNC Symbol;Acc:HGNC:1328] | 3.539784 | 5.92E-25 | 8.27E-24 | up |
| ENSG00000257065 | AL049844.1 | - | 3.434978 | 2.46E-24 | 3.37E-23 | up |
| ENSG00000153485 | TMEM251 | transmembrane protein 251 [Source:HGNC Symbol;Acc:HGNC:20218] | 1.406779 | 4.44E-24 | 6.02E-23 | up |
| ENSG00000230836 | LINC01293 | long intergenic non-protein coding RNA 1293 [Source:HGNC Symbol;Acc:HGNC:50362] | 1.110132 | 4.91E-24 | 6.64E-23 | up |
| ENSG00000094841 | UPRT | uracil phosphoribosyltransferase homolog [Source:HGNC Symbol;Acc:HGNC:28334] | 1.102953 | 2.04E-23 | 2.69E-22 | up |
| ENSG00000125843 | AP5S1 | adaptor related protein complex 5 sigma 1 subunit [Source:HGNC Symbol;Acc:HGNC:15875] | 1.332867 | 5.62E-23 | 7.27E-22 | up |
| ENSG00000282827 | AC134772.2 | - | 1.144815 | 6.49E-23 | 8.37E-22 | up |
| ENSG00000129422 | MTUS1 | microtubule associated scaffold protein 1 [Source:HGNC Symbol;Acc:HGNC:29789] | 1.585917 | 1.47E-22 | 1.86E-21 | up |
| ENSG00000151090 | THRB | thyroid hormone receptor beta [Source:HGNC Symbol;Acc:HGNC:11799] | 1.05729 | 3.00E-22 | 3.74E-21 | up |
| ENSG00000231672 | DIRC3 | disrupted in renal carcinoma 3 [Source:HGNC Symbol;Acc:HGNC:17805] | 1.077293 | 8.01E-22 | 9.75E-21 | up |
| ENSG00000204792 | LINC01291 | long intergenic non-protein coding RNA 1291 [Source:HGNC Symbol;Acc:HGNC:50358] | 1.265093 | 3.91E-21 | 4.64E-20 | up |
| ENSG00000224078 | SNHG14 | small nucleolar RNA host gene 14 [Source:HGNC Symbol;Acc:HGNC:37462] | 1.096435 | 1.05E-19 | 1.17E-18 | up |
| ENSG00000272695 | GAS6-AS2 | GAS6 antisense RNA 2 (head to head) [Source:HGNC Symbol;Acc:HGNC:43694] | 1.23269 | 1.21E-19 | 1.34E-18 | up |
| ENSG00000163884 | KLF15 | Kruppel like factor 15 [Source:HGNC Symbol;Acc:HGNC:14536] | 1.147353 | 2.99E-18 | 3.12E-17 | up |
| ENSG00000268439 | EMG1 | EMG1, N1-specific pseudouridine methyltransferase [Source:HGNC Symbol;Acc:HGNC:16912] | 1.98116 | 6.29E-18 | 6.49E-17 | up |
| ENSG00000267827 | AC011468.2 | - | 4.927169 | 1.72E-16 | 1.65E-15 | up |
| ENSG00000182261 | NLRP10 | NLR family pyrin domain containing 10 [Source:HGNC Symbol;Acc:HGNC:21464] | 2.262549 | 1.80E-16 | 1.73E-15 | up |
| ENSG00000109270 | LAMTOR3 | late endosomal/lysosomal adaptor, MAPK and MTOR activator 3 [Source:HGNC Symbol;Acc:HGNC:15606] | 1.017365 | 5.97E-16 | 5.56E-15 | up |
| ENSG00000164483 | SAMD3 | sterile alpha motif domain containing 3 [Source:HGNC Symbol;Acc:HGNC:21574] | 1.094658 | 2.84E-15 | 2.55E-14 | up |
| ENSG00000237523 | LINC00857 | long intergenic non-protein coding RNA 857 [Source:HGNC Symbol;Acc:HGNC:45114] | 1.250685 | 3.03E-15 | 2.71E-14 | up |
| ENSG00000182568 | SATB1 | SATB homeobox 1 [Source:HGNC Symbol;Acc:HGNC:10541] | 1.113401 | 1.35E-13 | 1.09E-12 | up |
| ENSG00000232453 | AC105277.1 | - | 1.39121 | 2.23E-13 | 1.77E-12 | up |
| ENSG00000213846 | AC098614.1 | - | 1.649779 | 2.51E-13 | 1.99E-12 | up |
| ENSG00000249279 | LINC02057 | long intergenic non-protein coding RNA 2057 [Source:HGNC Symbol;Acc:HGNC:52900] | 1.04859 | 7.45E-13 | 5.74E-12 | up |
| ENSG00000269416 | LINC01224 | long intergenic non-protein coding RNA 1224 [Source:HGNC Symbol;Acc:HGNC:49676] | 1.006795 | 1.01E-12 | 7.73E-12 | up |
| ENSG00000234106 | AP004242.1 | - | 5.068287 | 3.22E-12 | 2.38E-11 | up |
| ENSG00000134548 | SPX | spexin hormone [Source:HGNC Symbol;Acc:HGNC:28139] | 1.16495 | 6.88E-12 | 4.95E-11 | up |
| ENSG00000228343 | AC115618.2 | - | 1.104029 | 1.04E-11 | 7.39E-11 | up |
| ENSG00000155974 | GRIP1 | glutamate receptor interacting protein 1 [Source:HGNC Symbol;Acc:HGNC:18708] | 1.973879 | 2.42E-11 | 1.68E-10 | up |
| ENSG00000180573 | HIST1H2AC | histone cluster 1 H2A family member c [Source:HGNC Symbol;Acc:HGNC:4733] | 1.086145 | 4.96E-11 | 3.38E-10 | up |
| ENSG00000197261 | C6orf141 | chromosome 6 open reading frame 141 [Source:HGNC Symbol;Acc:HGNC:21351] | 1.255381 | 5.12E-11 | 3.49E-10 | up |
| ENSG00000137878 | GCOM1 | GRINL1A complex locus 1 [Source:HGNC Symbol;Acc:HGNC:26424] | 1.152928 | 6.72E-11 | 4.54E-10 | up |
| ENSG00000279535 | AP003476.1 | - | 1.522962 | 2.63E-10 | 1.71E-09 | up |
| ENSG00000284202 | MIR137 | microRNA 137 [Source:HGNC Symbol;Acc:HGNC:31523] | 6.447385 | 4.24E-10 | 2.70E-09 | up |
| ENSG00000239407 | Z68871.1 | - | 1.065776 | 5.12E-10 | 3.25E-09 | up |
| ENSG00000156510 | HKDC1 | hexokinase domain containing 1 [Source:HGNC Symbol;Acc:HGNC:23302] | 1.187035 | 5.98E-10 | 3.77E-09 | up |
| ENSG00000225205 | AC078883.1 | - | 1.441819 | 8.42E-10 | 5.24E-09 | up |
| ENSG00000151576 | QTRT2 | queuine tRNA-ribosyltransferase accessory subunit 2 [Source:HGNC Symbol;Acc:HGNC:25771] | 1.098196 | 9.79E-10 | 6.06E-09 | up |
| ENSG00000230982 | DSTNP1 | destrin, actin depolymerizing factor pseudogene 1 [Source:HGNC Symbol;Acc:HGNC:23769] | 1.334175 | 1.82E-09 | 1.10E-08 | up |
| ENSG00000154040 | CABYR | calcium binding tyrosine phosphorylation regulated [Source:HGNC Symbol;Acc:HGNC:15569] | 1.009415 | 4.26E-09 | 2.49E-08 | up |
| ENSG00000221852 | KRTAP1-5 | keratin associated protein 1-5 [Source:HGNC Symbol;Acc:HGNC:16777] | 1.186613 | 4.67E-09 | 2.72E-08 | up |
| ENSG00000226396 | AL031727.1 | - | 2.413256 | 1.66E-08 | 9.25E-08 | up |
| ENSG00000267523 | AC008735.2 | - | 1.132463 | 4.02E-08 | 2.17E-07 | up |
| ENSG00000137463 | MGARP | mitochondria localized glutamic acid rich protein [Source:HGNC Symbol;Acc:HGNC:29969] | 1.077224 | 1.23E-07 | 6.29E-07 | up |
| ENSG00000258667 | HIF1A-AS2 | HIF1A antisense RNA 2 [Source:HGNC Symbol;Acc:HGNC:43015] | 1.14621 | 1.41E-07 | 7.17E-07 | up |
| ENSG00000144785 | AC073896.1 | - | 1.30287 | 1.68E-07 | 8.48E-07 | up |
| ENSG00000229563 | LINC01204 | long intergenic non-protein coding RNA 1204 [Source:HGNC Symbol;Acc:HGNC:49635] | 1.029267 | 2.15E-07 | 1.07E-06 | up |
| ENSG00000248124 | RRN3P1 | RRN3 homolog, RNA polymerase I transcription factor pseudogene 1 [Source:HGNC Symbol;Acc:HGNC:30548] | 1.03697 | 3.65E-07 | 1.79E-06 | up |
| ENSG00000135709 | KIAA0513 | KIAA0513 [Source:HGNC Symbol;Acc:HGNC:29058] | 1.156589 | 7.70E-07 | 3.65E-06 | up |
| ENSG00000248927 | AC114284.1 | - | 1.059502 | 1.10E-06 | 5.13E-06 | up |
| ENSG00000088727 | KIF9 | kinesin family member 9 [Source:HGNC Symbol;Acc:HGNC:16666] | 1.766859 | 1.92E-06 | 8.73E-06 | up |
| ENSG00000121769 | FABP3 | fatty acid binding protein 3 [Source:HGNC Symbol;Acc:HGNC:3557] | 1.294066 | 2.45E-06 | 1.10E-05 | up |
| ENSG00000249592 | AC139887.2 | - | 1.067298 | 1.01E-05 | 4.20E-05 | up |
| ENSG00000213139 | CRYGS | crystallin gamma S [Source:HGNC Symbol;Acc:HGNC:2417] | 1.039854 | 1.33E-05 | 5.48E-05 | up |
| ENSG00000270084 | GAS5-AS1 | GAS5 antisense RNA 1 [Source:HGNC Symbol;Acc:HGNC:44119] | 1.149681 | 2.64E-05 | 0.000105 | up |
| ENSG00000234373 | SNX18P7 | sorting nexin 18 pseudogene 7 [Source:HGNC Symbol;Acc:HGNC:39615] | 1.14941 | 5.80E-05 | 0.00022 | up |
| ENSG00000206712 | RNU6-26P | RNA, U6 small nuclear 26, pseudogene [Source:HGNC Symbol;Acc:HGNC:34252] | 1.975575 | 0.000123 | 0.000444 | up |
| ENSG00000267547 | AC060766.4 | - | 1.101788 | 0.000133 | 0.000478 | up |
| ENSG00000262663 | AC087222.1 | - | 1.10457 | 0.000156 | 0.000556 | up |
| ENSG00000275996 | SNORD27 | small nucleolar RNA, C/D box 27 [Source:HGNC Symbol;Acc:HGNC:10149] | 5.10453 | 0.000194 | 0.000682 | up |
| ENSG00000251129 | AC112251.1 | - | 1.02996 | 0.000248 | 0.000857 | up |
| ENSG00000154589 | LY96 | lymphocyte antigen 96 [Source:HGNC Symbol;Acc:HGNC:17156] | 1.043649 | 0.000293 | 0.001001 | up |
| ENSG00000214827 | MTCP1 | mature T-cell proliferation 1 [Source:HGNC Symbol;Acc:HGNC:7423] | 1.099456 | 0.000307 | 0.001044 | up |
| ENSG00000230953 | AC099677.1 | - | 1.227217 | 0.003397 | 0.009547 | up |
| ENSG00000144285 | SCN1A | sodium voltage-gated channel alpha subunit 1 [Source:HGNC Symbol;Acc:HGNC:10585] | 1.544556 | 0.003859 | 0.010742 | up |
| ENSG00000232901 | CYCSP10 | cytochrome c, somatic pseudogene 10 [Source:HGNC Symbol;Acc:HGNC:24384] | 1.249358 | 0.005461 | 0.014656 | up |
| ENSG00000275072 | SNORD50B | small nucleolar RNA, C/D box 50B [Source:HGNC Symbol;Acc:HGNC:32722] | 4.637854 | 0.007958 | 0.020555 | up |
| ENSG00000136213 | CHST12 | carbohydrate sulfotransferase 12 [Source:HGNC Symbol;Acc:HGNC:17423] | 1.711281 | 0.008073 | 0.020824 | up |
| ENSG00000210112 | MT-TM | mitochondrially encoded tRNA methionine [Source:HGNC Symbol;Acc:HGNC:7492] | 1.276422 | 0.009293 | 0.023705 | up |
| ENSG00000207118 | SNORD14D | small nucleolar RNA, C/D box 14D [Source:HGNC Symbol;Acc:HGNC:30353] | 3.064806 | 0.010348 | 0.026004 | up |
| ENSG00000229604 | MTATP8P2 | mitochondrially encoded ATP synthase 8 pseudogene 2 [Source:HGNC Symbol;Acc:HGNC:44572] | 1.17562 | 0.013433 | 0.032711 | up |
| ENSG00000199332 | Y_RNA | Y RNA [Source:RFAM;Acc:RF00019] | 1.694475 | 0.018077 | 0.042749 | up |
