# Supplementary material for: Identification of downstream targets and signaling pathways of long non-coding RNA NR_002794 in human trophoblast cells
Source: Bioengineered. 2021 Sep 13;12(1):6617–28. doi: 10.1080/21655979.2021.1974808 (PMC8806843; doi:10.1080/21655979.2021.1974808)
Supplement: Supplemental Material [file KBIE_A_1974808_SM0255.zip › supplementary/supplementary Table 3.docx]

| **Common differentially expressed genes in both KD versus NC group and OE versus NC group** | | |
| --- | --- | --- |
| ENSG00000261915 | AC026954.2 | |
| ENSG00000188223 | AD000671.1 | |
| ENSG00000265590 | AP000275.2 | |
| ENSG00000258529 | AP001781.3 | |
| ENSG00000187689 | AMTN |  |
| ENSG00000264346 | SNORA77B | |
| ENSG00000275121 | AC211486.4 | |
| ENSG00000227706 | AL713998.1 | |
| ENSG00000281808 | SNORA17 |  |
| ENSG00000278334 | HOXA11-AS1_2 | |
| ENSG00000281453 | TGFB2-OT1 | |
| ENSG00000167244 | IGF2 |  |
| ENSG00000257949 | TEN1 |  |
| ENSG00000214282 | KRT8P14 |  |
| ENSG00000248871 | TNFSF12-TNFSF13 | |
| ENSG00000207725 | MIR222 |  |
| ENSG00000092096 | SLC22A17 |  |
| ENSG00000072818 | ACAP1 |  |
| ENSG00000277945 | AC107308.1 | |
| ENSG00000267261 | AC099811.2 | |
| ENSG00000172466 | ZNF24 |  |
| ENSG00000284194 | SCO2 |  |
| ENSG00000185022 | MAFF |  |
| ENSG00000198899 | MT-ATP6 |  |
| ENSG00000126709 | IFI6 |  |
| ENSG00000107984 | DKK1 |  |
| ENSG00000164362 | TERT |  |
| ENSG00000173875 | ZNF791 |  |
| ENSG00000229124 | VIM-AS1 |  |
| ENSG00000139292 | LGR5 |  |
| ENSG00000153291 | SLC25A27 |  |
| ENSG00000187210 | GCNT1 |  |
| ENSG00000063322 | MED29 |  |
| ENSG00000176624 | MEX3C |  |
| ENSG00000258653 | AC005520.1 | |
| ENSG00000164284 | GRPEL2 |  |
| ENSG00000204604 | ZNF468 |  |
| ENSG00000229563 | LINC01204 | |
| ENSG00000222365 | SNORD12B | |
| ENSG00000258667 | HIF1A-AS2 | |
| ENSG00000255526 | NEDD8-MDP1 | |
| ENSG00000198040 | ZNF84 |  |
| ENSG00000197965 | MPZL1 |  |
| ENSG00000093100 | AC016026.1 | |
| ENSG00000161671 | EMC10 |  |
| ENSG00000106484 | MEST |  |
| ENSG00000258232 | AC125611.3 | |
| ENSG00000163359 | COL6A3 |  |
| ENSG00000172250 | SERHL |  |
| ENSG00000273291 | AC092042.3 | |
| ENSG00000267523 | AC008735.2 | |
| ENSG00000115919 | KYNU |  |
| ENSG00000064300 | NGFR |  |
| ENSG00000196337 | CGB7 |  |
| ENSG00000163017 | ACTG2 |  |
| ENSG00000272275 | AC092687.3 | |
| ENSG00000173402 | DAG1 |  |
| ENSG00000226145 | KRT16P6 |  |
| ENSG00000269028 | MTRNR2L12 | |
| ENSG00000161847 | RAVER1 |  |
| ENSG00000198930 | CSAG1 |  |
| ENSG00000209480 | SNORD83B | |
| ENSG00000168301 | KCTD6 |  |
| ENSG00000124208 | TMEM189-UBE2V1 | |
| ENSG00000119729 | RHOQ |  |
| ENSG00000177427 | MIEF2 |  |
| ENSG00000154553 | PDLIM3 |  |
| ENSG00000114251 | WNT5A |  |
| ENSG00000156671 | SAMD8 |  |
| ENSG00000267520 | AC010733.2 | |
| ENSG00000231503 | PTMAP4 |  |
| ENSG00000213780 | GTF2H4 |  |
| ENSG00000240184 | PCDHGC3 |  |
| ENSG00000198860 | TSEN15 |  |
| ENSG00000271853 | AL162258.1 | |
| ENSG00000125968 | ID1 |  |
| ENSG00000227615 | AP001324.1 | |
| ENSG00000267598 | AC011446.2 | |
| ENSG00000116489 | CAPZA1 |  |
| ENSG00000011590 | ZBTB32 |  |
| ENSG00000186866 | POFUT2 |  |
| ENSG00000239264 | TXNDC5 |  |
| ENSG00000144677 | CTDSPL |  |
| ENSG00000260615 | RPL23AP97 | |
| ENSG00000187134 | AKR1C1 |  |
| ENSG00000154710 | RABGEF1 |  |
| ENSG00000276085 | CCL3L1 |  |
| ENSG00000113532 | ST8SIA4 |  |
| ENSG00000170345 | FOS |  |
| ENSG00000188732 | FAM221A |  |
| ENSG00000260238 | PMF1-BGLAP | |
| ENSG00000205084 | TMEM231 |  |
| ENSG00000248124 | RRN3P1 |  |
| ENSG00000132561 | MATN2 |  |
| ENSG00000011677 | GABRA3 |  |
| ENSG00000134470 | IL15RA |  |
| ENSG00000125841 | NRSN2 |  |
| ENSG00000198947 | DMD |  |
| ENSG00000222009 | BTBD19 |  |
| ENSG00000147533 | GOLGA7 |  |
| ENSG00000110881 | ASIC1 |  |
| ENSG00000276070 | CCL4L2 |  |
| ENSG00000114315 | HES1 |  |
| ENSG00000173917 | HOXB2 |  |
| ENSG00000163378 | EOGT |  |
| ENSG00000267680 | ZNF224 |  |
| ENSG00000157600 | TMEM164 |  |
| ENSG00000219545 | UMAD1 |  |
| ENSG00000213846 | AC098614.1 | |
| ENSG00000167397 | VKORC1 |  |
| ENSG00000183291 | SELENOF |  |
| ENSG00000189171 | S100A13 |  |
| ENSG00000214827 | MTCP1 |  |
| ENSG00000134590 | RTL8C |  |
| ENSG00000066056 | TIE1 |  |
| ENSG00000154429 | CCSAP |  |
| ENSG00000139675 | HNRNPA1L2 | |
| ENSG00000137878 | GCOM1 |  |
| ENSG00000101216 | GMEB2 |  |
| ENSG00000008517 | IL32 |  |
| ENSG00000128923 | MINDY2 |  |
| ENSG00000198894 | CIPC |  |
| ENSG00000187446 | CHP1 |  |
| ENSG00000146674 | IGFBP3 |  |
| ENSG00000102934 | PLLP |  |
| ENSG00000154589 | LY96 |  |
| ENSG00000182158 | CREB3L2 |  |
| ENSG00000154114 | TBCEL |  |
| ENSG00000135823 | STX6 |  |
| ENSG00000170498 | KISS1 |  |
| ENSG00000103066 | PLA2G15 |  |
| ENSG00000182551 | ADI1 |  |
| ENSG00000134321 | RSAD2 |  |
| ENSG00000140391 | TSPAN3 |  |
| ENSG00000253873 | PCDHGA11 | |
| ENSG00000104765 | BNIP3L |  |
| ENSG00000166913 | YWHAB |  |
| ENSG00000136732 | GYPC |  |
| ENSG00000238227 | TMEM250 |  |
| ENSG00000283515 | AC020915.6 | |
| ENSG00000197757 | HOXC6 |  |
| ENSG00000196693 | ZNF33B |  |
| ENSG00000143970 | ASXL2 |  |
| ENSG00000250748 | AC025419.1 | |
| ENSG00000206053 | JPT2 |  |
| ENSG00000166848 | TERF2IP |  |
| ENSG00000134548 | SPX |  |
| ENSG00000197562 | RAB40C |  |
| ENSG00000111348 | ARHGDIB |  |
| ENSG00000127314 | RAP1B |  |
| ENSG00000125962 | ARMCX5 |  |
| ENSG00000175274 | TP53I11 |  |
| ENSG00000207118 | SNORD14D | |
| ENSG00000284461 | RABGEF1 |  |
| ENSG00000270181 | BIVM-ERCC5 | |
| ENSG00000284202 | MIR137 |  |
| ENSG00000275996 | SNORD27 |  |
| ENSG00000248167 | TRIM39-RPP21 | |
| ENSG00000260772 | AC012321.1 | |
