# Supplementary material for: Identification of downstream targets and signaling pathways of long non-coding RNA NR_002794 in human trophoblast cells
Source: Bioengineered. 2021 Sep 13;12(1):6617–28. doi: 10.1080/21655979.2021.1974808 (PMC8806843; doi:10.1080/21655979.2021.1974808)
Supplement: Supplemental Material [file KBIE_A_1974808_SM0255.zip › supplementary/supplementary Table 4.docx]

| ##Databases: KEGG PATHWAY |  |  |  |  |  |  |  |  |
| --- | --- | --- | --- | --- | --- | --- | --- | --- |
| ##Statistical test method: hypergeometric test / Fisher's exact test | | | | |  |  |  |  |
| ##FDR correction method: Benjamini and Hochberg | |  |  |  |  |  |  |  |
|  |  |  |  |  |  |  |  |  |
| #Term | Database | ID | Input number | Background number | P-Value | Corrected P-Value | Input | Hyperlink |
| Toll-like receptor signaling pathway | KEGG PATHWAY | hsa04620 | 4 | 104 | 0.00048 | 0.065741 | LY96\|CCL4L2\|CCL3L1\|FOS | http://www.genome.jp/kegg-bin/show_pathway?hsa04620/hsa:9560%09red/hsa:2353%09red/hsa:23643%09red/hsa:6349%09red |
| Other types of O-glycan biosynthesis | KEGG PATHWAY | hsa00514 | 2 | 22 | 0.00289 | 0.114303 | POFUT2\|EOGT | http://www.genome.jp/kegg-bin/show_pathway?hsa00514/hsa:285203%09red/hsa:23275%09red |
| Salmonella infection | KEGG PATHWAY | hsa05132 | 3 | 83 | 0.003011 | 0.114303 | CCL4L2\|CCL3L1\|FOS | http://www.genome.jp/kegg-bin/show_pathway?hsa05132/hsa:9560%09red/hsa:2353%09red/hsa:6349%09red |
| Cytokine-cytokine receptor interaction | KEGG PATHWAY | hsa04060 | 5 | 294 | 0.003337 | 0.114303 | IL15RA\|CCL4L2\|IL32\|NGFR\|CCL3L1 | http://www.genome.jp/kegg-bin/show_pathway?hsa04060/hsa:3601%09red/hsa:4804%09red/hsa:9560%09red/hsa:6349%09red/hsa:9235%09red |
| Human T-cell leukemia virus 1 infection | KEGG PATHWAY | hsa05166 | 4 | 219 | 0.006701 | 0.155919 | CREB3L2\|TERT\|IL15RA\|FOS | http://www.genome.jp/kegg-bin/show_pathway?hsa05166/hsa:3601%09red/hsa:64764%09red/hsa:2353%09red/hsa:7015%09red |
| PI3K-Akt signaling pathway | KEGG PATHWAY | hsa04151 | 5 | 354 | 0.007158 | 0.155919 | CREB3L2\|IGF2\|COL6A3\|YWHAB\|NGFR | http://www.genome.jp/kegg-bin/show_pathway?hsa04151/hsa:64764%09red/hsa:7529%09red/hsa:4804%09red/hsa:3481%09red/hsa:1293%09red |
| Neurotrophin signaling pathway | KEGG PATHWAY | hsa04722 | 3 | 119 | 0.007967 | 0.155919 | ARHGDIB\|NGFR\|RAP1B | http://www.genome.jp/kegg-bin/show_pathway?hsa04722/hsa:5908%09red/hsa:397%09red/hsa:4804%09red |
| Vasopressin-regulated water reabsorption | KEGG PATHWAY | hsa04962 | 2 | 44 | 0.010332 | 0.166556 | CREB3L2\|ARHGDIB | http://www.genome.jp/kegg-bin/show_pathway?hsa04962/hsa:64764%09red/hsa:397%09red |
| Nucleotide excision repair | KEGG PATHWAY | hsa03420 | 2 | 47 | 0.011663 | 0.166556 | BIVM-ERCC5\|GTF2H4 | http://www.genome.jp/kegg-bin/show_pathway?hsa03420/hsa:100533467%09red/hsa:2968%09red |
| Hippo signaling pathway | KEGG PATHWAY | hsa04390 | 3 | 154 | 0.015702 | 0.166556 | YWHAB\|WNT5A\|ID1 | http://www.genome.jp/kegg-bin/show_pathway?hsa04390/hsa:3397%09red/hsa:7529%09red/hsa:7474%09red |
| Cushing syndrome | KEGG PATHWAY | hsa04934 | 3 | 155 | 0.015968 | 0.166556 | CREB3L2\|WNT5A\|RAP1B | http://www.genome.jp/kegg-bin/show_pathway?hsa04934/hsa:5908%09red/hsa:64764%09red/hsa:7474%09red |
| Wnt signaling pathway | KEGG PATHWAY | hsa04310 | 3 | 160 | 0.017335 | 0.166556 | LGR5\|WNT5A\|DKK1 | http://www.genome.jp/kegg-bin/show_pathway?hsa04310/hsa:8549%09red/hsa:7474%09red/hsa:22943%09red |
| MAPK signaling pathway | KEGG PATHWAY | hsa04010 | 4 | 295 | 0.018007 | 0.166556 | IGF2\|RAP1B\|NGFR\|FOS | http://www.genome.jp/kegg-bin/show_pathway?hsa04010/hsa:5908%09red/hsa:4804%09red/hsa:2353%09red/hsa:3481%09red |
| Hepatitis B | KEGG PATHWAY | hsa05161 | 3 | 163 | 0.018185 | 0.166556 | CREB3L2\|YWHAB\|FOS | http://www.genome.jp/kegg-bin/show_pathway?hsa05161/hsa:64764%09red/hsa:7529%09red/hsa:2353%09red |
| Viral myocarditis | KEGG PATHWAY | hsa05416 | 2 | 60 | 0.018236 | 0.166556 | DMD\|DAG1 | http://www.genome.jp/kegg-bin/show_pathway?hsa05416/hsa:1605%09red/hsa:1756%09red |
| Hepatocellular carcinoma | KEGG PATHWAY | hsa05225 | 3 | 168 | 0.019654 | 0.168287 | IGF2\|WNT5A\|TERT | http://www.genome.jp/kegg-bin/show_pathway?hsa05225/hsa:7474%09red/hsa:7015%09red/hsa:3481%09red |
| Amphetamine addiction | KEGG PATHWAY | hsa05031 | 2 | 68 | 0.022892 | 0.17658 | CREB3L2\|FOS | http://www.genome.jp/kegg-bin/show_pathway?hsa05031/hsa:64764%09red/hsa:2353%09red |
| Herpes simplex virus 1 infection | KEGG PATHWAY | hsa05168 | 5 | 492 | 0.025638 | 0.17658 | ZNF224\|ZNF468\|ZNF33B\|ZNF791\|ZNF84 | http://www.genome.jp/kegg-bin/show_pathway?hsa05168/hsa:7767%09red/hsa:388561%09red/hsa:7582%09red/hsa:7637%09red/hsa:163049%09red |
| Human papillomavirus infection | KEGG PATHWAY | hsa05165 | 4 | 330 | 0.025739 | 0.17658 | CREB3L2\|COL6A3\|TERT\|WNT5A | http://www.genome.jp/kegg-bin/show_pathway?hsa05165/hsa:64764%09red/hsa:7474%09red/hsa:7015%09red/hsa:1293%09red |
| Chemokine signaling pathway | KEGG PATHWAY | hsa04062 | 3 | 190 | 0.026873 | 0.17658 | CCL4L2\|RAP1B\|CCL3L1 | http://www.genome.jp/kegg-bin/show_pathway?hsa04062/hsa:5908%09red/hsa:9560%09red/hsa:6349%09red |
| Pertussis | KEGG PATHWAY | hsa05133 | 2 | 76 | 0.027981 | 0.17658 | LY96\|FOS | http://www.genome.jp/kegg-bin/show_pathway?hsa05133/hsa:2353%09red/hsa:23643%09red |
| Arrhythmogenic right ventricular cardiomyopathy (ARVC) | KEGG PATHWAY | hsa05412 | 2 | 77 | 0.028647 | 0.17658 | DMD\|DAG1 | http://www.genome.jp/kegg-bin/show_pathway?hsa05412/hsa:1605%09red/hsa:1756%09red |
| Viral carcinogenesis | KEGG PATHWAY | hsa05203 | 3 | 201 | 0.030945 | 0.17658 | CREB3L2\|YWHAB\|GTF2H4 | http://www.genome.jp/kegg-bin/show_pathway?hsa05203/hsa:64764%09red/hsa:7529%09red/hsa:2968%09red |
| Pathways in cancer | KEGG PATHWAY | hsa05200 | 5 | 530 | 0.033648 | 0.17658 | IGF2\|WNT5A\|TERT\|IL15RA\|FOS | http://www.genome.jp/kegg-bin/show_pathway?hsa05200/hsa:3601%09red/hsa:7474%09red/hsa:2353%09red/hsa:7015%09red/hsa:3481%09red |
| Rap1 signaling pathway | KEGG PATHWAY | hsa04015 | 3 | 210 | 0.034504 | 0.17658 | ID1\|NGFR\|RAP1B | http://www.genome.jp/kegg-bin/show_pathway?hsa04015/hsa:5908%09red/hsa:3397%09red/hsa:4804%09red |
| ECM-receptor interaction | KEGG PATHWAY | hsa04512 | 2 | 86 | 0.034913 | 0.17658 | COL6A3\|DAG1 | http://www.genome.jp/kegg-bin/show_pathway?hsa04512/hsa:1605%09red/hsa:1293%09red |
| cAMP signaling pathway | KEGG PATHWAY | hsa04024 | 3 | 214 | 0.036151 | 0.17658 | CREB3L2\|RAP1B\|FOS | http://www.genome.jp/kegg-bin/show_pathway?hsa04024/hsa:5908%09red/hsa:64764%09red/hsa:2353%09red |
| Hypertrophic cardiomyopathy (HCM) | KEGG PATHWAY | hsa05410 | 2 | 90 | 0.037853 | 0.17658 | DMD\|DAG1 | http://www.genome.jp/kegg-bin/show_pathway?hsa05410/hsa:1605%09red/hsa:1756%09red |
| Rheumatoid arthritis | KEGG PATHWAY | hsa05323 | 2 | 91 | 0.038603 | 0.17658 | CCL3L1\|FOS | http://www.genome.jp/kegg-bin/show_pathway?hsa05323/hsa:2353%09red/hsa:6349%09red |
| Ubiquinone and other terpenoid-quinone biosynthesis | KEGG PATHWAY | hsa00130 | 1 | 11 | 0.039207 | 0.17658 | VKORC1 | http://www.genome.jp/kegg-bin/show_pathway?hsa00130/hsa:79001%09red |
| Human cytomegalovirus infection | KEGG PATHWAY | hsa05163 | 3 | 225 | 0.040885 | 0.17658 | CREB3L2\|CCL4L2\|CCL3L1 | http://www.genome.jp/kegg-bin/show_pathway?hsa05163/hsa:64764%09red/hsa:9560%09red/hsa:6349%09red |
| Dilated cardiomyopathy (DCM) | KEGG PATHWAY | hsa05414 | 2 | 96 | 0.042434 | 0.17658 | DMD\|DAG1 | http://www.genome.jp/kegg-bin/show_pathway?hsa05414/hsa:1605%09red/hsa:1756%09red |
| Ras signaling pathway | KEGG PATHWAY | hsa04014 | 3 | 232 | 0.044053 | 0.17658 | IGF2\|NGFR\|RAP1B | http://www.genome.jp/kegg-bin/show_pathway?hsa04014/hsa:5908%09red/hsa:4804%09red/hsa:3481%09red |
| Viral protein interaction with cytokine and cytokine receptor | KEGG PATHWAY | hsa04061 | 2 | 100 | 0.045597 | 0.17658 | CCL4L2\|CCL3L1 | http://www.genome.jp/kegg-bin/show_pathway?hsa04061/hsa:9560%09red/hsa:6349%09red |
| NF-kappa B signaling pathway | KEGG PATHWAY | hsa04064 | 2 | 100 | 0.045597 | 0.17658 | LY96\|CCL4L2 | http://www.genome.jp/kegg-bin/show_pathway?hsa04064/hsa:9560%09red/hsa:23643%09red |
| Melanogenesis | KEGG PATHWAY | hsa04916 | 2 | 101 | 0.046401 | 0.17658 | CREB3L2\|WNT5A | http://www.genome.jp/kegg-bin/show_pathway?hsa04916/hsa:64764%09red/hsa:7474%09red |
| Chagas disease (American trypanosomiasis) | KEGG PATHWAY | hsa05142 | 2 | 103 | 0.048024 | 0.177819 | CCL3L1\|FOS | http://www.genome.jp/kegg-bin/show_pathway?hsa05142/hsa:2353%09red/hsa:6349%09red |
| Parathyroid hormone synthesis, secretion and action | KEGG PATHWAY | hsa04928 | 2 | 106 | 0.050498 | 0.182058 | CREB3L2\|FOS | http://www.genome.jp/kegg-bin/show_pathway?hsa04928/hsa:64764%09red/hsa:2353%09red |
| Cholinergic synapse | KEGG PATHWAY | hsa04725 | 2 | 112 | 0.055579 | 0.190359 | CREB3L2\|FOS | http://www.genome.jp/kegg-bin/show_pathway?hsa04725/hsa:64764%09red/hsa:2353%09red |
| TNF signaling pathway | KEGG PATHWAY | hsa04668 | 2 | 112 | 0.055579 | 0.190359 | CREB3L2\|FOS | http://www.genome.jp/kegg-bin/show_pathway?hsa04668/hsa:64764%09red/hsa:2353%09red |
| Relaxin signaling pathway | KEGG PATHWAY | hsa04926 | 2 | 130 | 0.071816 | 0.237333 | CREB3L2\|FOS | http://www.genome.jp/kegg-bin/show_pathway?hsa04926/hsa:64764%09red/hsa:2353%09red |
| Dopaminergic synapse | KEGG PATHWAY | hsa04728 | 2 | 131 | 0.072759 | 0.237333 | CREB3L2\|FOS | http://www.genome.jp/kegg-bin/show_pathway?hsa04728/hsa:64764%09red/hsa:2353%09red |
| Estrogen signaling pathway | KEGG PATHWAY | hsa04915 | 2 | 138 | 0.079468 | 0.253189 | CREB3L2\|FOS | http://www.genome.jp/kegg-bin/show_pathway?hsa04915/hsa:64764%09red/hsa:2353%09red |
| Signaling pathways regulating pluripotency of stem cells | KEGG PATHWAY | hsa04550 | 2 | 140 | 0.081419 | 0.25351 | WNT5A\|ID1 | http://www.genome.jp/kegg-bin/show_pathway?hsa04550/hsa:3397%09red/hsa:7474%09red |
| Breast cancer | KEGG PATHWAY | hsa05224 | 2 | 147 | 0.088363 | 0.269017 | WNT5A\|FOS | http://www.genome.jp/kegg-bin/show_pathway?hsa05224/hsa:7474%09red/hsa:2353%09red |
| Gastric cancer | KEGG PATHWAY | hsa05226 | 2 | 149 | 0.090379 | 0.269172 | WNT5A\|TERT | http://www.genome.jp/kegg-bin/show_pathway?hsa05226/hsa:7474%09red/hsa:7015%09red |
| Hepatitis C | KEGG PATHWAY | hsa05160 | 2 | 155 | 0.096506 | 0.281305 | YWHAB\|RSAD2 | http://www.genome.jp/kegg-bin/show_pathway?hsa05160/hsa:7529%09red/hsa:91543%09red |
| Mucin type O-glycan biosynthesis | KEGG PATHWAY | hsa00512 | 1 | 31 | 0.10119 | 0.288812 | GCNT1 | http://www.genome.jp/kegg-bin/show_pathway?hsa00512/hsa:2650%09red |
| Apoptosis - multiple species | KEGG PATHWAY | hsa04215 | 1 | 33 | 0.107165 | 0.299625 | NGFR | http://www.genome.jp/kegg-bin/show_pathway?hsa04215/hsa:4804%09red |
| SNARE interactions in vesicular transport | KEGG PATHWAY | hsa04130 | 1 | 34 | 0.110138 | 0.301779 | STX6 | http://www.genome.jp/kegg-bin/show_pathway?hsa04130/hsa:10228%09red |
| Nicotine addiction | KEGG PATHWAY | hsa05033 | 1 | 40 | 0.127771 | 0.342091 | GABRA3 | http://www.genome.jp/kegg-bin/show_pathway?hsa05033/hsa:2556%09red |
| Transcriptional misregulation in cancer | KEGG PATHWAY | hsa05202 | 2 | 186 | 0.129845 | 0.342091 | IGFBP3\|NGFR | http://www.genome.jp/kegg-bin/show_pathway?hsa05202/hsa:4804%09red/hsa:3486%09red |
| Tryptophan metabolism | KEGG PATHWAY | hsa00380 | 1 | 42 | 0.133571 | 0.344908 | KYNU | http://www.genome.jp/kegg-bin/show_pathway?hsa00380/hsa:8942%09red |
| Huntington disease | KEGG PATHWAY | hsa05016 | 2 | 193 | 0.137705 | 0.344908 | CREB3L2\|MT-ATP6 | http://www.genome.jp/kegg-bin/show_pathway?hsa05016/hsa:64764%09red/hsa:4508%09red |
| Basal transcription factors | KEGG PATHWAY | hsa03022 | 1 | 45 | 0.1422 | 0.344908 | GTF2H4 | http://www.genome.jp/kegg-bin/show_pathway?hsa03022/hsa:2968%09red |
| Focal adhesion | KEGG PATHWAY | hsa04510 | 2 | 199 | 0.144525 | 0.344908 | COL6A3\|RAP1B | http://www.genome.jp/kegg-bin/show_pathway?hsa04510/hsa:5908%09red/hsa:1293%09red |
| Proteoglycans in cancer | KEGG PATHWAY | hsa05205 | 2 | 203 | 0.14911 | 0.344908 | IGF2\|WNT5A | http://www.genome.jp/kegg-bin/show_pathway?hsa05205/hsa:7474%09red/hsa:3481%09red |
| Intestinal immune network for IgA production | KEGG PATHWAY | hsa04672 | 1 | 49 | 0.153572 | 0.344908 | IL15RA | http://www.genome.jp/kegg-bin/show_pathway?hsa04672/hsa:3601%09red |
| Cocaine addiction | KEGG PATHWAY | hsa05030 | 1 | 49 | 0.153572 | 0.344908 | CREB3L2 | http://www.genome.jp/kegg-bin/show_pathway?hsa05030/hsa:64764%09red |
| Cysteine and methionine metabolism | KEGG PATHWAY | hsa00270 | 1 | 49 | 0.153572 | 0.344908 | ADI1 | http://www.genome.jp/kegg-bin/show_pathway?hsa00270/hsa:55256%09red |
| Malaria | KEGG PATHWAY | hsa05144 | 1 | 49 | 0.153572 | 0.344908 | GYPC | http://www.genome.jp/kegg-bin/show_pathway?hsa05144/hsa:2995%09red |
| Pathogenic Escherichia coli infection | KEGG PATHWAY | hsa05130 | 1 | 55 | 0.170351 | 0.37642 | LY96 | http://www.genome.jp/kegg-bin/show_pathway?hsa05130/hsa:23643%09red |
| Thermogenesis | KEGG PATHWAY | hsa04714 | 2 | 231 | 0.18194 | 0.392306 | CREB3L2\|MT-ATP6 | http://www.genome.jp/kegg-bin/show_pathway?hsa04714/hsa:64764%09red/hsa:4508%09red |
| Steroid hormone biosynthesis | KEGG PATHWAY | hsa00140 | 1 | 60 | 0.184081 | 0.392306 | AKR1C1 | http://www.genome.jp/kegg-bin/show_pathway?hsa00140/hsa:1645%09red |
| Basal cell carcinoma | KEGG PATHWAY | hsa05217 | 1 | 63 | 0.19221 | 0.392306 | WNT5A | http://www.genome.jp/kegg-bin/show_pathway?hsa05217/hsa:7474%09red |
| Cytosolic DNA-sensing pathway | KEGG PATHWAY | hsa04623 | 1 | 63 | 0.19221 | 0.392306 | CCL4L2 | http://www.genome.jp/kegg-bin/show_pathway?hsa04623/hsa:9560%09red |
| Endocytosis | KEGG PATHWAY | hsa04144 | 2 | 244 | 0.197529 | 0.392306 | CAPZA1\|ACAP1 | http://www.genome.jp/kegg-bin/show_pathway?hsa04144/hsa:9744%09red/hsa:829%09red |
| Cortisol synthesis and secretion | KEGG PATHWAY | hsa04927 | 1 | 65 | 0.197585 | 0.392306 | CREB3L2 | http://www.genome.jp/kegg-bin/show_pathway?hsa04927/hsa:64764%09red |
| Mitophagy - animal | KEGG PATHWAY | hsa04137 | 1 | 65 | 0.197585 | 0.392306 | BNIP3L | http://www.genome.jp/kegg-bin/show_pathway?hsa04137/hsa:665%09red |
| Long-term potentiation | KEGG PATHWAY | hsa04720 | 1 | 67 | 0.202924 | 0.395737 | RAP1B | http://www.genome.jp/kegg-bin/show_pathway?hsa04720/hsa:5908%09red |
| Renal cell carcinoma | KEGG PATHWAY | hsa05211 | 1 | 69 | 0.208228 | 0.395737 | RAP1B | http://www.genome.jp/kegg-bin/show_pathway?hsa05211/hsa:5908%09red |
| Central carbon metabolism in cancer | KEGG PATHWAY | hsa05230 | 1 | 69 | 0.208228 | 0.395737 | SCO2 | http://www.genome.jp/kegg-bin/show_pathway?hsa05230/hsa:9997%09red |
| Prolactin signaling pathway | KEGG PATHWAY | hsa04917 | 1 | 70 | 0.210867 | 0.395737 | FOS | http://www.genome.jp/kegg-bin/show_pathway?hsa04917/hsa:2353%09red |
| p53 signaling pathway | KEGG PATHWAY | hsa04115 | 1 | 72 | 0.216119 | 0.398987 | IGFBP3 | http://www.genome.jp/kegg-bin/show_pathway?hsa04115/hsa:3486%09red |
| Thyroid hormone synthesis | KEGG PATHWAY | hsa04918 | 1 | 74 | 0.221336 | 0.398987 | CREB3L2 | http://www.genome.jp/kegg-bin/show_pathway?hsa04918/hsa:64764%09red |
| Leishmaniasis | KEGG PATHWAY | hsa05140 | 1 | 74 | 0.221336 | 0.398987 | FOS | http://www.genome.jp/kegg-bin/show_pathway?hsa05140/hsa:2353%09red |
| Metabolism of xenobiotics by cytochrome P450 | KEGG PATHWAY | hsa00980 | 1 | 76 | 0.226518 | 0.399958 | AKR1C1 | http://www.genome.jp/kegg-bin/show_pathway?hsa00980/hsa:1645%09red |
| B cell receptor signaling pathway | KEGG PATHWAY | hsa04662 | 1 | 82 | 0.241861 | 0.399958 | FOS | http://www.genome.jp/kegg-bin/show_pathway?hsa04662/hsa:2353%09red |
| Taste transduction | KEGG PATHWAY | hsa04742 | 1 | 83 | 0.244389 | 0.399958 | GABRA3 | http://www.genome.jp/kegg-bin/show_pathway?hsa04742/hsa:2556%09red |
| Colorectal cancer | KEGG PATHWAY | hsa05210 | 1 | 86 | 0.251922 | 0.399958 | FOS | http://www.genome.jp/kegg-bin/show_pathway?hsa05210/hsa:2353%09red |
| Insulin secretion | KEGG PATHWAY | hsa04911 | 1 | 86 | 0.251922 | 0.399958 | CREB3L2 | http://www.genome.jp/kegg-bin/show_pathway?hsa04911/hsa:64764%09red |
| PD-L1 expression and PD-1 checkpoint pathway in cancer | KEGG PATHWAY | hsa05235 | 1 | 89 | 0.25938 | 0.399958 | FOS | http://www.genome.jp/kegg-bin/show_pathway?hsa05235/hsa:2353%09red |
| GABAergic synapse | KEGG PATHWAY | hsa04727 | 1 | 89 | 0.25938 | 0.399958 | GABRA3 | http://www.genome.jp/kegg-bin/show_pathway?hsa04727/hsa:2556%09red |
| Longevity regulating pathway | KEGG PATHWAY | hsa04211 | 1 | 89 | 0.25938 | 0.399958 | CREB3L2 | http://www.genome.jp/kegg-bin/show_pathway?hsa04211/hsa:64764%09red |
| Protein digestion and absorption | KEGG PATHWAY | hsa04974 | 1 | 90 | 0.26185 | 0.399958 | COL6A3 | http://www.genome.jp/kegg-bin/show_pathway?hsa04974/hsa:1293%09red |
| Morphine addiction | KEGG PATHWAY | hsa05032 | 1 | 91 | 0.264311 | 0.399958 | GABRA3 | http://www.genome.jp/kegg-bin/show_pathway?hsa05032/hsa:2556%09red |
| MicroRNAs in cancer | KEGG PATHWAY | hsa05206 | 2 | 299 | 0.26476 | 0.399958 | MIR222\|MIR137 | http://www.genome.jp/kegg-bin/show_pathway?hsa05206/hsa:407007%09red/hsa:406928%09red |
| Th1 and Th2 cell differentiation | KEGG PATHWAY | hsa04658 | 1 | 92 | 0.266765 | 0.399958 | FOS | http://www.genome.jp/kegg-bin/show_pathway?hsa04658/hsa:2353%09red |
| IL-17 signaling pathway | KEGG PATHWAY | hsa04657 | 1 | 93 | 0.26921 | 0.399958 | FOS | http://www.genome.jp/kegg-bin/show_pathway?hsa04657/hsa:2353%09red |
| TGF-beta signaling pathway | KEGG PATHWAY | hsa04350 | 1 | 94 | 0.271647 | 0.399958 | ID1 | http://www.genome.jp/kegg-bin/show_pathway?hsa04350/hsa:3397%09red |
| Prostate cancer | KEGG PATHWAY | hsa05215 | 1 | 97 | 0.27891 | 0.399958 | CREB3L2 | http://www.genome.jp/kegg-bin/show_pathway?hsa05215/hsa:64764%09red |
| Circadian entrainment | KEGG PATHWAY | hsa04713 | 1 | 97 | 0.27891 | 0.399958 | FOS | http://www.genome.jp/kegg-bin/show_pathway?hsa04713/hsa:2353%09red |
| Glycerophospholipid metabolism | KEGG PATHWAY | hsa00564 | 1 | 97 | 0.27891 | 0.399958 | PLA2G15 | http://www.genome.jp/kegg-bin/show_pathway?hsa00564/hsa:23659%09red |
| Pancreatic secretion | KEGG PATHWAY | hsa04972 | 1 | 98 | 0.281315 | 0.399958 | RAP1B | http://www.genome.jp/kegg-bin/show_pathway?hsa04972/hsa:5908%09red |
| Aldosterone synthesis and secretion | KEGG PATHWAY | hsa04925 | 1 | 98 | 0.281315 | 0.399958 | CREB3L2 | http://www.genome.jp/kegg-bin/show_pathway?hsa04925/hsa:64764%09red |
| Endocrine resistance | KEGG PATHWAY | hsa01522 | 1 | 98 | 0.281315 | 0.399958 | FOS | http://www.genome.jp/kegg-bin/show_pathway?hsa01522/hsa:2353%09red |
| Choline metabolism in cancer | KEGG PATHWAY | hsa05231 | 1 | 99 | 0.283712 | 0.399958 | FOS | http://www.genome.jp/kegg-bin/show_pathway?hsa05231/hsa:2353%09red |
| Inflammatory mediator regulation of TRP channels | KEGG PATHWAY | hsa04750 | 1 | 100 | 0.286101 | 0.399958 | ASIC1 | http://www.genome.jp/kegg-bin/show_pathway?hsa04750/hsa:41%09red |
| T cell receptor signaling pathway | KEGG PATHWAY | hsa04660 | 1 | 103 | 0.293222 | 0.405771 | FOS | http://www.genome.jp/kegg-bin/show_pathway?hsa04660/hsa:2353%09red |
| Glucagon signaling pathway | KEGG PATHWAY | hsa04922 | 1 | 106 | 0.300271 | 0.409566 | CREB3L2 | http://www.genome.jp/kegg-bin/show_pathway?hsa04922/hsa:64764%09red |
| Th17 cell differentiation | KEGG PATHWAY | hsa04659 | 1 | 107 | 0.302606 | 0.409566 | FOS | http://www.genome.jp/kegg-bin/show_pathway?hsa04659/hsa:2353%09red |
| Insulin resistance | KEGG PATHWAY | hsa04931 | 1 | 108 | 0.304932 | 0.409566 | CREB3L2 | http://www.genome.jp/kegg-bin/show_pathway?hsa04931/hsa:64764%09red |
| Neuroactive ligand-receptor interaction | KEGG PATHWAY | hsa04080 | 2 | 338 | 0.312649 | 0.412892 | KISS1\|GABRA3 | http://www.genome.jp/kegg-bin/show_pathway?hsa04080/hsa:3814%09red/hsa:2556%09red |
| Leukocyte transendothelial migration | KEGG PATHWAY | hsa04670 | 1 | 112 | 0.314162 | 0.412892 | RAP1B | http://www.genome.jp/kegg-bin/show_pathway?hsa04670/hsa:5908%09red |
| Toxoplasmosis | KEGG PATHWAY | hsa05145 | 1 | 113 | 0.31645 | 0.412892 | LY96 | http://www.genome.jp/kegg-bin/show_pathway?hsa05145/hsa:23643%09red |
| AMPK signaling pathway | KEGG PATHWAY | hsa04152 | 1 | 120 | 0.332258 | 0.424858 | CREB3L2 | http://www.genome.jp/kegg-bin/show_pathway?hsa04152/hsa:64764%09red |
| Yersinia infection | KEGG PATHWAY | hsa05135 | 1 | 121 | 0.334486 | 0.424858 | FOS | http://www.genome.jp/kegg-bin/show_pathway?hsa05135/hsa:2353%09red |
| Lysosome | KEGG PATHWAY | hsa04142 | 1 | 123 | 0.338921 | 0.424858 | PLA2G15 | http://www.genome.jp/kegg-bin/show_pathway?hsa04142/hsa:23659%09red |
| Cell cycle | KEGG PATHWAY | hsa04110 | 1 | 124 | 0.341127 | 0.424858 | YWHAB | http://www.genome.jp/kegg-bin/show_pathway?hsa04110/hsa:7529%09red |
| Platelet activation | KEGG PATHWAY | hsa04611 | 1 | 124 | 0.341127 | 0.424858 | RAP1B | http://www.genome.jp/kegg-bin/show_pathway?hsa04611/hsa:5908%09red |
| Osteoclast differentiation | KEGG PATHWAY | hsa04380 | 1 | 128 | 0.34988 | 0.426251 | FOS | http://www.genome.jp/kegg-bin/show_pathway?hsa04380/hsa:2353%09red |
| Oocyte meiosis | KEGG PATHWAY | hsa04114 | 1 | 128 | 0.34988 | 0.426251 | YWHAB | http://www.genome.jp/kegg-bin/show_pathway?hsa04114/hsa:7529%09red |
| Natural killer cell mediated cytotoxicity | KEGG PATHWAY | hsa04650 | 1 | 131 | 0.356368 | 0.426251 | MTCP1 | http://www.genome.jp/kegg-bin/show_pathway?hsa04650/hsa:4068%09red |
| Vascular smooth muscle contraction | KEGG PATHWAY | hsa04270 | 1 | 132 | 0.358517 | 0.426251 | ACTG2 | http://www.genome.jp/kegg-bin/show_pathway?hsa04270/hsa:72%09red |
| Oxidative phosphorylation | KEGG PATHWAY | hsa00190 | 1 | 133 | 0.360658 | 0.426251 | MT-ATP6 | http://www.genome.jp/kegg-bin/show_pathway?hsa00190/hsa:4508%09red |
| Spliceosome | KEGG PATHWAY | hsa03040 | 1 | 135 | 0.36492 | 0.426251 | HNRNPA1L2 | http://www.genome.jp/kegg-bin/show_pathway?hsa03040/hsa:144983%09red |
| Apoptosis | KEGG PATHWAY | hsa04210 | 1 | 136 | 0.36704 | 0.426251 | FOS | http://www.genome.jp/kegg-bin/show_pathway?hsa04210/hsa:2353%09red |
| Insulin signaling pathway | KEGG PATHWAY | hsa04910 | 1 | 137 | 0.369153 | 0.426251 | RHOQ | http://www.genome.jp/kegg-bin/show_pathway?hsa04910/hsa:23433%09red |
| Measles | KEGG PATHWAY | hsa05162 | 1 | 138 | 0.37126 | 0.426251 | FOS | http://www.genome.jp/kegg-bin/show_pathway?hsa05162/hsa:2353%09red |
| Fluid shear stress and atherosclerosis | KEGG PATHWAY | hsa05418 | 1 | 139 | 0.373359 | 0.426251 | FOS | http://www.genome.jp/kegg-bin/show_pathway?hsa05418/hsa:2353%09red |
| Parkinson disease | KEGG PATHWAY | hsa05012 | 1 | 142 | 0.379615 | 0.429812 | MT-ATP6 | http://www.genome.jp/kegg-bin/show_pathway?hsa05012/hsa:4508%09red |
| Cell adhesion molecules (CAMs) | KEGG PATHWAY | hsa04514 | 1 | 146 | 0.38786 | 0.435276 | MPZL1 | http://www.genome.jp/kegg-bin/show_pathway?hsa04514/hsa:9019%09red |
| Retrograde endocannabinoid signaling | KEGG PATHWAY | hsa04723 | 1 | 148 | 0.391942 | 0.435276 | GABRA3 | http://www.genome.jp/kegg-bin/show_pathway?hsa04723/hsa:2556%09red |
| Adrenergic signaling in cardiomyocytes | KEGG PATHWAY | hsa04261 | 1 | 149 | 0.393972 | 0.435276 | CREB3L2 | http://www.genome.jp/kegg-bin/show_pathway?hsa04261/hsa:64764%09red |
| Oxytocin signaling pathway | KEGG PATHWAY | hsa04921 | 1 | 153 | 0.402028 | 0.437126 | FOS | http://www.genome.jp/kegg-bin/show_pathway?hsa04921/hsa:2353%09red |
| mTOR signaling pathway | KEGG PATHWAY | hsa04150 | 1 | 153 | 0.402028 | 0.437126 | WNT5A | http://www.genome.jp/kegg-bin/show_pathway?hsa04150/hsa:7474%09red |
| Cellular senescence | KEGG PATHWAY | hsa04218 | 1 | 160 | 0.41587 | 0.448616 | IGFBP3 | http://www.genome.jp/kegg-bin/show_pathway?hsa04218/hsa:3486%09red |
| Jak-STAT signaling pathway | KEGG PATHWAY | hsa04630 | 1 | 162 | 0.419767 | 0.449062 | IL15RA | http://www.genome.jp/kegg-bin/show_pathway?hsa04630/hsa:3601%09red |
| Protein processing in endoplasmic reticulum | KEGG PATHWAY | hsa04141 | 1 | 165 | 0.425563 | 0.449062 | TXNDC5 | http://www.genome.jp/kegg-bin/show_pathway?hsa04141/hsa:81567%09red |
| cGMP-PKG signaling pathway | KEGG PATHWAY | hsa04022 | 1 | 167 | 0.429395 | 0.449062 | CREB3L2 | http://www.genome.jp/kegg-bin/show_pathway?hsa04022/hsa:64764%09red |
| Influenza A | KEGG PATHWAY | hsa05164 | 1 | 167 | 0.429395 | 0.449062 | RSAD2 | http://www.genome.jp/kegg-bin/show_pathway?hsa05164/hsa:91543%09red |
| Alzheimer disease | KEGG PATHWAY | hsa05010 | 1 | 171 | 0.436983 | 0.453536 | MT-ATP6 | http://www.genome.jp/kegg-bin/show_pathway?hsa05010/hsa:4508%09red |
| Alcoholism | KEGG PATHWAY | hsa05034 | 1 | 180 | 0.453693 | 0.465717 | CREB3L2 | http://www.genome.jp/kegg-bin/show_pathway?hsa05034/hsa:64764%09red |
| Axon guidance | KEGG PATHWAY | hsa04360 | 1 | 181 | 0.455518 | 0.465717 | WNT5A | http://www.genome.jp/kegg-bin/show_pathway?hsa04360/hsa:7474%09red |
| Kaposi sarcoma-associated herpesvirus infection | KEGG PATHWAY | hsa05167 | 1 | 186 | 0.464558 | 0.47144 | FOS | http://www.genome.jp/kegg-bin/show_pathway?hsa05167/hsa:2353%09red |
| Human immunodeficiency virus 1 infection | KEGG PATHWAY | hsa05170 | 1 | 212 | 0.509215 | 0.51296 | FOS | http://www.genome.jp/kegg-bin/show_pathway?hsa05170/hsa:2353%09red |
| Metabolic pathways | KEGG PATHWAY | hsa01100 | 5 | 1433 | 0.523759 | 0.523759 | GCNT1\|MT-ATP6\|KYNU\|VKORC1\|ADI1 | http://www.genome.jp/kegg-bin/show_pathway?hsa01100/hsa:2650%09red/hsa:55256%09red/hsa:4508%09red/hsa:8942%09red/hsa:79001%09red |
|  |  |  |  |  |  |  |  |  |
| -------------------- |  |  |  |  |  |  |  |  |
|  |  |  |  |  |  |  |  |  |
| #Term | Database | ID | Input number | Background number | P-Value | Corrected P-Value | Input | Hyperlink |
|  |  |  |  |  |  |  |  |  |
| -------------------- |  |  |  |  |  |  |  |  |
|  |  |  |  |  |  |  |  |  |
| #Term | Database | ID | Input number | Background number | P-Value | Corrected P-Value | Input | Hyperlink |
|  |  |  |  |  |  |  |  |  |
| -------------------- |  |  |  |  |  |  |  |  |
