# Supplementary material for: Identification of downstream targets and signaling pathways of long non-coding RNA NR_002794 in human trophoblast cells
Source: Bioengineered. 2021 Sep 13;12(1):6617–28. doi: 10.1080/21655979.2021.1974808 (PMC8806843; doi:10.1080/21655979.2021.1974808)
Supplement: Supplemental Material [file KBIE_A_1974808_SM0255.zip › supplementary/supplementary Table 5.docx]

| **Genes that were markedly up-regulated in KD versus NC group and notably down-regulated in OE versus NC group** | | | | | | | | | | |
| --- | --- | --- | --- | --- | --- | --- | --- | --- | --- | --- |
|  |  | KD vs NC | | | |  | OE vs NC | | | |
| Gene id | GeneName | log2FoldChange | pValue | qValue | result |  | log2FoldChange | pValue | qValue | result |
| ENSG00000175274 | TP53I11 | 2.641542 | 0 | 0 | up |  | -2.01584 | 4.74E-53 | 1.4E-51 | down |
| ENSG00000111348 | ARHGDIB | 2.091057 | 0 | 0 | up |  | -2.94271 | 3.6E-125 | 2.7E-123 | down |
| ENSG00000250748 | AC025419.1 | 1.869915 | 2.74E-66 | 9.3E-65 | up |  | -1.14347 | 2.38E-11 | 1.66E-10 | down |
| ENSG00000136732 | GYPC | 1.690875 | 9.1E-105 | 5.1E-103 | up |  | -1.24425 | 2.14E-24 | 2.94E-23 | down |
| ENSG00000134321 | RSAD2 | 1.59906 | 2.9E-147 | 2.4E-145 | up |  | -1.21439 | 2.18E-29 | 3.6E-28 | down |
| ENSG00000170498 | KISS1 | 1.524842 | 1.8E-61 | 5.62E-60 | up |  | -1.45733 | 2.08E-17 | 2.1E-16 | down |
| ENSG00000102934 | PLLP | 1.408618 | 9.9E-159 | 9E-157 | up |  | -1.76484 | 5.42E-56 | 1.7E-54 | down |
| ENSG00000008517 | IL32 | 1.368975 | 1.13E-16 | 1.03E-15 | up |  | -1.55198 | 1.09E-11 | 7.71E-11 | down |
| ENSG00000066056 | TIE1 | 1.259655 | 1.49E-69 | 5.36E-68 | up |  | -1.37687 | 8.49E-44 | 2.05E-42 | down |
| ENSG00000114315 | HES1 | 1.139091 | 1.26E-18 | 1.25E-17 | up |  | -4.67362 | 1.7E-41 | 3.86E-40 | down |
| ENSG00000276070 | CCL4L2 | 1.130783 | 4.84E-61 | 1.5E-59 | up |  | -1.43123 | 6.66E-54 | 2E-52 | down |
| ENSG00000198947 | DMD | 1.111455 | 6.76E-37 | 1.26E-35 | up |  | -1.55096 | 7.98E-48 | 2.16E-46 | down |
| ENSG00000134470 | IL15RA | 1.060832 | 2.28E-34 | 3.95E-33 | up |  | -1.2425 | 1.39E-31 | 2.45E-30 | down |
| ENSG00000011677 | GABRA3 | 1.027181 | 5.06E-29 | 7.42E-28 | up |  | -2.78554 | 4.16E-72 | 1.7E-70 | down |
