# Supplementary material for: Identification of downstream targets and signaling pathways of long non-coding RNA NR_002794 in human trophoblast cells
Source: Bioengineered. 2021 Sep 13;12(1):6617–28. doi: 10.1080/21655979.2021.1974808 (PMC8806843; doi:10.1080/21655979.2021.1974808)
Supplement: Supplemental Material [file KBIE_A_1974808_SM0255.zip › supplementary/supplementary Table 6.docx]

| Genes that were markedly down-regulated in KD versus NC group and notably up-regulated in OE versus NC group | | | | | | | | | | | |
| --- | --- | --- | --- | --- | --- | --- | --- | --- | --- | --- | --- |
|  | KD vs NC | | | | |  |  | OE vs NC | | | |
| Gene id | GeneName | log2FoldChange | pValue | qValue | result |  |  | log2FoldChange | pValue | qValue | result |
| ENSG00000107984 | DKK1 | -2.16654 | 0 | 0 | down |  |  | 1.223646 | 0 | 0 | up |
| ENSG00000229124 | VIM-AS1 | -2.13288 | 3.57E-45 | 8.18E-44 | down |  |  | 1.461491 | 6.69E-89 | 3.52E-87 | up |
| ENSG00000139292 | LGR5 | -2.10924 | 1.82E-43 | 3.98E-42 | down |  |  | 1.230436 | 1.5E-61 | 5.19E-60 | up |
| ENSG00000187210 | GCNT1 | -2.04598 | 8.09E-21 | 8.84E-20 | down |  |  | 1.293838 | 1.7E-160 | 1.7E-158 | up |
| ENSG00000229563 | LINC01204 | -1.85336 | 4.99E-12 | 3.47E-11 | down |  |  | 1.029267 | 2.15E-07 | 1.07E-06 | up |
| ENSG00000258667 | HIF1A-AS2 | -1.80085 | 2.61E-06 | 1.13E-05 | down |  |  | 1.14621 | 1.41E-07 | 7.17E-07 | up |
| ENSG00000093100 | AC016026.1 | -1.73537 | 1.01E-34 | 1.77E-33 | down |  |  | 1.086875 | 1.01E-40 | 2.25E-39 | up |
| ENSG00000106484 | MEST | -1.66446 | 1.67E-46 | 3.93E-45 | down |  |  | 1.576068 | 1.3E-151 | 1.2E-149 | up |
| ENSG00000267523 | AC008735.2 | -1.57245 | 7.27E-06 | 3E-05 | down |  |  | 1.132463 | 4.02E-08 | 2.17E-07 | up |
