# Supplementary material for: Identification of downstream targets and signaling pathways of long non-coding RNA NR_002794 in human trophoblast cells
Source: Bioengineered. 2021 Sep 13;12(1):6617–28. doi: 10.1080/21655979.2021.1974808 (PMC8806843; doi:10.1080/21655979.2021.1974808)
Supplement: Supplemental Material [file KBIE_A_1974808_SM0255.zip › supplementary/supplementary Table 7.docx]

| **Annotation analysis for genes in Supplementary Table 3** | | | |
| --- | --- | --- | --- |
| //// |  |  |  |
| Query: | TP53I11 |  |  |
| Gene: | hsa:9537 | TP53I11, PIG11 | |
| Entrez Gene ID: | 9537 |  |  |
| Disease: | PSYCH | GAD |  |
|  | Amyotrophic lateral sclerosis (sporadic) | NHGRI GWAS Catalog | |
|  | Stomach cancer | FunDO | 1841 |
|  | ATTENTION DEFICIT DISORDER WITH HYPERACTIVITY | GAD |  |
| GO: | negative regulation of cell population proliferation | Gene Ontology | GO:0008285 |
|  | integral component of membrane | Gene Ontology | GO:0016021 |
| GOslim: | response to stress | Gene Ontology Slim | GO:0006950 |
| //// |  |  |  |
| Query: | ARHGDIB |  |  |
| Gene: | hsa:397 | ARHGDIB, D4, GDIA2, GDID4, LYGDI, Ly-GDI, RAP1GN1, RhoGDI2 | |
| Entrez Gene ID: | 397 |  |  |
| Pathway: | Caspase Cascade in Apoptosis | PID | caspase_pathway |
|  | Rho GTPase cycle | Reactome | R-HSA-194840 |
|  | caspase cascade in apoptosis | BioCarta | 100218 |
|  | Neurotrophin signaling pathway | KEGG PATHWAY | hsa04722 |
|  | hiv-1 nef: negative effector of fas and tnf | BioCarta | 100144 |
|  | d4gdi signaling pathway | BioCarta | 100191 |
|  | Regulation of RhoA activity | PID | rhoa_reg_pathway |
|  | Vasopressin-regulated water reabsorption | KEGG PATHWAY | hsa04962 |
|  | Signaling by Rho GTPases | Reactome | R-HSA-194315 |
|  | Signal Transduction | Reactome | R-HSA-162582 |
| Disease: | Cancer | FunDO | 1934 |
| GO: | GTPase activity | Gene Ontology | GO:0003924 |
|  | Rho GDP-dissociation inhibitor activity | Gene Ontology | GO:0005094 |
|  | GTPase activator activity | Gene Ontology | GO:0005096 |
|  | protein binding | Gene Ontology | GO:0005515 |
|  | cytoplasm | Gene Ontology | GO:0005737 |
|  | cytosol | Gene Ontology | GO:0005829 |
|  | cytoskeleton | Gene Ontology | GO:0005856 |
|  | negative regulation of cell adhesion | Gene Ontology | GO:0007162 |
|  | Rho protein signal transduction | Gene Ontology | GO:0007266 |
|  | multicellular organism development | Gene Ontology | GO:0007275 |
|  | membrane | Gene Ontology | GO:0016020 |
|  | cytoplasmic vesicle | Gene Ontology | GO:0031410 |
|  | regulation of Rho protein signal transduction | Gene Ontology | GO:0035023 |
|  | positive regulation of GTPase activity | Gene Ontology | GO:0043547 |
|  | Rac GTPase binding | Gene Ontology | GO:0048365 |
|  | regulation of small GTPase mediated signal transduction | Gene Ontology | GO:0051056 |
|  | extracellular exosome | Gene Ontology | GO:0070062 |
|  | cellular response to redox state | Gene Ontology | GO:0071461 |
|  | negative regulation of trophoblast cell migration | Gene Ontology | GO:1901164 |
|  | regulation of actin cytoskeleton reorganization | Gene Ontology | GO:2000249 |
| GOslim: | GTPase activity | Gene Ontology Slim | GO:0003924 |
|  | intracellular | Gene Ontology Slim | GO:0005622 |
|  | cytoplasm | Gene Ontology Slim | GO:0005737 |
|  | cytosol | Gene Ontology Slim | GO:0005829 |
|  | cytoskeleton | Gene Ontology Slim | GO:0005856 |
|  | organelle | Gene Ontology Slim | GO:0043226 |
| //// |  |  |  |
| Query: | AC025419.1 | |  |
| //// |  |  |  |
| Query: | GYPC |  |  |
| Gene: | hsa:2995 | GYPC, CD236, CD236R, GE, GE:GPC:GPD:GYPD, GPC, GPD, GYPD, PAS-2, PAS-2' | |
| Entrez Gene ID: | 2995 |  |  |
| Pathway: | Malaria | KEGG PATHWAY | hsa05144 |
|  | Hemostasis | Reactome | R-HSA-109582 |
|  | Cell surface interactions at the vascular wall | Reactome | R-HSA-202733 |
| Disease: | CARDIOVASCULAR | GAD |  |
|  | METABOLIC | GAD |  |
|  | HEMATOLOGICAL | GAD |  |
|  | INFECTION | GAD |  |
|  | Intelligence | NHGRI GWAS Catalog | |
|  | HEART FAILURE | GAD |  |
|  | Blood group, Gerbich | OMIM | 616089 |
|  | OVALOCYTOSIS AND MALARIA SUSCEPTIBILITY | GAD |  |
|  | Malaria, resistance to | OMIM | 611162 |
|  | ERYTHROCYTE COUNT | GAD |  |
|  | HEMOGLOBINS | GAD |  |
|  | SODIUM | GAD |  |
|  | TUNICA MEDIA | GAD |  |
|  | WAIST-HIP RATIO | GAD |  |
| GO: | protein binding | Gene Ontology | GO:0005515 |
|  | plasma membrane | Gene Ontology | GO:0005886 |
|  | integral component of plasma membrane | Gene Ontology | GO:0005887 |
|  | membrane | Gene Ontology | GO:0016020 |
|  | cortical cytoskeleton | Gene Ontology | GO:0030863 |
|  | leukocyte migration | Gene Ontology | GO:0050900 |
| GOslim: | plasma membrane | Gene Ontology Slim | GO:0005886 |
| //// |  |  |  |
| Query: | RSAD2 |  |  |
| Gene: | hsa:91543 | RSAD2, 2510004L01Rik, cig33, cig5, vig1 | |
| Entrez Gene ID: | 91543 |  |  |
| Pathway: | Influenza A | KEGG PATHWAY | hsa05164 |
|  | Hepatitis C | KEGG PATHWAY | hsa05160 |
|  | Immune System | Reactome | R-HSA-168256 |
|  | Interferon alpha/beta signaling | Reactome | R-HSA-909733 |
|  | Interferon Signaling | Reactome | R-HSA-913531 |
|  | Cytokine Signaling in Immune system | Reactome | R-HSA-1280215 |
| GO: | fibrillar center | Gene Ontology | GO:0001650 |
|  | catalytic activity | Gene Ontology | GO:0003824 |
|  | protein binding | Gene Ontology | GO:0005515 |
|  | mitochondrion | Gene Ontology | GO:0005739 |
|  | mitochondrial outer membrane | Gene Ontology | GO:0005741 |
|  | mitochondrial inner membrane | Gene Ontology | GO:0005743 |
|  | endoplasmic reticulum | Gene Ontology | GO:0005783 |
|  | endoplasmic reticulum membrane | Gene Ontology | GO:0005789 |
|  | Golgi apparatus | Gene Ontology | GO:0005794 |
|  | lipid droplet | Gene Ontology | GO:0005811 |
|  | response to virus | Gene Ontology | GO:0009615 |
|  | viral process | Gene Ontology | GO:0016032 |
|  | positive regulation of toll-like receptor 7 signaling pathway | Gene Ontology | GO:0034157 |
|  | positive regulation of toll-like receptor 9 signaling pathway | Gene Ontology | GO:0034165 |
|  | CD4-positive, alpha-beta T cell activation | Gene Ontology | GO:0035710 |
|  | CD4-positive, alpha-beta T cell differentiation | Gene Ontology | GO:0043367 |
|  | protein self-association | Gene Ontology | GO:0043621 |
|  | negative regulation of viral genome replication | Gene Ontology | GO:0045071 |
|  | metal ion binding | Gene Ontology | GO:0046872 |
|  | negative regulation of protein secretion | Gene Ontology | GO:0050709 |
|  | positive regulation of immune response | Gene Ontology | GO:0050778 |
|  | 4 iron, 4 sulfur cluster binding | Gene Ontology | GO:0051539 |
|  | defense response to virus | Gene Ontology | GO:0051607 |
|  | type I interferon signaling pathway | Gene Ontology | GO:0060337 |
|  | positive regulation of T-helper 2 cell cytokine production | Gene Ontology | GO:2000553 |
| GOslim: | intracellular | Gene Ontology Slim | GO:0005622 |
|  | cytoplasm | Gene Ontology Slim | GO:0005737 |
|  | mitochondrion | Gene Ontology Slim | GO:0005739 |
|  | endoplasmic reticulum | Gene Ontology Slim | GO:0005783 |
|  | Golgi apparatus | Gene Ontology Slim | GO:0005794 |
|  | lipid droplet | Gene Ontology Slim | GO:0005811 |
|  | organelle | Gene Ontology Slim | GO:0043226 |
| //// |  |  |  |
| Query: | KISS1 |  |  |
| Gene: | hsa:3814 | KISS1, HH13, KiSS-1 | |
| Entrez Gene ID: | 3814 |  |  |
| Pathway: | Neuroactive ligand-receptor interaction | KEGG PATHWAY | hsa04080 |
|  | Class A/1 (Rhodopsin-like receptors) | Reactome | R-HSA-373076 |
|  | GPCR downstream signalling | Reactome | R-HSA-388396 |
|  | Signaling by GPCR | Reactome | R-HSA-372790 |
|  | Peptide ligand-binding receptors | Reactome | R-HSA-375276 |
|  | Signal Transduction | Reactome | R-HSA-162582 |
|  | GPCR ligand binding | Reactome | R-HSA-500792 |
|  | G alpha (q) signalling events | Reactome | R-HSA-416476 |
| Disease: | METABOLIC | GAD |  |
|  | POLYCYSTIC OVARY SYNDROME | GAD |  |
|  | OBESITY | GAD |  |
|  | Cancer | FunDO | 1934 |
|  | Endocrine and metabolic diseases | KEGG DISEASE | |
|  | Hypothalamus and pituitary gland diseases | KEGG DISEASE | |
|  | Hypogonadotropic hypogonadism | KEGG DISEASE | H00255 |
|  | POLYCYSTIC OVARIAN SYNDROME | GAD |  |
|  | Hypogonadotropic hypogonadism 13 with or without anosmia | OMIM | 614842 |
|  | NATURAL MENOPAUSE | GAD |  |
|  | POF - PREMATURE OVARIAN FAILURE | GAD |  |
|  | PRIMARY OVARIAN INSUFFICIENCY | GAD |  |
|  | PUBERTY, DELAYED | GAD |  |
|  | PUBERTY, PRECOCIOUS | GAD |  |
|  | MENARCH | GAD |  |
| GO: | protein binding | Gene Ontology | GO:0005515 |
|  | extracellular region | Gene Ontology | GO:0005576 |
|  | extracellular space | Gene Ontology | GO:0005615 |
|  | cytoplasm | Gene Ontology | GO:0005737 |
|  | cytoskeleton organization | Gene Ontology | GO:0007010 |
|  | G protein-coupled receptor signaling pathway | Gene Ontology | GO:0007186 |
|  | positive regulation of cytosolic calcium ion concentration | Gene Ontology | GO:0007204 |
|  | negative regulation of cell population proliferation | Gene Ontology | GO:0008285 |
|  | apical plasma membrane | Gene Ontology | GO:0016324 |
|  | kisspeptin receptor binding | Gene Ontology | GO:0031773 |
|  | positive regulation of luteinizing hormone secretion | Gene Ontology | GO:0033686 |
|  | neuron projection | Gene Ontology | GO:0043005 |
|  | neuronal cell body | Gene Ontology | GO:0043025 |
|  | positive regulation of MAPK cascade | Gene Ontology | GO:0043410 |
|  | positive regulation of synaptic transmission | Gene Ontology | GO:0050806 |
|  | positive regulation of cytosolic calcium ion concentration involved in phospholipase C-activating G protein-coupled signaling pathway | Gene Ontology | GO:0051482 |
|  | generation of ovulation cycle rhythm | Gene Ontology | GO:0060112 |
|  | positive regulation of growth hormone secretion | Gene Ontology | GO:0060124 |
| GOslim: | extracellular region | Gene Ontology Slim | GO:0005576 |
|  | extracellular space | Gene Ontology Slim | GO:0005615 |
|  | intracellular | Gene Ontology Slim | GO:0005622 |
|  | cytoplasm | Gene Ontology Slim | GO:0005737 |
|  | cytosol | Gene Ontology Slim | GO:0005829 |
|  | cytoskeleton organization | Gene Ontology Slim | GO:0007010 |
| //// |  |  |  |
| Query: | PLLP |  |  |
| Gene: | hsa:51090 | PLLP, PMLP, TM4SF11 | |
| Entrez Gene ID: | 51090 |  |  |
| Disease: | NEUROLOGICAL | GAD |  |
|  | Sleep duration | NHGRI GWAS Catalog | |
|  | ALZHEIMER DISEASE | GAD |  |
| GO: | protein binding | Gene Ontology | GO:0005515 |
|  | ion transport | Gene Ontology | GO:0006811 |
|  | response to wounding | Gene Ontology | GO:0009611 |
|  | integral component of membrane | Gene Ontology | GO:0016021 |
|  | structural constituent of myelin sheath | Gene Ontology | GO:0019911 |
|  | myelination | Gene Ontology | GO:0042552 |
|  | compact myelin | Gene Ontology | GO:0043218 |
|  | membrane raft | Gene Ontology | GO:0045121 |
| //// |  |  |  |
| Query: | IL32 |  |  |
| Gene: | hsa:9235 | IL32, IL-32alpha, IL-32beta, IL-32delta, IL-32gamma, NK4, TAIF, TAIFa, TAIFb, TAIFc, TAIFd | |
| Entrez Gene ID: | 9235 |  |  |
| Pathway: | Cytokine-cytokine receptor interaction | KEGG PATHWAY | hsa04060 |
|  | Immune System | Reactome | R-HSA-168256 |
|  | Other interleukin signaling | Reactome | R-HSA-449836 |
|  | Cytokine Signaling in Immune system | Reactome | R-HSA-1280215 |
|  | Signaling by Interleukins | Reactome | R-HSA-449147 |
| Disease: | INFECTION | GAD |  |
|  | Rheumatoid arthritis | FunDO | 1781 |
|  | HIV-1 susceptibility | NHGRI GWAS Catalog | |
|  | Autoimmune disease | FunDO | 1807 |
|  | HIV-1 | GAD |  |
| GO: | cytokine activity | Gene Ontology | GO:0005125 |
|  | protein binding | Gene Ontology | GO:0005515 |
|  | extracellular space | Gene Ontology | GO:0005615 |
|  | cytosol | Gene Ontology | GO:0005829 |
|  | defense response | Gene Ontology | GO:0006952 |
|  | immune response | Gene Ontology | GO:0006955 |
|  | cell adhesion | Gene Ontology | GO:0007155 |
|  | membrane | Gene Ontology | GO:0016020 |
|  | cytokine-mediated signaling pathway | Gene Ontology | GO:0019221 |
| GOslim: | extracellular region | Gene Ontology Slim | GO:0005576 |
|  | extracellular space | Gene Ontology Slim | GO:0005615 |
|  | intracellular | Gene Ontology Slim | GO:0005622 |
|  | cytoplasm | Gene Ontology Slim | GO:0005737 |
|  | cytosol | Gene Ontology Slim | GO:0005829 |
|  | cell adhesion | Gene Ontology Slim | GO:0007155 |
| //// |  |  |  |
| Query: | TIE1 |  |  |
| Gene: | hsa:7075 | TIE1, JTK14, TIE | |
| Entrez Gene ID: | 7075 |  |  |
| Disease: | Neurilemmoma | FunDO | 1771 |
|  | Fibroid tumor | FunDO | 1864 |
|  | Gastrointestinal stromal tumor | FunDO | 2033 |
| GO: | angiogenesis | Gene Ontology | GO:0001525 |
|  | vasculogenesis | Gene Ontology | GO:0001570 |
|  | in utero embryonic development | Gene Ontology | GO:0001701 |
|  | transmembrane receptor protein tyrosine kinase activity | Gene Ontology | GO:0004714 |
|  | protein binding | Gene Ontology | GO:0005515 |
|  | ATP binding | Gene Ontology | GO:0005524 |
|  | integral component of plasma membrane | Gene Ontology | GO:0005887 |
|  | signal transduction | Gene Ontology | GO:0007165 |
|  | transmembrane receptor protein tyrosine kinase signaling pathway | Gene Ontology | GO:0007169 |
|  | multicellular organism development | Gene Ontology | GO:0007275 |
|  | mesoderm development | Gene Ontology | GO:0007498 |
|  | negative regulation of angiogenesis | Gene Ontology | GO:0016525 |
|  | peptidyl-tyrosine phosphorylation | Gene Ontology | GO:0018108 |
|  | negative regulation of cell migration | Gene Ontology | GO:0030336 |
|  | response to retinoic acid | Gene Ontology | GO:0032526 |
|  | positive regulation of kinase activity | Gene Ontology | GO:0033674 |
|  | receptor complex | Gene Ontology | GO:0043235 |
|  | plasma membrane fusion | Gene Ontology | GO:0045026 |
|  | positive regulation of angiogenesis | Gene Ontology | GO:0045766 |
| GOslim: | signal transduction | Gene Ontology Slim | GO:0007165 |
| //// |  |  |  |
| Query: | HES1 |  |  |
| //// |  |  |  |
| Query: | CCL4L2 |  |  |
| Gene: | hsa:9560 | CCL4L2, AT744.2, CCL4L, SCYA4L, SCYQ4L2 | |
| Entrez Gene ID: | 9560 |  |  |
| Pathway: | NF-kappa B signaling pathway | KEGG PATHWAY | hsa04064 |
|  | Inflammation mediated by chemokine and cytokine signaling pathway | PANTHER | P00031 |
|  | Cytokine-cytokine receptor interaction | KEGG PATHWAY | hsa04060 |
|  | Viral protein interaction with cytokine and cytokine receptor | KEGG PATHWAY | hsa04061 |
|  | Chemokine signaling pathway | KEGG PATHWAY | hsa04062 |
|  | Toll-like receptor signaling pathway | KEGG PATHWAY | hsa04620 |
|  | Cytosolic DNA-sensing pathway | KEGG PATHWAY | hsa04623 |
|  | Salmonella infection | KEGG PATHWAY | hsa05132 |
|  | Human cytomegalovirus infection | KEGG PATHWAY | hsa05163 |
|  | GPCR downstream signalling | Reactome | R-HSA-388396 |
|  | Signaling by GPCR | Reactome | R-HSA-372790 |
|  | Signal Transduction | Reactome | R-HSA-162582 |
|  | G alpha (i) signalling events | Reactome | R-HSA-418594 |
| Disease: | HIV infection | FunDO | 1809 |
|  | Protein quantitative trait loci | NHGRI GWAS Catalog | |
| GOslim: | extracellular region | Gene Ontology Slim | GO:0005576 |
|  | extracellular space | Gene Ontology Slim | GO:0005615 |
| //// |  |  |  |
| Query: | DMD |  |  |
| Gene: | hsa:1756 | DMD, BMD, CMD3B, DXS142, DXS164, DXS206, DXS230, DXS239, DXS268, DXS269, DXS270, DXS272, MRX85 | |
| Entrez Gene ID: | 1756 |  |  |
| Pathway: | Dilated cardiomyopathy (DCM) | KEGG PATHWAY | hsa05414 |
|  | Viral myocarditis | KEGG PATHWAY | hsa05416 |
|  | agrin in postsynaptic differentiation | BioCarta | 100252 |
|  | Extracellular matrix organization | Reactome | R-HSA-1474244 |
|  | Hypertrophic cardiomyopathy (HCM) | KEGG PATHWAY | hsa05410 |
|  | Arrhythmogenic right ventricular cardiomyopathy (ARVC) | KEGG PATHWAY | hsa05412 |
|  | Non-integrin membrane-ECM interactions | Reactome | R-HSA-3000171 |
|  | Striated Muscle Contraction | Reactome | R-HSA-390522 |
|  | Muscle contraction | Reactome | R-HSA-397014 |
| Disease: | NEUROLOGICAL | GAD |  |
|  | CARDIOVASCULAR | GAD |  |
|  | METABOLIC | GAD |  |
|  | HEMATOLOGICAL | GAD |  |
|  | AGING | GAD |  |
|  | PSYCH | GAD |  |
|  | BIPOLAR DISORDER | GAD |  |
|  | INSULIN | GAD |  |
|  | Anxiety in major depressive disorder | NHGRI GWAS Catalog | |
|  | PHARMACOGENOMIC | GAD |  |
|  | CHOLESTEROL, HDL | GAD |  |
|  | OSTEOPOROSIS | GAD |  |
|  | Cardiovascular diseases | KEGG DISEASE | |
|  | Cardiac diseases | KEGG DISEASE | |
|  | Dilated cardiomyopathy | KEGG DISEASE | H00294 |
|  | CHOLESTEROL, LDL | GAD |  |
|  | C-REACTIVE PROTEIN | GAD |  |
|  | Response to antidepressant treatment | NHGRI GWAS Catalog | |
|  | Musculoskeletal diseases | KEGG DISEASE | |
|  | Muscular diseases | KEGG DISEASE | |
|  | Dystrophinopathies | KEGG DISEASE | H00562 |
|  | Duchenne muscular dystrophy | KEGG DISEASE | H01963 |
|  | Becker muscular dystrophy | KEGG DISEASE | H01964 |
|  | BODY MASS INDEX | GAD |  |
|  | BODY WEIGHT | GAD |  |
|  | Duchenne muscular dystrophy | OMIM | 310200 |
|  | MUSCULAR DYSTROPHY | GAD |  |
|  | DUCHENNE MUSCULAR DYSTROPHY | GAD |  |
|  | X-LINKED DILATED CARDIOMYOPATHY | GAD |  |
|  | DILATED CARDIOMYOPATHY | GAD |  |
|  | MEMORY | GAD |  |
|  | AMYOTROPHIC LATERAL SCLEROSIS | GAD |  |
|  | Becker muscular dystrophy | OMIM | 300376 |
|  | HEMATOCRIT | GAD |  |
|  | AUDIOMETRY, PURE-TONE | GAD |  |
|  | ERYTHROCYTES | GAD |  |
|  | EXERCISE TEST | GAD |  |
|  | HIP | GAD |  |
|  | OCULAR PHYSIOLOGICAL PHENOMENA | GAD |  |
|  | LIPOPROTEINS, VLDL | GAD |  |
|  | ANTIDEPRESSIVE AGENTS | GAD |  |
|  | Cardiomyopathy, dilated, 3B | OMIM | 302045 |
| GO: | regulation of heart rate | Gene Ontology | GO:0002027 |
|  | dystroglycan binding | Gene Ontology | GO:0002162 |
|  | actin binding | Gene Ontology | GO:0003779 |
|  | structural constituent of cytoskeleton | Gene Ontology | GO:0005200 |
|  | protein binding | Gene Ontology | GO:0005515 |
|  | cytosol | Gene Ontology | GO:0005829 |
|  | cytoskeleton | Gene Ontology | GO:0005856 |
|  | cytoskeleton organization | Gene Ontology | GO:0007010 |
|  | muscle organ development | Gene Ontology | GO:0007517 |
|  | zinc ion binding | Gene Ontology | GO:0008270 |
|  | structural constituent of muscle | Gene Ontology | GO:0008307 |
|  | cell surface | Gene Ontology | GO:0009986 |
|  | regulation of release of sequestered calcium ion into cytosol by sarcoplasmic reticulum | Gene Ontology | GO:0010880 |
|  | regulation of cardiac muscle contraction by regulation of the release of sequestered calcium ion | Gene Ontology | GO:0010881 |
|  | regulation of skeletal muscle contraction by regulation of release of sequestered calcium ion | Gene Ontology | GO:0014809 |
|  | regulation of skeletal muscle contraction | Gene Ontology | GO:0014819 |
|  | dystrophin-associated glycoprotein complex | Gene Ontology | GO:0016010 |
|  | syntrophin complex | Gene Ontology | GO:0016013 |
|  | lateral plasma membrane | Gene Ontology | GO:0016328 |
|  | myosin binding | Gene Ontology | GO:0017022 |
|  | vinculin binding | Gene Ontology | GO:0017166 |
|  | Z disc | Gene Ontology | GO:0030018 |
|  | muscle filament sliding | Gene Ontology | GO:0030049 |
|  | cell-substrate junction | Gene Ontology | GO:0030055 |
|  | filopodium | Gene Ontology | GO:0030175 |
|  | filopodium membrane | Gene Ontology | GO:0031527 |
|  | protein-containing complex | Gene Ontology | GO:0032991 |
|  | negative regulation of peptidyl-serine phosphorylation | Gene Ontology | GO:0033137 |
|  | cellular protein localization | Gene Ontology | GO:0034613 |
|  | cellular protein-containing complex assembly | Gene Ontology | GO:0034622 |
|  | maintenance of blood-brain barrier | Gene Ontology | GO:0035633 |
|  | response to muscle stretch | Gene Ontology | GO:0035994 |
|  | sarcolemma | Gene Ontology | GO:0042383 |
|  | costamere | Gene Ontology | GO:0043034 |
|  | peptide biosynthetic process | Gene Ontology | GO:0043043 |
|  | neuron projection terminus | Gene Ontology | GO:0044306 |
|  | membrane raft | Gene Ontology | GO:0045121 |
|  | synapse | Gene Ontology | GO:0045202 |
|  | postsynaptic membrane | Gene Ontology | GO:0045211 |
|  | muscle cell cellular homeostasis | Gene Ontology | GO:0046716 |
|  | muscle fiber development | Gene Ontology | GO:0048747 |
|  | nitric-oxide synthase binding | Gene Ontology | GO:0050998 |
|  | cardiac muscle contraction | Gene Ontology | GO:0060048 |
|  | regulation of ryanodine-sensitive calcium-release channel activity | Gene Ontology | GO:0060314 |
|  | cardiac muscle cell action potential | Gene Ontology | GO:0086001 |
|  | regulation of voltage-gated calcium channel activity | Gene Ontology | GO:1901385 |
|  | negative regulation of peptidyl-cysteine S-nitrosylation | Gene Ontology | GO:1902083 |
|  | positive regulation of sodium ion transmembrane transporter activity | Gene Ontology | GO:2000651 |
| GOslim: | intracellular | Gene Ontology Slim | GO:0005622 |
|  | nucleus | Gene Ontology Slim | GO:0005634 |
|  | cytoplasm | Gene Ontology Slim | GO:0005737 |
|  | cytosol | Gene Ontology Slim | GO:0005829 |
|  | cytoskeleton | Gene Ontology Slim | GO:0005856 |
|  | plasma membrane | Gene Ontology Slim | GO:0005886 |
|  | organelle | Gene Ontology Slim | GO:0043226 |
| //// |  |  |  |
| Query: | IL15RA |  |  |
| Gene: | hsa:3601 | IL15RA, CD215 | |
| Entrez Gene ID: | 3601 |  |  |
| Pathway: | Intestinal immune network for IgA production | KEGG PATHWAY | hsa04672 |
|  | Cytokine-cytokine receptor interaction | KEGG PATHWAY | hsa04060 |
|  | Interleukin signaling pathway | PANTHER | P00036 |
|  | Human T-cell leukemia virus 1 infection | KEGG PATHWAY | hsa05166 |
|  | Pathways in cancer | KEGG PATHWAY | hsa05200 |
|  | Jak-STAT signaling pathway | KEGG PATHWAY | hsa04630 |
|  | Immune System | Reactome | R-HSA-168256 |
|  | Cytokine Signaling in Immune system | Reactome | R-HSA-1280215 |
|  | Signaling by Interleukins | Reactome | R-HSA-449147 |
|  | Interleukin-15 signaling | Reactome | R-HSA-8983432 |
|  | Interleukin-2 family signaling | Reactome | R-HSA-451927 |
| Disease: | IMMUNE | GAD |  |
|  | Lymphoma | FunDO | 2239 |
|  | Embryoma | FunDO | 1848 |
|  | Asthma (bronchodilator response) | NHGRI GWAS Catalog | |
|  | Inflammatory bowel disease | NHGRI GWAS Catalog | |
|  | INFLAMMATION | GAD |  |
| GO: | Golgi membrane | Gene Ontology | GO:0000139 |
|  | cytokine receptor activity | Gene Ontology | GO:0004896 |
|  | protein binding | Gene Ontology | GO:0005515 |
|  | extracellular space | Gene Ontology | GO:0005615 |
|  | endosome | Gene Ontology | GO:0005768 |
|  | endoplasmic reticulum membrane | Gene Ontology | GO:0005789 |
|  | plasma membrane | Gene Ontology | GO:0005886 |
|  | cell surface | Gene Ontology | GO:0009986 |
|  | integral component of membrane | Gene Ontology | GO:0016021 |
|  | protein kinase binding | Gene Ontology | GO:0019901 |
|  | cytoplasmic vesicle membrane | Gene Ontology | GO:0030659 |
|  | nuclear membrane | Gene Ontology | GO:0031965 |
|  | interleukin-15-mediated signaling pathway | Gene Ontology | GO:0035723 |
|  | interleukin-15 receptor activity | Gene Ontology | GO:0042010 |
|  | positive regulation of phagocytosis | Gene Ontology | GO:0050766 |
| GOslim: | extracellular region | Gene Ontology Slim | GO:0005576 |
|  | extracellular space | Gene Ontology Slim | GO:0005615 |
|  | plasma membrane | Gene Ontology Slim | GO:0005886 |
|  | signal transduction | Gene Ontology Slim | GO:0007165 |
|  | cell population proliferation | Gene Ontology Slim | GO:0008283 |
| //// |  |  |  |
| Query: | GABRA3 |  |  |
| Gene: | hsa:2556 | GABRA3 |  |
| Entrez Gene ID: | 2556 |  |  |
| Pathway: | cardiac protection against ros | BioCarta | 100163 |
|  | Neuroactive ligand-receptor interaction | KEGG PATHWAY | hsa04080 |
|  | Retrograde endocannabinoid signaling | KEGG PATHWAY | hsa04723 |
|  | gamma-aminobutyric acid receptor life cycle pathway | BioCarta | 100158 |
|  | Taste transduction | KEGG PATHWAY | hsa04742 |
|  | Morphine addiction | KEGG PATHWAY | hsa05032 |
|  | GABAergic synapse | KEGG PATHWAY | hsa04727 |
|  | Nicotine addiction | KEGG PATHWAY | hsa05033 |
|  | Neuronal System | Reactome | R-HSA-112316 |
|  | GABA receptor activation | Reactome | R-HSA-977443 |
|  | Transmission across Chemical Synapses | Reactome | R-HSA-112315 |
|  | Neurotransmitter receptors and postsynaptic signal transmission | Reactome | R-HSA-112314 |
| Disease: | IMMUNE | GAD |  |
|  | PSYCH | GAD |  |
|  | DEPRESSIVE DISORDER, MAJOR | GAD |  |
|  | MULTIPLE SCLEROSIS | GAD |  |
| GO: | GABA-A receptor activity | Gene Ontology | GO:0004890 |
|  | inhibitory extracellular ligand-gated ion channel activity | Gene Ontology | GO:0005237 |
|  | chloride channel activity | Gene Ontology | GO:0005254 |
|  | protein binding | Gene Ontology | GO:0005515 |
|  | plasma membrane | Gene Ontology | GO:0005886 |
|  | integral component of plasma membrane | Gene Ontology | GO:0005887 |
|  | signal transduction | Gene Ontology | GO:0007165 |
|  | gamma-aminobutyric acid signaling pathway | Gene Ontology | GO:0007214 |
|  | chemical synaptic transmission | Gene Ontology | GO:0007268 |
|  | benzodiazepine receptor activity | Gene Ontology | GO:0008503 |
|  | GABA-gated chloride ion channel activity | Gene Ontology | GO:0022851 |
|  | neurotransmitter receptor activity | Gene Ontology | GO:0030594 |
|  | dendrite membrane | Gene Ontology | GO:0032590 |
|  | ion transmembrane transport | Gene Ontology | GO:0034220 |
|  | chloride channel complex | Gene Ontology | GO:0034707 |
|  | regulation of membrane potential | Gene Ontology | GO:0042391 |
|  | neuron projection | Gene Ontology | GO:0043005 |
|  | synapse | Gene Ontology | GO:0045202 |
|  | nervous system process | Gene Ontology | GO:0050877 |
|  | synaptic transmission, GABAergic | Gene Ontology | GO:0051932 |
|  | regulation of postsynaptic membrane potential | Gene Ontology | GO:0060078 |
|  | postsynapse | Gene Ontology | GO:0098794 |
|  | GABA-ergic synapse | Gene Ontology | GO:0098982 |
|  | integral component of postsynaptic specialization membrane | Gene Ontology | GO:0099060 |
|  | chloride transmembrane transport | Gene Ontology | GO:1902476 |
|  | GABA-A receptor complex | Gene Ontology | GO:1902711 |
|  | transmitter-gated ion channel activity involved in regulation of postsynaptic membrane potential | Gene Ontology | GO:1904315 |
| GOslim: | plasma membrane | Gene Ontology Slim | GO:0005886 |
|  | transport | Gene Ontology Slim | GO:0006810 |
| //// |  |  |  |
| //// |  |  |  |
| Query: | DKK1 |  |  |
| Gene: | hsa:22943 | DKK1, DKK-1, SK | |
| Entrez Gene ID: | 22943 |  |  |
| Pathway: | Wnt signaling network | PID | wnt_signaling_pathway |
|  | inactivation of gsk3 by akt causes accumulation of b-catenin in alveolar macrophages | BioCarta | 100152 |
|  | Regulation of nuclear beta catenin signaling and target gene transcription | PID | betacatenin_nuc_pathway |
|  | Validated targets of C-MYC transcriptional repression | PID | myc_represspathway |
|  | wnt signaling pathway | BioCarta | 100002 |
|  | segmentation clock | BioCarta | 100146 |
|  | Wnt signaling pathway | KEGG PATHWAY | hsa04310 |
|  | Presenilin action in Notch and Wnt signaling | PID | ps1pathway |
|  | multi-step regulation of transcription by pitx2 | BioCarta | 100074 |
|  | Direct p53 effectors | PID | p53downstreampathway |
|  | wnt lrp6 signalling | BioCarta | 100003 |
|  | Signaling by WNT | Reactome | R-HSA-195721 |
|  | Signaling by WNT in cancer | Reactome | R-HSA-4791275 |
|  | Signal Transduction | Reactome | R-HSA-162582 |
|  | Negative regulation of TCF-dependent signaling by WNT ligand antagonists | Reactome | R-HSA-3772470 |
|  | Diseases of signal transduction | Reactome | R-HSA-5663202 |
|  | TCF dependent signaling in response to WNT | Reactome | R-HSA-201681 |
|  | Disease | Reactome | R-HSA-1643685 |
|  | Misspliced LRP5 mutants have enhanced beta-catenin-dependent signaling | Reactome | R-HSA-5339717 |
| Disease: | NEUROLOGICAL | GAD |  |
|  | METABOLIC | GAD |  |
|  | Bone properties (heel) | NHGRI GWAS Catalog | |
|  | Periodontitis (Mean PAL) | NHGRI GWAS Catalog | |
|  | QRS duration | NHGRI GWAS Catalog | |
|  | Electrocardiographic traits | NHGRI GWAS Catalog | |
|  | PHOSPHORUS | GAD |  |
|  | BRAIN | GAD |  |
| GO: | negative regulation of transcription by RNA polymerase II | Gene Ontology | GO:0000122 |
|  | cell morphogenesis involved in differentiation | Gene Ontology | GO:0000904 |
|  | endoderm formation | Gene Ontology | GO:0001706 |
|  | mesoderm formation | Gene Ontology | GO:0001707 |
|  | hair follicle development | Gene Ontology | GO:0001942 |
|  | regulation of receptor internalization | Gene Ontology | GO:0002090 |
|  | protein binding | Gene Ontology | GO:0005515 |
|  | extracellular region | Gene Ontology | GO:0005576 |
|  | extracellular space | Gene Ontology | GO:0005615 |
|  | plasma membrane | Gene Ontology | GO:0005886 |
|  | learning or memory | Gene Ontology | GO:0007611 |
|  | growth factor activity | Gene Ontology | GO:0008083 |
|  | positive regulation of gene expression | Gene Ontology | GO:0010628 |
|  | positive regulation of cell death | Gene Ontology | GO:0010942 |
|  | negative regulation of neuron projection development | Gene Ontology | GO:0010977 |
|  | negative regulation of Wnt signaling pathway | Gene Ontology | GO:0030178 |
|  | negative regulation of ossification | Gene Ontology | GO:0030279 |
|  | embryonic limb morphogenesis | Gene Ontology | GO:0030326 |
|  | negative regulation of BMP signaling pathway | Gene Ontology | GO:0030514 |
|  | forebrain development | Gene Ontology | GO:0030900 |
|  | early endosome membrane | Gene Ontology | GO:0031901 |
|  | negative regulation of protein binding | Gene Ontology | GO:0032091 |
|  | response to retinoic acid | Gene Ontology | GO:0032526 |
|  | negative regulation of peptidyl-serine phosphorylation | Gene Ontology | GO:0033137 |
|  | co-receptor binding | Gene Ontology | GO:0039706 |
|  | negative regulation of mesodermal cell fate specification | Gene Ontology | GO:0042662 |
|  | regulation of endodermal cell fate specification | Gene Ontology | GO:0042663 |
|  | negative regulation of apoptotic process | Gene Ontology | GO:0043066 |
|  | positive regulation of JUN kinase activity | Gene Ontology | GO:0043507 |
|  | positive regulation of Wnt signaling pathway, calcium modulating pathway | Gene Ontology | GO:0045813 |
|  | receptor antagonist activity | Gene Ontology | GO:0048019 |
|  | low-density lipoprotein particle receptor binding | Gene Ontology | GO:0050750 |
|  | regulation of synaptic transmission, glutamatergic | Gene Ontology | GO:0051966 |
|  | limb development | Gene Ontology | GO:0060173 |
|  | face morphogenesis | Gene Ontology | GO:0060325 |
|  | negative regulation of pathway-restricted SMAD protein phosphorylation | Gene Ontology | GO:0060394 |
|  | motor learning | Gene Ontology | GO:0061743 |
|  | positive regulation of heart induction by negative regulation of canonical Wnt signaling pathway | Gene Ontology | GO:0090082 |
|  | negative regulation of canonical Wnt signaling pathway | Gene Ontology | GO:0090090 |
|  | Wnt signaling pathway involved in somitogenesis | Gene Ontology | GO:0090244 |
|  | modulation of age-related behavioral decline | Gene Ontology | GO:0090647 |
|  | synapse pruning | Gene Ontology | GO:0098883 |
|  | positive regulation of neuron death | Gene Ontology | GO:1901216 |
|  | negative regulation of canonical Wnt signaling pathway involved in cardiac muscle cell fate commitment | Gene Ontology | GO:1901296 |
|  | positive regulation of tau-protein kinase activity | Gene Ontology | GO:1902949 |
|  | regulation of dopaminergic neuron differentiation | Gene Ontology | GO:1904338 |
|  | negative regulation of Wnt-Frizzled-LRP5/6 complex assembly | Gene Ontology | GO:1904723 |
|  | positive regulation of midbrain dopaminergic neuron differentiation | Gene Ontology | GO:1904958 |
|  | negative regulation of presynapse assembly | Gene Ontology | GO:1905607 |
|  | positive regulation of Wnt signaling pathway, planar cell polarity pathway | Gene Ontology | GO:2000096 |
|  | negative regulation of signaling receptor activity | Gene Ontology | GO:2000272 |
|  | negative regulation of cardiac muscle cell differentiation | Gene Ontology | GO:2000726 |
| GOslim: | extracellular region | Gene Ontology Slim | GO:0005576 |
|  | extracellular space | Gene Ontology Slim | GO:0005615 |
|  | plasma membrane | Gene Ontology Slim | GO:0005886 |
| //// |  |  |  |
| Query: | VIM-AS1 |  |  |
| Gene: | hsa:100507347 | VIM-AS1 |  |
| Entrez Gene ID: | 1.01E+08 |  |  |
| //// |  |  |  |
| Query: | LGR5 |  |  |
| Gene: | hsa:8549 | LGR5, FEX, GPR49, GPR67, GRP49, HG38 | |
| Entrez Gene ID: | 8549 |  |  |
| Pathway: | Wnt signaling pathway | KEGG PATHWAY | hsa04310 |
|  | Signaling by WNT | Reactome | R-HSA-195721 |
|  | Regulation of FZD by ubiquitination | Reactome | R-HSA-4641263 |
|  | Signal Transduction | Reactome | R-HSA-162582 |
|  | TCF dependent signaling in response to WNT | Reactome | R-HSA-201681 |
| Disease: | CARDIOVASCULAR | GAD |  |
|  | METABOLIC | GAD |  |
|  | DIABETES, TYPE 2 | GAD |  |
|  | BLOOD PRESSURE | GAD |  |
|  | Type 2 diabetes | NHGRI GWAS Catalog | |
|  | CORONARY ARTERY DISEASE | GAD |  |
|  | RENAL | GAD |  |
|  | TYPE 2 DIABETES | GAD |  |
|  | GLOMERULAR FILTRATION RATE | GAD |  |
|  | EXERCISE TEST | GAD |  |
| GO: | hair follicle development | Gene Ontology | GO:0001942 |
|  | transmembrane signaling receptor activity | Gene Ontology | GO:0004888 |
|  | G protein-coupled receptor activity | Gene Ontology | GO:0004930 |
|  | protein binding | Gene Ontology | GO:0005515 |
|  | plasma membrane | Gene Ontology | GO:0005886 |
|  | integral component of plasma membrane | Gene Ontology | GO:0005887 |
|  | G protein-coupled receptor signaling pathway | Gene Ontology | GO:0007186 |
|  | adenylate cyclase-activating G protein-coupled receptor signaling pathway | Gene Ontology | GO:0007189 |
|  | activation of adenylate cyclase activity | Gene Ontology | GO:0007190 |
|  | G protein-coupled peptide receptor activity | Gene Ontology | GO:0008528 |
|  | hormone-mediated signaling pathway | Gene Ontology | GO:0009755 |
|  | oocyte differentiation | Gene Ontology | GO:0009994 |
|  | protein-hormone receptor activity | Gene Ontology | GO:0016500 |
|  | trans-Golgi network membrane | Gene Ontology | GO:0032588 |
|  | regulation of cell population proliferation | Gene Ontology | GO:0042127 |
|  | inner ear development | Gene Ontology | GO:0048839 |
|  | positive regulation of canonical Wnt signaling pathway | Gene Ontology | GO:0090263 |
|  | epithelial cell proliferation involved in renal tubule morphogenesis | Gene Ontology | GO:2001013 |
| GOslim: | plasma membrane | Gene Ontology Slim | GO:0005886 |
| //// |  |  |  |
| Query: | GCNT1 |  |  |
| Gene: | hsa:2650 | GCNT1, C2GNT, C2GNT-L, C2GNT1, G6NT, NACGT2, NAGCT2 | |
| Entrez Gene ID: | 2650 |  |  |
| Pathway: | Metabolic pathways | KEGG PATHWAY | hsa01100 |
|  | mucin core 1 and core 2 O-glycosylation | BioCyc | PWY-7433 |
|  | Mucin type O-glycan biosynthesis | KEGG PATHWAY | hsa00512 |
|  | O-linked glycosylation | Reactome | R-HSA-5173105 |
|  | Metabolism of proteins | Reactome | R-HSA-392499 |
|  | Post-translational protein modification | Reactome | R-HSA-597592 |
|  | O-linked glycosylation of mucins | Reactome | R-HSA-913709 |
| Disease: | CARDIOVASCULAR | GAD |  |
|  | HYPERTENSION | GAD |  |
|  | BLOOD PRESSURE | GAD |  |
|  | Lymphoma | FunDO | 2239 |
|  | Rheumatoid arthritis | FunDO | 1781 |
|  | Prostate cancer | FunDO | 1831 |
|  | Diabetes mellitus | FunDO | 1904 |
|  | Hyperglycemia | FunDO | 2040 |
|  | Leukemia | FunDO | 2051 |
|  | Dialysis-related mortality | NHGRI GWAS Catalog | |
| GO: | Golgi membrane | Gene Ontology | GO:0000139 |
|  | beta-1,3-galactosyl-O-glycosyl-glycoprotein beta-1,6-N-acetylglucosaminyltransferase activity | Gene Ontology | GO:0003829 |
|  | protein binding | Gene Ontology | GO:0005515 |
|  | extracellular space | Gene Ontology | GO:0005615 |
|  | trans-Golgi network | Gene Ontology | GO:0005802 |
|  | glycoprotein biosynthetic process | Gene Ontology | GO:0009101 |
|  | integral component of membrane | Gene Ontology | GO:0016021 |
|  | O-glycan processing | Gene Ontology | GO:0016266 |
|  | Golgi cisterna | Gene Ontology | GO:0031985 |
|  | response to insulin | Gene Ontology | GO:0032868 |
|  | tissue morphogenesis | Gene Ontology | GO:0048729 |
|  | leukocyte tethering or rolling | Gene Ontology | GO:0050901 |
|  | cell adhesion molecule production | Gene Ontology | GO:0060352 |
|  | kidney morphogenesis | Gene Ontology | GO:0060993 |
| GOslim: | extracellular region | Gene Ontology Slim | GO:0005576 |
|  | extracellular space | Gene Ontology Slim | GO:0005615 |
| //// |  |  |  |
| Query: | LINC01204 | |  |
| Gene: | hsa:101927528 | LINC01204 | |
| Entrez Gene ID: | 1.02E+08 |  |  |
| //// |  |  |  |
| Query: | HIF1A-AS2 | |  |
| Gene: | hsa:100750247 | HIF1A-AS2, 3'aHIF-1A, aHIF | |
| Entrez Gene ID: | 1.01E+08 |  |  |
| //// |  |  |  |
| Query: | AC016026.1 | |  |
| //// |  |  |  |
| Query: | MEST |  |  |
| Gene: | hsa:4232 | MEST, PEG1 | |
| Entrez Gene ID: | 4232 |  |  |
| Disease: | Autistic disorder | FunDO | 2124 |
|  | Infiltrating cancer | FunDO | 1788 |
|  | Breast cancer | FunDO | 1944 |
| GO: | protein binding | Gene Ontology | GO:0005515 |
|  | endoplasmic reticulum | Gene Ontology | GO:0005783 |
|  | endoplasmic reticulum membrane | Gene Ontology | GO:0005789 |
|  | mesoderm development | Gene Ontology | GO:0007498 |
|  | regulation of lipid storage | Gene Ontology | GO:0010883 |
|  | integral component of membrane | Gene Ontology | GO:0016021 |
|  | hydrolase activity | Gene Ontology | GO:0016787 |
|  | extracellular exosome | Gene Ontology | GO:0070062 |
| GOslim: | intracellular | Gene Ontology Slim | GO:0005622 |
|  | cytoplasm | Gene Ontology Slim | GO:0005737 |
|  | endoplasmic reticulum | Gene Ontology Slim | GO:0005783 |
|  | organelle | Gene Ontology Slim | GO:0043226 |
| //// |  |  |  |
| Query: | AC008735.2 | |  |
| //// |  |  |  |
