# Supplementary material for: Identification of downstream targets and signaling pathways of long non-coding RNA NR_002794 in human trophoblast cells
Source: Bioengineered. 2021 Sep 13;12(1):6617–28. doi: 10.1080/21655979.2021.1974808 (PMC8806843; doi:10.1080/21655979.2021.1974808)
Supplement: Supplemental Material [file KBIE_A_1974808_SM0255.zip › supplementary/Supplementary Table legends.docx]

Supplementary Table 1 Differentially expressed genes in the KD versus NC group.

Supplementary Table 2 Differentially expressed genes in the OE versus NC group.

Supplementary Table 3 Common differentially expressed genes in both KD versus NC group and OE versus NC group.

Supplementary Table 4 KEGG enrichment analysis for genes in the Supplementary Table 1 Sheet3.

Supplementary Table 5 Genes that were markedly up-regulated in the KD versus NC group and notably down-regulated in the OE versus NC group.

Supplementary Table 6 Genes that were markedly down-regulated in the KD versus NC group and notably up-regulated in the OE versus NC group.

Supplementary Table 7 Annotation analysis for genes in Supplementary Table 5 and 6.
